# Laser Shock Peening of Gear Steels and Related Metallic Materials: Near-Surface States, Surface Integrity, and Component-Level Applications

**DOI:** 10.3390/ma19153257

**Published:** 2026-08-01

**Authors:** Yuxuan Sheng, Xin Hou, Yi Hou, Wenjie Chen, Qianjin Liu, Xiaoqiang Li, Shengguan Qu

**Affiliations:** 1 School of Mechanical and Automotive Engineering, South China University of Technology, Guangzhou 510640, China; 2 Guangdong Provincial Key Laboratory for Processing and Forming of Advanced Metallic Materials, National Engineering Research Center of Near-Net-Shape Forming for Metallic Materials, South China University of Technology, Guangzhou 510640, China; 3 KUKA Robotics Automation (Guangdong) Co., Ltd., Foshan 528300, China; 4 State Key Laboratory of High-End Heavy-Load Robots, Midea Group, Foshan 528300, China

**Keywords:** laser shock peening, gear steels, compressive residual stress, surface integrity, fatigue and wear resistance, hybrid strengthening

## Abstract

**Highlights:**

- Laser shock peening (LSP) and hybrid routes raise fatigue resistance of gear steels and related materials.
- Quality metrics include compressive residual stress (CRS) depth, hardening gradient, microstructure, topography.
- LSP discussion covers tooth flanks, root transitions, gaps, and bore constraints.

**Abstract:**

Contact fatigue, bending fatigue, and wear failures in gears are governed by the stress state, hardening gradient, microstructural stability, and surface topography within the near-surface and subsurface regions. For tooth flanks, rolling–sliding contact, asperity interaction, lubricant-film disturbance, and subsurface shear stress control micropitting, pitting, and spalling. For tooth-root fillets, local stress concentration and surface or near-surface defects dominate bending-fatigue crack initiation. Laser shock peening (LSP) introduces deep compressive residual stress, gradient hardening, and microstructural refinement, and is therefore relevant to gears when these effects are matched to the critical damage zones. This review examines LSP of gear steels and related load-bearing steels from the viewpoint of tooth-flank and tooth-root damage control. It links laser parameters, shock-induced plastic deformation, residual-stress depth, hardening response, surface roughness, and profile accuracy to bending fatigue, rolling contact fatigue, and wear behavior. LSP is most effective when the compressive residual-stress layer and hardened layer reach the contact- or bending-damage depth while lubrication compatibility, flank form accuracy, and subsequent finishing are preserved. Excessive pulse energy, overlap, or unstable absorbing/confining conditions may increase roughness, produce ablation or micropitting-sensitive defects, and compromise tooth profile accuracy. Thus, LSP for gears should be evaluated together with carburizing, nitriding, shot peening, surface rolling, polishing, coatings, and laser texturing rather than as an isolated treatment. The central task is to define gear-specific process windows that balance residual-stress depth, surface integrity, dimensional accuracy, and manufacturing repeatability.

## 1. Introduction

In high-precision and heavily loaded gear transmissions, reliability is closely tied to the quality and stability of the working surface. The tooth flank is continuously exposed to rolling–sliding contact and cyclic Hertzian stress [1,2], whereas the tooth-root transition region carries bending loads and local stress concentration [3]. Damage developing within this confined surface-to-subsurface volume changes the local stress state [4], microstructural stability, and crack-initiation behavior, and therefore has a direct effect on gear reliability in service [5]. As aerospace systems [6], industrial robots [7], precision machine tools [8], and electrified transportation move toward lightweight architectures, higher power density, heavier loads, and longer service lives [9], gear components are expected to maintain surface quality, fatigue resistance, and transmission accuracy over longer service intervals [10]. In heavily loaded precision transmissions, including RV reducers and machine-tool gearboxes [11,12], progressive near-surface damage may degrade transmission accuracy [13], increase vibration and noise [14], and eventually lead to pitting, spalling, bending fatigue, or wear failure [15]. Controlled strengthening of the surface-to-subsurface region is therefore essential for improving the reliability and service stability of precision transmission systems [16].

Common gear surface-strengthening methods include carburizing [17], nitriding [18], shot peening [19], surface rolling [20], and related hybrid treatments [21]. In practice, the strengthening of gear steels is usually built from the substrate outward. Heat treatment provides the core strength and toughness required for load support [22,23], while carburizing or nitriding forms a hardened diffusion case with a depth-dependent hardness gradient [24,25]. External mechanical treatments such as shot peening and surface rolling further modify the outer surface layer by introducing plastic deformation, work hardening, and compressive residual stress (CRS), thereby reducing crack-initiation sensitivity and altering the local stress state [26]. In gear service, contact fatigue, tooth-root bending fatigue, micropitting, wear, and subsurface spalling tend to occur at different depths and in different local regions. It is therefore difficult for a single strengthening route to fully meet the requirements of the tooth-flank friction interface, the subsurface crack-growth region, and the tooth-root stress-concentration region at the same time [27]. The fatigue benefit of surface strengthening is also governed by surface morphology, residual-stress distribution, microstructural hardening, and stress relaxation. These factors can produce different responses under high-cycle and very-high-cycle fatigue conditions [28]. Gear surface strengthening therefore requires coordinated control of the surface and subsurface layers to meet the coupled demands imposed by complex service conditions.

Laser shock peening (LSP) offers a route for near-surface strengthening of gear-related materials. In this process, a short-pulse, high-energy laser generates transient shock loading, producing high-strain-rate plastic deformation beneath the surface and establishing a relatively deep CRS field, a hardness gradient, and a refined microstructural gradient [29,30]. Because LSP is a shock-wave-driven, noncontact, high-strain-rate treatment, it is particularly useful for modifying subsurface stress fields, delaying short-crack growth, and changing the depth at which cracks initiate. These features make it relevant to the strengthening of damage-prone surface and subsurface regions in gears. In 40CrMo steel, LSP can generate a favorable near-surface CRS layer and improve overall mechanical performance [31]. In related steels such as AISI 304, repeated laser peening without coating (LSPwC) has also been reported to improve fatigue and fretting-wear behavior [32]. Through the combined action of CRS, plastic strain accumulation, gradient hardening, and microstructural refinement, LSP can suppress crack initiation and early crack growth in critical gear regions [33].

The use of LSP in gear materials nevertheless has clear limits. The strengthening response is highly sensitive to the initial material state and the combined effects of laser energy, pulse duration, spot size, overlap ratio, number of impacts, confining layer, and absorbing layer. The resulting trends are often non-monotonic and may reach saturation [34]. The tooth-flank contact region is sensitive to roughness, morphological continuity, and lubricant-film condition. Excessive energy input or instability of the absorbing layer may cause ablation, micropitting, and increased roughness, offsetting the benefit of the CRS field [35]. Complex regions such as tooth-root transitions, tooth gaps, micro-gears, and inner bores also involve curvature variation, restricted incidence, and nonuniform coverage. Parameter relationships obtained from flat specimens cannot therefore be transferred directly to real gear components without redefining the process window [36]. More specifically, the tooth-flank contact region requires control of roughness, lubrication compatibility, and coverage of the subsurface shear-stress zone. For tooth-root transitions, continuous CRS coverage and an increased crack-initiation threshold are more critical. Inner bores and tooth-gap regions are further constrained by incident angle and strengthening uniformity. During long-term service, CRS relaxation, surface morphology evolution, and coupled wear–fatigue damage may also change the effectiveness of the initially strengthened layer [37]. For gear applications, LSP must therefore be assessed against several practical questions: whether the affected depth reaches the critical damage zone, whether the cost to surface integrity remains acceptable, whether the strengthened layer is stable in service, and whether complex components can be treated with sufficient consistency.

Although LSP has been widely studied in titanium alloys, aluminum alloys, nickel-based superalloys, and aerospace components [38,39,40], its gear-specific implications have not been treated with the same coherence. Existing reviews generally discuss LSP in terms of processing variables, microstructural evolution, mechanical properties, predictive models, and broad metallic applications [41,42], or include it as one branch of laser surface treatment for gears alongside laser hardening, remelting, cladding, texturing, and hybrid laser processing [43,44]. For steel and steel-alloy gears, available studies have reported residual stress, microstructural refinement, wear, fatigue, and related responses [45,46,47,48,49,50], but these results remain fragmented. The missing link is a gear-oriented framework that connects LSP process input with near-surface stress–microstructure evolution, surface-integrity change, and failure behavior in damage-prone layers and geometrically constrained regions.

**Figure 1:**
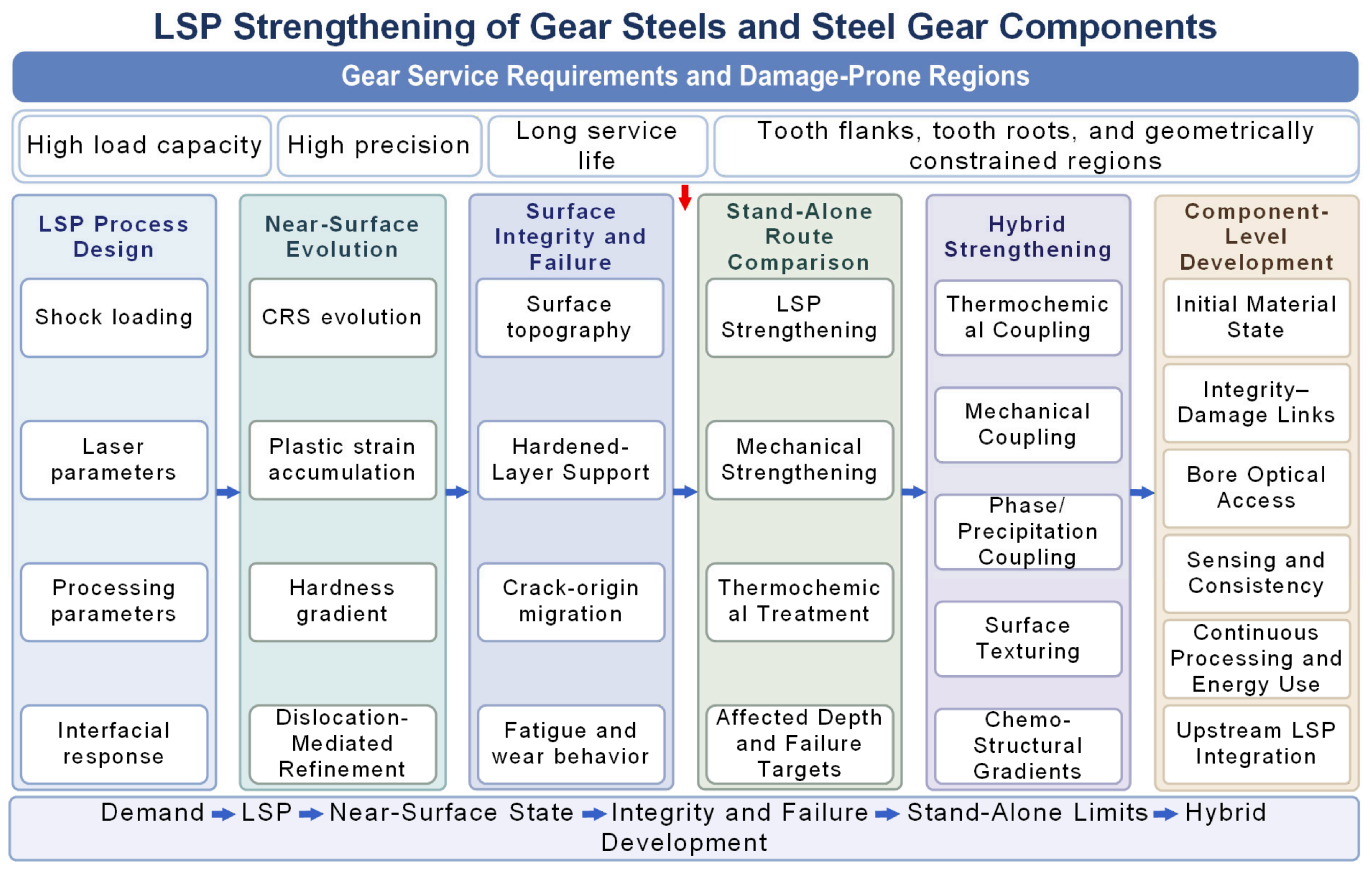
Technical framework of this review.

Because component-level studies of LSP-treated gears remain limited, this review takes a narrative, mechanism-oriented approach built around representative studies, not a systematic review or meta-analysis. Its focus is the gear-relevant chain linking process conditions, near-surface states, surface integrity, and fatigue/wear behavior. Gear components, gear steels, and gear-like specimens form the main evidence base; other steels and model materials are used selectively when they help interpret LSP mechanisms or parameter effects. Following the framework in Figure 1, the review first introduces shock-loading principles and process parameters, then examines the formation of CRS fields, hardness gradients, and refined microstructures in the near-surface region. It further links these state changes to surface integrity, fatigue and wear behavior, and compares stand-alone LSP with mechanical and thermochemical strengthening routes to define its capability limits. On this basis, hybrid strengthening strategies are discussed, and the outlook focuses on LSP adaptation to complex gear geometries, process-window identification, process monitoring, and manufacturing consistency.

## 2. Fundamental Principles and Process Parameters of Laser Shock Peening

Laser shock peening converts nanosecond laser energy into plasma-driven shock loading, near-surface plastic yielding, and retained residual stress [51]. Process design for gear-related materials must provide sufficient shock pressure, cover damage-prone regions, preserve surface integrity, and remain compatible with complex geometries. Laser energy, pulse duration, spot geometry, scanning path, overlap ratio, and number of impacts are therefore treated as coupled variables governing the strengthening response.

### 2.1. Formation of Laser-Induced Shock Loading and Near-Surface Plastic Response

The effect of LSP on the near-surface region arises from the generation and propagation of shock loading and the plastic response induced by that loading [52]. This sequence is governed by the intrinsic laser parameters, processing strategy, interfacial confinement, and the dynamic response of the material [53]. The near-surface response to LSP is governed by three coupled processes: laser–plasma shock generation, stress-wave propagation with residual-stress development, and interfacial confinement by the transparent and absorbing layers [54].

#### 2.1.1. Formation of Laser–Plasma Shock Loading

LSP typically uses high-peak-power, short-pulse lasers to deliver a high energy density to the material surface on a nanosecond time scale, meeting the transient power requirement for plasma-driven shock loading [55,56]. Its defining feature is the coupling between nanosecond energy deposition and a transient mechanical shock response, which distinguishes LSP from heat-dominated laser processes such as laser hardening and laser cladding [57].

**Figure 2:**
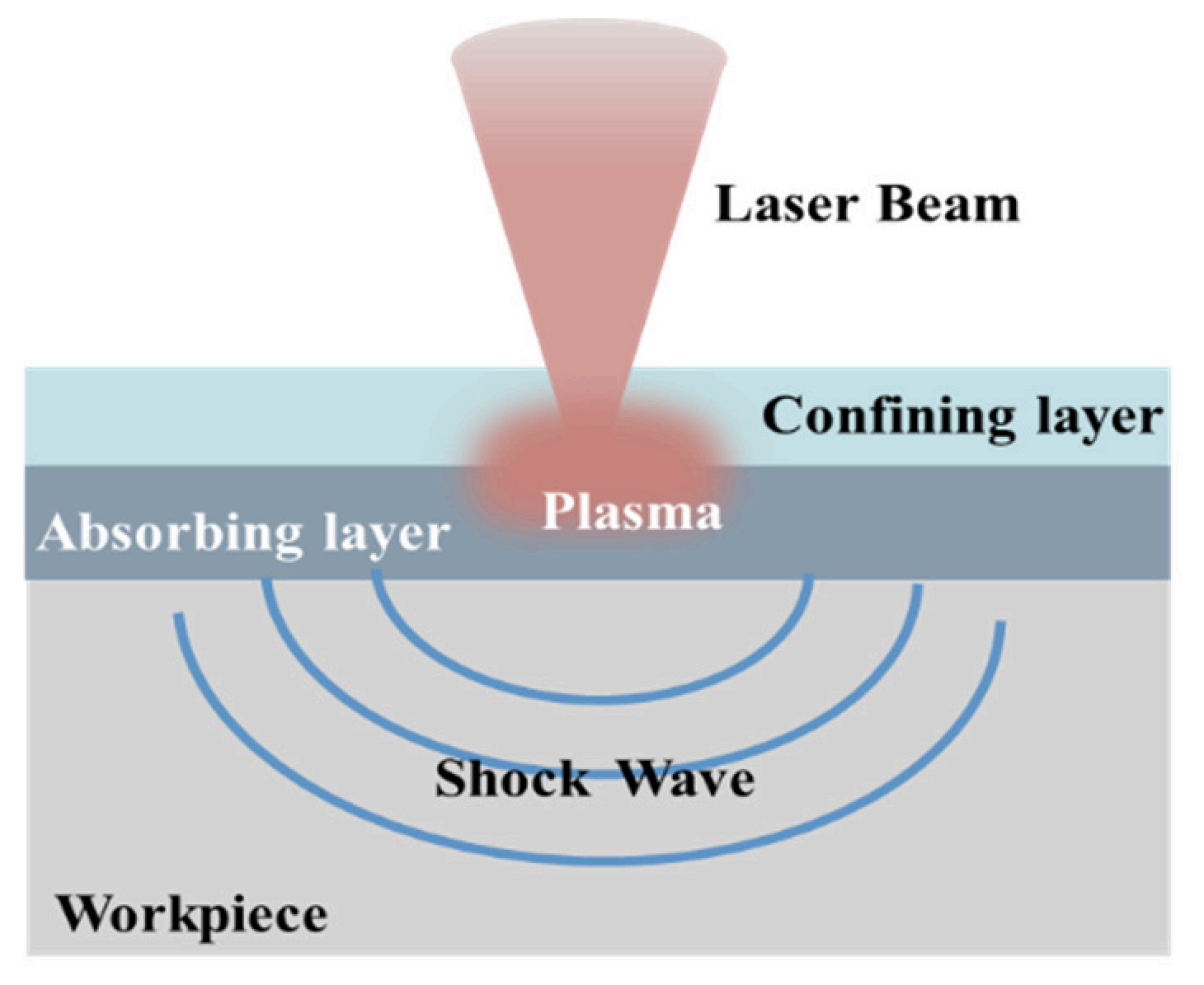
Schematic of shock-load generation during LSP. After pulsed-laser irradiation of the material surface, the absorbing layer is ablated and forms a high-temperature, high-pressure plasma. Under the constraint imposed by the transparent confinement layer, a shock load is generated and subsequently propagates into the material.

When the pulsed laser irradiates the material surface, the absorbing layer rapidly takes up the laser energy and undergoes ablation, vaporization, and ionization, producing a high-temperature, high-pressure plasma. With a transparent confinement layer placed above the surface, the free expansion of the plasma is strongly restricted, so that a larger fraction of the laser energy is converted into a shock load acting on the material surface [58]. The plasma pressure then rises sharply and is imposed on the surface as a high-pressure pulse, which subsequently develops into a stress wave propagating into the material, as shown in Figure 2 [59].

To quantitatively describe the formation of the shock load, the one-dimensional semi-empirical model proposed by Fabbro et al. is commonly used to estimate the peak shock pressure, $P_{max}$, as given in Equation (1) [60]:

(1) $$P_{max}\left(GPa\right)=0.01{\left(\frac{\xi }{2\xi +3}\right)}^{1/2}\cdot Z^{1/2}\cdot I^{1/2}$$

where ξ is the fraction of laser energy absorbed, typically taken to be approximately 0.1, and Z is the effective acoustic impedance of the confinement-layer/material system. In this model, once the laser pulse duration τ is specified, the action of the plasma pressure on the material surface can usually be represented by a finite interaction length, $L_{p}$, which can be approximated by Equation (2) [60]:

(2) $$L_{p}\left(\mu m\right)=2.10^{5}\cdot \frac{P\tau }{z}$$

where $L_{p}$ is governed not only by the pulse duration but also by the interfacial acoustic conditions of the confinement-layer/material system. The acoustic impedance of an individual medium is generally expressed as $Z_{i}=\rho_{i}D_{i}$ [61]. In the presence of a transparent confinement layer, the effective interfacial acoustic impedance is determined jointly by the acoustic properties of the confinement layer and the material, as given in Equation (3).

(3) $$\frac{2}{Z}=\frac{1}{Z_{1}}+\frac{1}{Z_{2}}$$

To further quantify the relationship between the laser parameters and power density, the laser power density is commonly calculated using Equation (4) [62].

(4) $$I=\frac{4E}{\pi D^{2}\tau }$$

where E is the single-pulse laser energy, D is the spot diameter, and τ is the laser pulse duration. Taken together, Equations (1)–(4) relate the build-up of shock loading to single-pulse energy, spot diameter, pulse duration, and interfacial acoustic conditions. These variables define the pressure input and loading scale for subsequent analyses of stress-wave propagation, plastic yielding, and residual-stress formation.

These equations therefore define a nominal pressure and power-density scale for LSP under confined-plasma, effective-impedance, and locally flat-surface assumptions [60,61,62]. In gear flanks, root fillets, tooth gaps, and bore edges, curvature, oblique incidence, and discontinuous absorbing or confinement layers may shift the actual local pressure input [36,63,64].

#### 2.1.2. Shock-Wave Propagation and Residual-Stress Development

Once the shock pressure has been established, the high-pressure load propagates into the material in the form of a stress wave. Its propagation efficiency is governed by interfacial impedance matching, reflection and transmission conditions, and the loading time history. As an engineering approximation, the peak stress within the near-surface region, $\sigma_{peak}$, can usually be related to the peak surface shock pressure, $P_{max}$, through an equivalent mapping relation, as expressed in Equation (5) [65]:

(5) $$\sigma_{peak}\approx kP_{max}\left(0<k\leq 1\right)$$

where k is the load-transfer coefficient, which accounts for interfacial impedance matching and the efficiency of load transmission. In engineering models, the peak surface shock pressure, $P_{max}$, is commonly mapped to the peak stress in the near-surface region through the coefficient k, while a piecewise time function, P(t), is used to describe the transient history of the laser-induced shock load [66]. During this process, the near-surface region first undergoes plastic deformation under a high-amplitude transient compressive load. Rapid unloading then follows, and the constraint imposed by the surrounding elastic matrix leads to the formation of a predominantly compressive residual stress field. Accordingly, the temporal evolution of the shock-pressure history can be divided into three stages: rise, decay, and termination [67].

To describe this time-dependent evolution, the laser-induced shock pressure, P(t), is often written as a piecewise function, with a typical form given in Equation (6) [68]:

(6) $$P\left(t\right)=\left\{\begin{array}{c} P\left(t\right),t<T_{r}\\ P\left(t\right){\left(\frac{t}{T_{r}}\right)}^{-4/5},T_{r}<t<T_{z}\\ P\left(t\right){\left(\frac{T_{r}}{T_{z}}\right)}^{4/5}{\left(\frac{T_{z}}{t}\right)}^{6/5},t>T_{z} \end{array}\right.$$

where $T_{r}$ denotes the characteristic time over which the shock pressure rises rapidly and is sustained, whereas $T_{z}$ represents the time at which the main shock momentum has been transferred into the material. Thus, LSP loading is a transient shock process characterized by rapid build-up, short-duration holding, and subsequent decay.

Here, the transfer coefficient and pressure-time function reduce the shock event to an equivalent loading description [65,66,68]. This is adequate for simplified analysis, but wave reflection, boundary release, and impact superposition near fillets, gaps, curved flanks, and bore edges still require geometry-aware simulation or residual-stress validation [63,69].

When the peak stress wave reaching the near-surface region equals or exceeds the dynamic yield strength of the material under high-strain-rate conditions, transient plastic deformation occurs in the surface and subsurface layers. In engineering analyses, the onset of pronounced plastic deformation is commonly assessed using the Hugoniot elastic limit (HEL), as expressed in Equation (7) [70]:

(7) $$P_{max}>HEL$$

Accordingly, the HEL can be expressed in terms of the material’s dynamic yield strength and Lamé constants, as given in Equation (8) [71]:

(8) $$HEL=\left(1+\frac{\lambda }{2\mu }\right)Y,\lambda =\frac{E\nu }{\left(1+\nu \right)\left(1-2\nu \right)},\mu =G=\frac{E}{2\left(1+\nu \right)}$$

These relations link the shock pressure to the condition required for plastic yielding. Previous studies have shown that, when the peak shock pressure is kept at approximately 2–2.5 times the material HEL, sufficiently developed and stable plastic deformation can generally be induced in the near-surface region [72]. Further increasing the shock pressure provides little additional improvement in the residual-stress level and may instead increase the risk of surface damage.

The HEL criterion is therefore a practical pressure-window guide rather than a fixed threshold for all gear steels [67,68]. In carburized, nitrided, or quenched-and-tempered gears, hardness gradients, retained austenite, martensite, carbides, and prior residual stress make the local yielding condition depth-dependent [73,74].

**Figure 3:**
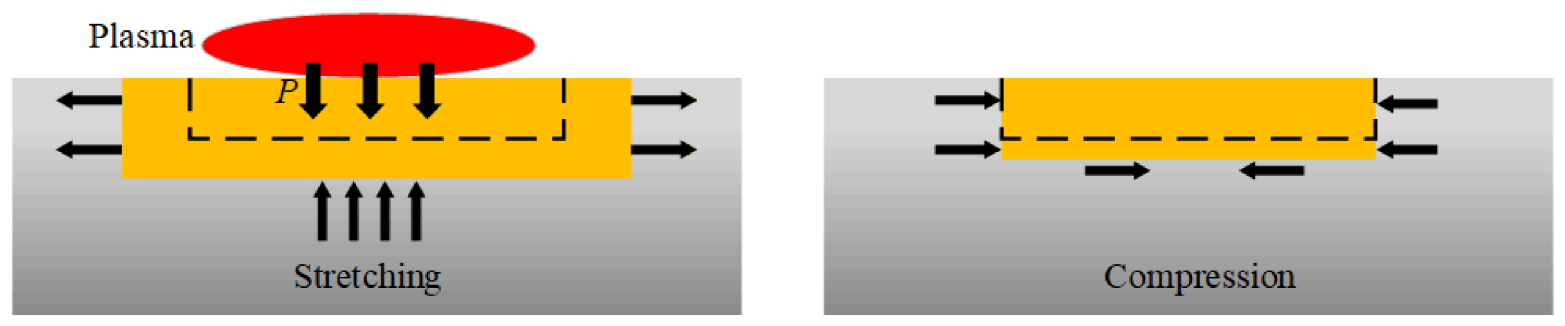
Schematic of near-surface residual-stress formation during the LSP loading–unloading process. Shock loading induces plastic deformation in the near-surface region; after unloading, CRS is generated through mechanical accommodation between the plastically deformed zone and the surrounding elastic region.

Once the plasticity condition is satisfied, the near-surface region undergoes irreversible plastic deformation followed by constrained elastic recovery. During loading, the near-surface layer is first subjected to intense normal compression. After unloading, the plastically deformed zone cannot fully recover along its original elastic path; its recovery is also constrained by the surrounding elastic matrix. As a result, a self-equilibrated residual-stress distribution dominated by compression is established in the near-surface region, as shown in Figure 3 [75].

From a quantitative standpoint, elastoplastic analytical models for circular-spot loading have related the surface residual stress to the intensity and spatial extent of the plastic deformation induced during shock loading. A representative expression is given in Equation (9) [76].

(9) $$\sigma_{gurf}=\sigma_{0}-\left(\mu \varepsilon_{p}\frac{1+\nu }{1-\nu }+\sigma_{0}\right)\left(1-\frac{4\sqrt{2}}{\pi }\frac{L}{D\sqrt{2}}\right)$$

where $\sigma_{surf}$ is the surface residual stress, $\varepsilon_{p}$ is the equivalent plastic strain (PEEQ) generated in the near-surface region during shock loading, L is the characteristic depth of the plastically deformed zone, D is the laser spot radius, μ and ν are the shear modulus and Poisson’s ratio, respectively, $\sigma_{0}$ denotes the initial stress state, and C is a coefficient related to the loading geometry. Equation (9) indicates that $\varepsilon_{p}$ governs the basic magnitude of the surface residual stress, $\sigma_{surf}$, whereas the ratio between the characteristic plastic-zone depth, L, and the spot size, D, controls the extent to which CRS is retained in the near-surface region. Hu et al. further developed an analytical framework that sequentially couples a confined-plasma expansion model with a residual-stress model. By dividing the material response into loading and relaxation stages, this framework enables prediction of the depth-dependent distribution of LSP-induced residual stress [77].

However, Equation (9) is derived under simplified circular-spot loading conditions, and its direct application to gears is restricted by scanning sequence, overlap accumulation, oblique incidence, local curvature, tooth-root constraint, tooth-gap shielding, and bore-edge release [63,64,69,78]. Thus, the analytical equations in Section 2.1 are best used for pressure-scale estimation, parameter screening, and trend interpretation, rather than direct component-level prediction.

These relations provide a mechanical basis for linking shock-pressure history, plastic yielding, constrained unloading, and CRS retention during LSP. For gear applications, however, their predictive use is bounded by assumptions on loading geometry, interfacial transfer, material homogeneity, and simplified boundary conditions. For tooth-flank contact regions and tooth-root transition regions, they are therefore more suitable for assessing whether the predicted CRS depth is compatible with the service-induced damage zone than for replacing component-level validation.

#### 2.1.3. Transparent Confinement Layer and Absorbing Layer

The LSP process relies on the coupled interfacial action of a transparent confinement layer and an absorbing layer. The transparent confinement layer suppresses the free expansion of the laser-induced plasma, thereby increasing the shock-wave pressure acting on the workpiece surface [79], while improving the conversion efficiency of laser energy into mechanical shock loading [61].

An effective transparent confinement medium must provide optical transparency, process stability, and compatibility with the component geometry. Water and glass are the most commonly used confinement materials [80]. Among them, water offers greater flexibility in arrangement and can readily conform to curved surfaces and irregular components. It also provides cooling and cleaning during processing, helping to reduce laser-induced thermal effects while removing debris and gaseous products [81].

The absorbing layer is applied to the workpiece surface and serves to absorb the incident laser energy, initiate plasma formation, and shield the substrate from thermal damage. Its ablation, vaporization, or ionization supplies the energy required to form the high-temperature, high-pressure plasma [82], while reducing adverse thermal effects such as direct laser-induced melting, ablation, and resolidification [83]. In complex local regions of gears, the continuity of the absorbing layer affects local pressure input and the stability of surface integrity. The matching between the confinement layer and the absorbing layer also defines an important boundary condition for subsequent process-window design.

### 2.2. LSP Process Parameters

**Figure 4:**
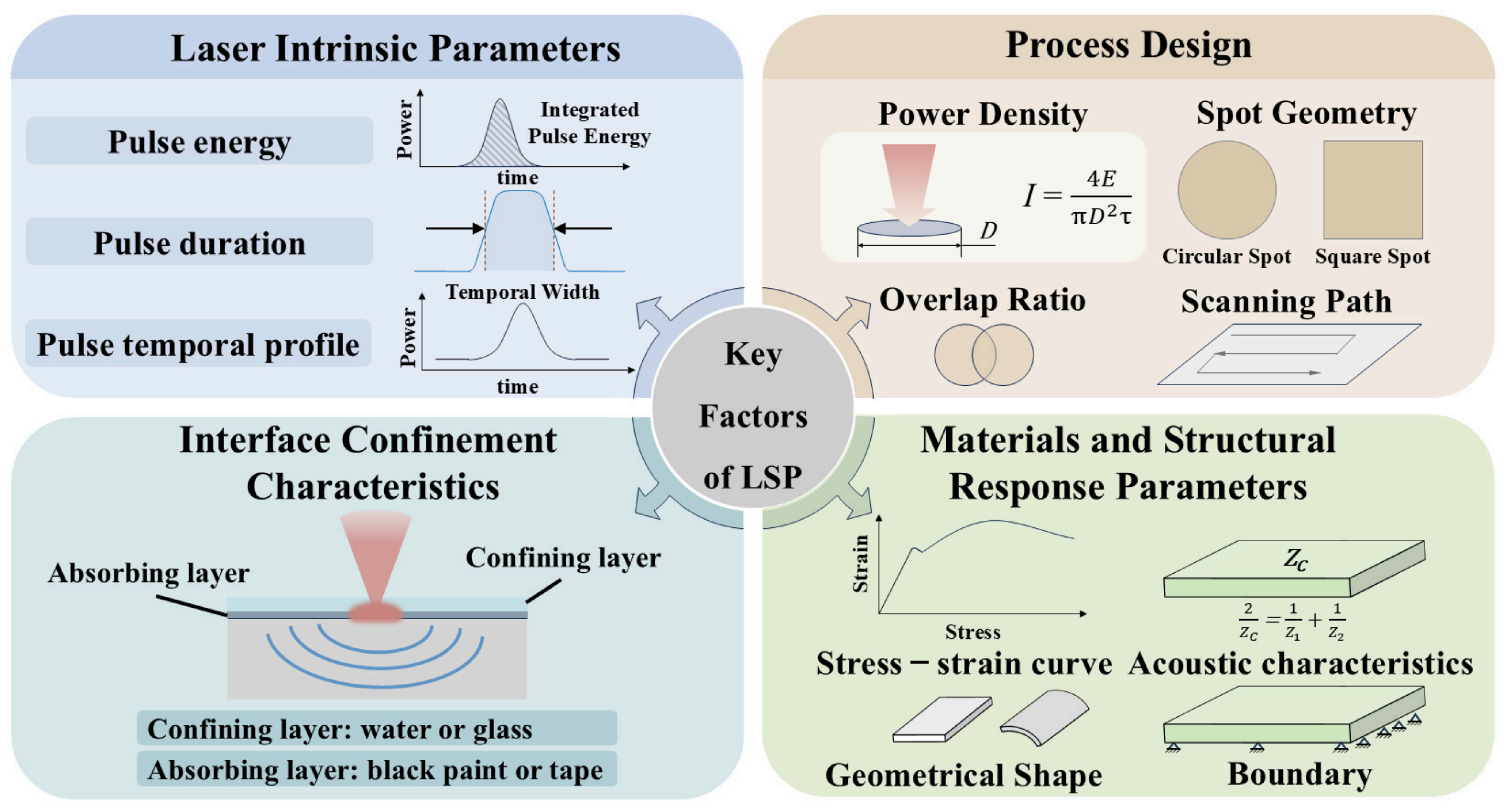
LSP process parameters.

The preceding models connect laser input and interfacial conditions with shock loading, plastic yielding, and residual-stress retention. LSP process parameters can therefore be organized into three linked groups: intrinsic laser parameters, material and structural response parameters, and processing parameters (Figure 4) [84]. Together, these groups connect pressure input, stress-wave transmission, impact superposition, and repeated loading with strengthening depth, stress-field uniformity, and surface-integrity preservation.

#### 2.2.1. Intrinsic Laser Parameters

The intrinsic laser parameters mainly include pulse energy, pulse duration, and spot size, which together determine the power density, pressure duration, and single-impact coverage scale [73]. For gear-related materials, the effective parameter window is defined by CRS depth, stress-field uniformity, surface morphology, and compatibility with local geometry.

Pulse energy and spot size regulate the shock-pressure amplitude through the power density. Increasing the power density generally enhances near-surface plastic deformation and deepens the CRS layer. Excessive energy input, however, may destabilize the residual-stress distribution, increase subsurface tensile residual stress, and promote surface damage [85,86]. Spot size also governs coverage efficiency and local adaptability. A small spot improves local access but limits the affected area. A large spot improves areal coverage, yet may cause nonuniform loading near sharp curvature transitions or component boundaries. Pulse duration controls the pressure duration and shock-momentum transfer and therefore must be matched with the energy density and spot size.

**Figure 5:**
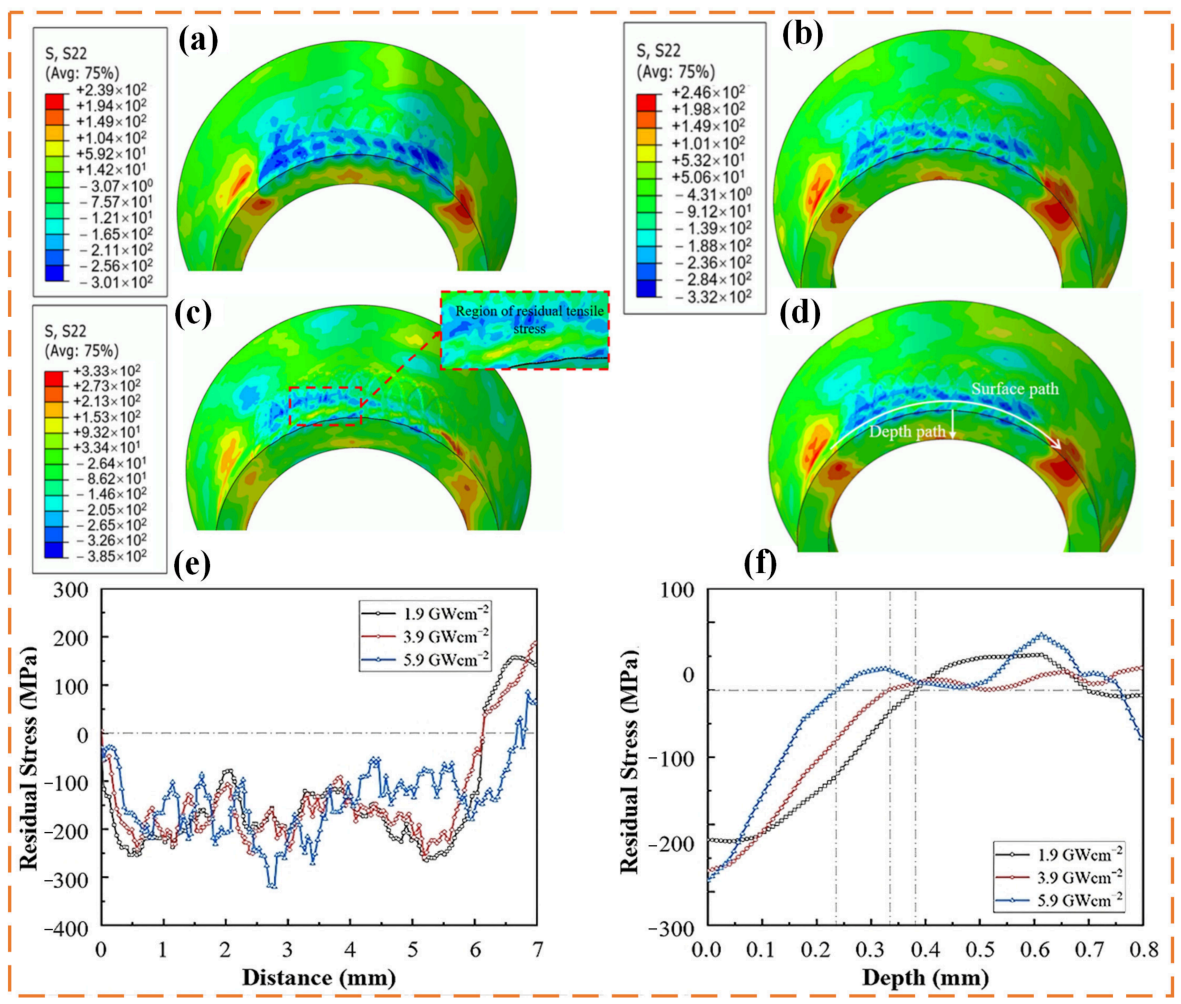
Residual-stress distributions in a stainless-steel thin-walled pipe after LSP at different laser power densities: (**a**–**c**) contours at 1.9, 3.9, and 5.9 GW cm^−2^; (**d**) extraction paths; (**e**,**f**) surface- and depth-direction profiles. Reproduced from Ref. [87] under the terms of the Creative Commons Attribution License.

The stainless-steel thin-walled pipe simulations shown in Figure 5 illustrate the finite power-density window of LSP. As the power density increased from 1.9 to 5.9 GW cm^−2^, the surface CRS became stronger, but the highest input caused stress-field nonuniformity and local tensile stress. The depth profiles further suggest that 3.9 GW cm^−2^ produced a better balance between surface compression and affected depth than 5.9 GW cm^−2^ [87]. Although this case is based on a nickel-based superalloy, it provides a useful mechanistic reference for interpreting LSP parameter effects. Its relevance to gear steels and actual gear components should be judged in relation to the heat-treatment condition, tooth geometry, and surface-integrity requirements [12,88].

Optimization of the intrinsic laser parameters therefore requires a balance among effective strengthening depth, plastic deformation intensity, stress-field uniformity, and process-induced damage.

#### 2.2.2. Material and Structural Response Parameters

In addition to the intrinsic laser parameters, the strengthening response of LSP is also governed by the dynamic response of the material and the structural conditions of the component. For the material, the elastoplastic constitutive behavior at high strain rates, dynamic yielding characteristics, and acoustic parameters such as acoustic impedance affect the coupling, propagation, and attenuation of the shock pressure within the workpiece. These factors further determine the onset and development of plastic deformation [66]. Thus, even under identical laser input conditions, different material systems may exhibit pronounced differences in the thickness of the plastically affected layer, the peak value and depth of the CRS field, and the evolution of near-surface hardening and microstructural gradients.

For real components, geometry and boundary conditions further modify the spatial distribution of the stress and plastic strain fields produced by LSP. Previous studies have shown that surface curvature can markedly alter the three-dimensional distribution of the residual stress field after LSP [63]. Variations in thickness can further affect the residual-stress field and its response during service [69], while complex three-dimensional boundaries can change the way in which the residual-stress field is spatially redistributed [89]. Such structural effects are usually more pronounced in geometrically transitional regions, including tooth-flank contact regions, tooth-root transitions, and hole edges. Parameter windows obtained from flat specimens or simple components therefore cannot be transferred directly to actual gears. Accordingly, LSP parameter design should account not only for laser input but also for the dynamic material response, local geometric features, and boundary constraints, so that the strengthening response can be matched to the service requirements of the component.

#### 2.2.3. Processing Parameters

Processing parameters determine how repeated laser impacts are arranged and accumulated on the treated surface after the intrinsic laser parameters have been selected. The scanning path controls the loading sequence [90], the spot shape determines the spatial distribution of each impact [91], the overlap ratio governs the continuity of adjacent impact zones [92], and the number of impacts controls plastic strain accumulation and repeated loading [93]. These parameters are discussed below as general processing variables affecting coverage continuity, residual-stress uniformity, strengthening saturation, and surface-integrity preservation.

(1) Scanning path

**Figure 6:**
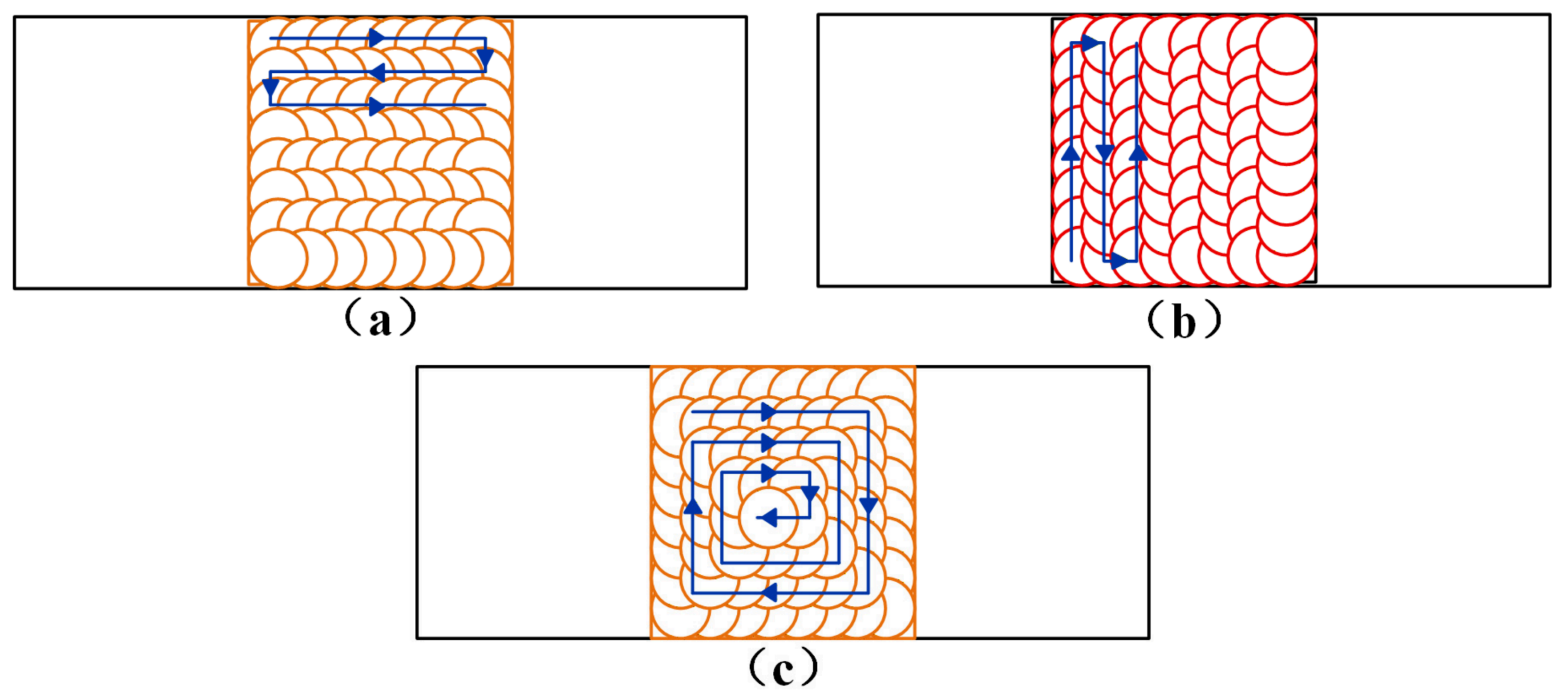
Geometry and scanning paths of specimens used for fatigue and residual-stress analysis (mm): overall specimen geometry; (**a**) X–Y scanning path; (**b**) Y–X scanning path; (**c**) L-spiral scanning path.

The scanning path defines the loading sequence and spatial arrangement of laser impact spots, and directly affects the superposition between adjacent impact zones, the accumulation path of residual stress, and stress release near boundaries [80]. Poorly designed paths may lead to missed impacts, excessive reloading, or abrupt stress gradients near boundaries, turning points, and regions with changing curvature. A well-designed path helps reduce local stress fluctuations and improves the stability of the residual-stress field [94]. The Al 2024-T351 specimens shown in Figure 6 are used here to illustrate how scanning path affects residual stress and fatigue response. Direct transfer of path rules from flat specimens to components with different curvature and boundary conditions requires caution.

(2) Spot shape

**Figure 7:**
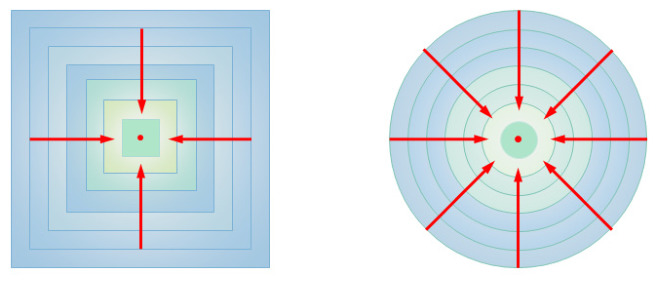
Schematic illustration of square and circular spot shapes and their corresponding pressure-wave distributions. The red arrows indicate the inward propagation and convergence of the pressure waves toward the spot center, while the concentric contours represent the corresponding spatial pressure distributions.

The spot shape defines the spatial distribution of the single-impact load on the surface. As shown in Figure 7, circular spots are widely used because they are readily compatible with conventional optical systems [73], whereas square spots provide a regular tiling pattern and can improve coverage efficiency in open processing regions [95].

Equations (1)–(4) mainly describe the peak shock pressure and its relationship with power density and acoustic impedance. When different spot shapes are compared, the spatial distribution of pressure within the spot area must also be considered. In related studies, the shock-pressure distribution under a circular spot is usually expressed in a Gaussian form. To compare the load distributions of square and circular spots under the same characteristic size, the pressure field of a square spot can be treated as an equivalent modification of the circular Gaussian pressure field. Here, 0.618 is an equivalent scaling factor obtained from the normalization relationship between spot area and pressure distribution, which makes the equivalent pressure amplitudes of the two spot types comparable [96]. When the side length of the square spot is taken to be equal to the diameter of the circular spot, the surface shock pressure can be approximated by Equation (10) [97,98]:

(10) $$P_{Squaru}\approx 0.618P_{Round}\left(r,t\right)=0.618P_{Peak}P\left(t\right)exp\left(-\frac{r^{2}}{2R^{2}}\right)$$

where $P_{peak}$ is the peak shock pressure, P(t) is the time-dependent amplitude function, r is the distance from an arbitrary point within the spot to the spot center, and R is the radius of the circular spot. Equation (10) indicates that spot shape changes the surface pressure distribution and the superposition between adjacent impact zones. The factor 0.618 is a correction coefficient introduced under specific assumptions of geometric equivalence and pressure-distribution normalization. Its applicability is therefore limited by the underlying geometric assumptions and the adopted pressure-distribution model.

Because individual impacts are discretely arranged along the scanning path, continuous strengthening relies on spatial overlap between adjacent impact spots. This overlap is required to obtain a more uniform shock-load distribution and a more consistent superposition of residual stress [99].

(3) Overlap ratio

**Figure 8:**
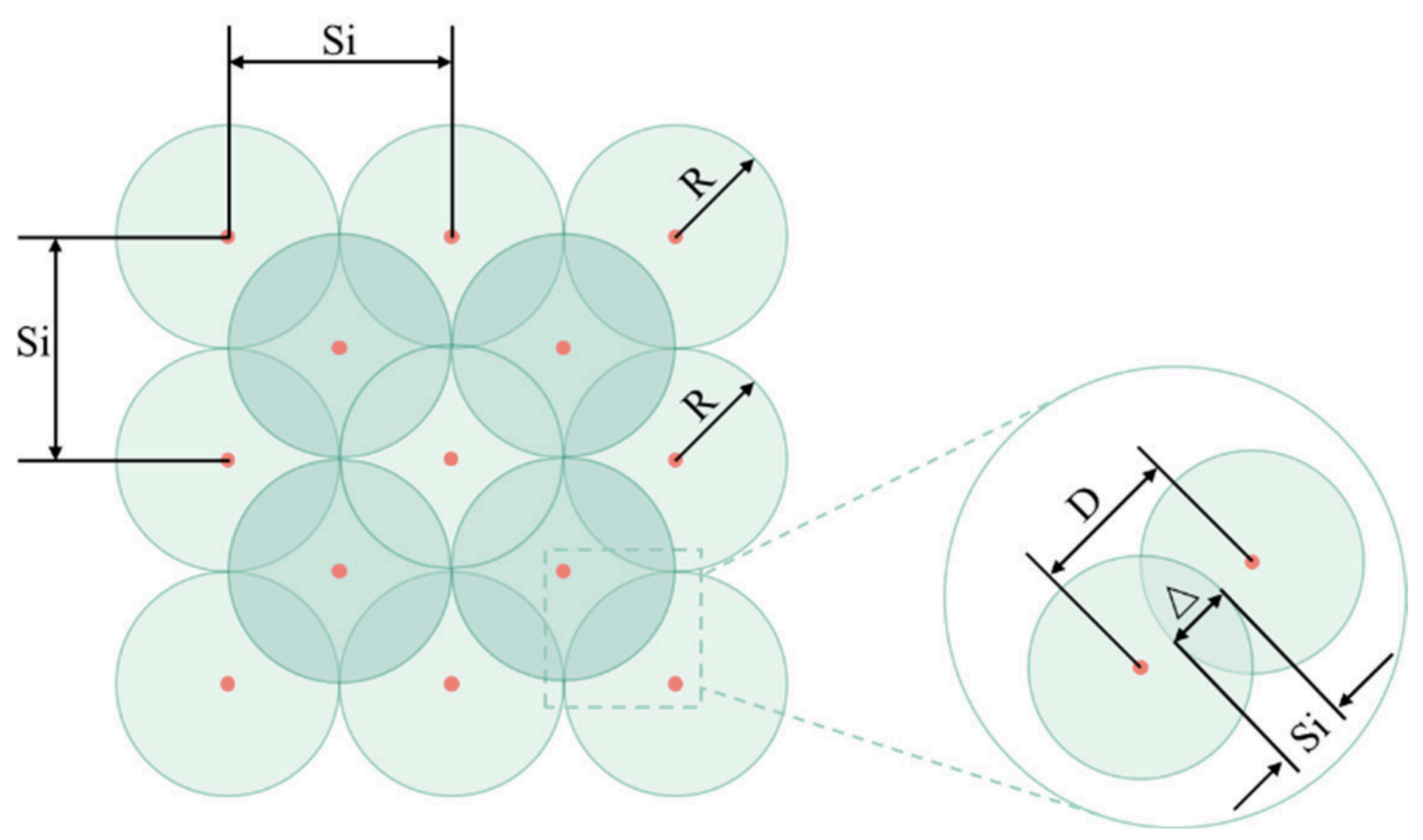
Schematic illustration of overlap ratio in LSP.

The overlap ratio describes the degree of spatial overlap between adjacent impact spots and is a key processing parameter for converting discrete impacts into a continuous strengthened region. The plastic deformation zone and residual-stress field induced by a single impact have a finite effective range and decay rapidly in the radial direction. Insufficient overlap may produce discontinuities in the residual-stress field and leave unprocessed areas within the strengthened region. Excessive overlap, by contrast, increases local repeated loading, drives the residual-stress benefit toward saturation, and may promote surface roughening, micropitting, and disturbances in the local surface profile. The overlap-ratio window should therefore be defined by the continuity of residual stress, the cost to surface integrity, and the geometric constraints of the component [56]. Under regular scanning conditions, the overlap ratio can be expressed as Equation (11) [84]:

(11) $$\eta =1-\frac{s}{D},0<s\leq D$$

where s is the center-to-center spacing between adjacent impact spots, and D is the characteristic spot size (Figure 8). When s=D, η=0, corresponding to no overlap; when s→0, η→1, corresponding to highly repeated loading. Equation (11) gives the geometric definition of the overlap ratio.

(4) Number of impacts

The number of impacts affects near-surface plastic strain accumulation, residual-stress superposition, and surface integrity. An appropriate increase in impact number can raise both the CRS level and the degree of hardening, but the benefit weakens once the plastic response approaches saturation. Shu et al. showed that, for 30CrMnSiNi2A high-strength steel, additional impacts were more effective in improving fatigue life than simply increasing the energy density. In terms of CRS, the difference between one impact at 7.89 GW·cm^−2^ and three impacts at 6.57 GW·cm^−2^ was no longer pronounced, indicating saturation of the residual-stress gain under repeated impacts [47]. In additively manufactured TA15 alloy, the fatigue lives after one and two impacts reached 2.34 and 2.56 times that of the as-built state, respectively, showing that the incremental benefit of the second impact had already weakened substantially [100]. These results indicate that impact number should be treated as a coupled processing variable together with energy density, spot size, scanning path, and overlap ratio. Repeated loading improves plastic strain accumulation and CRS retention only within a finite strengthening window; beyond this range, additional impacts mainly increase the risk of surface roughening, micropitting, and local over-peening [101].

### 2.3. Parameter Considerations for Gear Applications

The general processing parameters discussed above require additional constraints when LSP is applied to gear components. Compared with flat specimens, gears contain curved contact surfaces, tooth-root fillets, tooth gaps, bore edges, and local boundaries that alter beam accessibility, incidence angle, spot superposition, confinement-layer retention, and residual-stress continuity [64,78]. Parameter selection should therefore account not only for nominal power density, spot size, overlap ratio, and impact number, but also for local geometry, coverage continuity, surface-profile preservation, and the target depth of the strengthened layer. The useful range is judged from the layer that remains after peening and finishing. It is inadequate when CRS depth, hardened support, or coverage fails to reach the bending/contact-fatigue zone; with higher inputs, roughness, tensile pockets, ablation, profile drift, or finishing removal can outweigh further CRS or hardness gains.

Component-level evidence remains limited. Pathak et al. showed that LSPwC of selective laser melting(SLM) mesoscale gears converted tensile residual stress in tooth-root and tooth-gap regions into CRS and improved surface quality and near-surface microstructure [102]. For laser powder bed fusion(LPBF) 17-4 PH micro-spur gears, LSP increased the effective CRS depth to approximately 0.4 mm, reduced surface roughness by about 32%, and caused only minor changes in gear microgeometry [103]. The available component-level evidence shows that LSP can regulate surface and subsurface states around tooth-root, tooth-gap, and tooth-profile features, but the evidence is still concentrated on additively manufactured micro- and mesoscale gears. Further validation remains necessary for conventional carburized or nitrided gear steels, large components, and heavy-load service conditions.

For industrial gear manufacturing, LSP parameter selection should be treated as a geometrically constrained optimization problem rather than a set of single-factor empirical trials. Li et al. provide a useful model-based example: maximum residual stress, residual-stress depth, surface plastic strain, and a roughness factor were optimized through weights and constraints, bringing stress gain and surface degradation into the same parameter search [104]. For gears, this logic must be further combined with profile tolerance and post-finishing retention. Finite element method (FEM) remains necessary for pre-screening pulse energy, spot size, overlap, scanning path, and incidence angle, because the stress-wave boundary conditions and surface-deformation responses vary among tooth flanks, root fillets, tooth gaps, and bore walls. To reduce the cost of repeated simulations, Li et al. combined validated FEM data with ensemble learning for rapid residual-stress prediction [105]. Residual-stress holes in overlap regions further more make parameter selection a spatial-control problem; Zhou et al. used random forest regression to optimize peak power, spot diameter, number of impacts, and overlap ratio for this purpose [106]. Zhao et al. extended the prediction target from residual stress to microhardness by introducing shock-wave pressure, attenuation, material parameters, and impact number into an artificial neural network (ANN)–physical model [107]. In production, however, the selected window still requires signal-level verification. Interpretable acoustic-emission networks provide one route for linking transient shock signals with stress-state monitoring [108]. A gear-LSP digital twin should therefore remain a future framework connecting geometry, Finite element method/machine learning (FEM/ML) prediction, process signals, and inspection data, with validation and data synchronization still unresolved [109].

## 3. LSP-Induced Near-Surface State Evolution and Strengthening Response

LSP converts transient shock loading into retained near-surface states, including CRS layers, plastic strain accumulation, hardness gradients, and dislocation-mediated microstructural refinement [41,110]. For gear steels, these changes are relevant only when their depth, magnitude, and spatial uniformity correspond to damage-prone regions near the tooth flank and tooth root. Because component-level studies of LSP-treated gears remain relatively limited, the analysis is based mainly on gear steels and related load-bearing steels, while selected results from non-gear metallic materials are used only to clarify general features of LSP-induced near-surface state evolution. The key issues are whether the CRS layer can cover damage-prone regions, whether plastic strain accumulation and hardness gradients can provide stable near-surface load-bearing support, and whether microstructural refinement and defect reconfiguration can explain the microscopic origin of these strengthening responses.

### 3.1. LSP-Induced Plastic Strain Accumulation and CRS Evolution

The CRS layer is one of the most widely examined near-surface responses after LSP treatment. For gear-related materials, the CRS response can be evaluated in terms of compressive-stress magnitude, affected depth, and distribution state. These metrics help determine its correspondence with damage-prone regions such as the tooth-flank contact region and the tooth-root transition region, and provide the stress-state basis for subsequent analyses of fatigue, wear, and crack-initiation behavior.

#### 3.1.1. Formation, Depth Profile, and Uniformity of the CRS Layer

In steels and steel-alloy gear-related materials, LSP introduces constrained plastic deformation from the surface into the subsurface region through shock-wave loading. After unloading, the mismatch between the plastically deformed zone and the surrounding elastic matrix leaves a residual-stress layer dominated by compression. The depth profile of residual stress reflects shock-wave attenuation, plastic deformation retained after unloading, and near-surface stress redistribution, and is therefore widely used to characterize the strengthened near-surface state after LSP [111]. For gear damage-prone regions, the surface compressive stress describes the local stress magnitude, whereas the peak position, affected depth, and spatial uniformity more directly determine whether the compressive stress field is properly matched to the critical damage layer.

**Figure 9:**
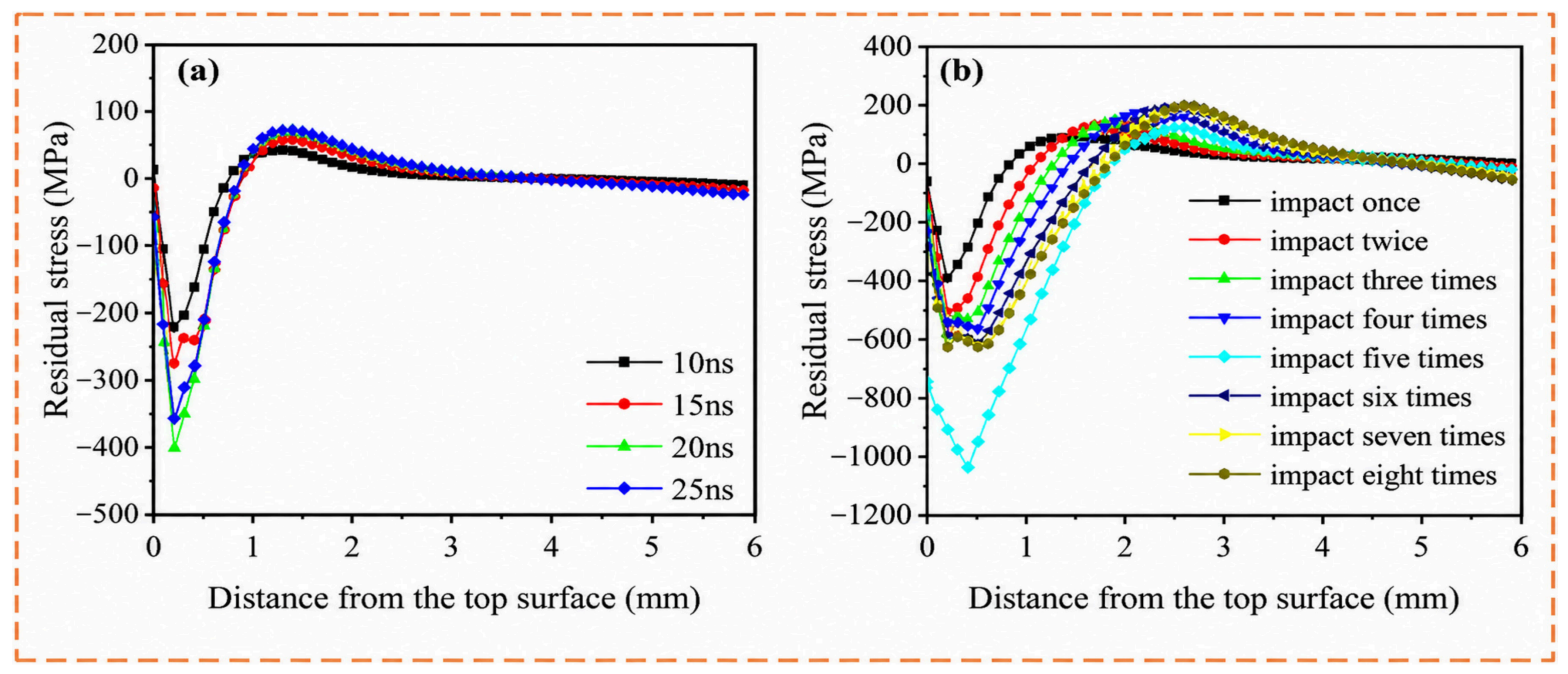
Depth-direction CRS profiles of LSP-treated 20CrMnTi carburized steel: (**a**) effect of pulse energy on residual-stress distribution; (**b**) effect of impact number on residual-stress distribution. Panels (**a**,**b**) are reproduced from Ref. [112] under the terms of the Creative Commons Attribution License.

For 20CrMnTi carburized steel, FEM calculations checked against X-ray residual-stress measurements reveal strong parameter sensitivity of the CRS profile. As shown in Figure 9a, increasing the pulse energy from 4 to 7 J deepened and intensified the compressive field until the response saturated near 5 J; beyond this range, the 7 J condition produced a local tensile-stress pocket at the impact center, showing that excessively high energy can weaken the integrity of the CRS field. The peak CRS was located approximately 200 μm below the surface, and the compressive layer extended to about 0.9 mm. Pulse width and impact number further modified the in-depth CRS response. The highest depth-direction CRS was obtained at a pulse width of 20 ns, whereas five impacts gave the strongest single-point response, with a CRS-affected depth greater than 1 mm and a maximum CRS of approximately 1085 MPa, as shown in Figure 9b [112]. A study on LSP uniformity in 20CrNiMo steel further showed that overlap ratio, pulse energy, pulse duration, and spot diameter jointly affect the residual stress and plastic strain distributions, with overlap ratio having the strongest effect on uniformity. Under an overlap ratio of 62.5%, a pulse energy of 6 J, a pulse duration of 20 ns, and a spot diameter of 3 mm, the bending fatigue life increased to 5.41 times that of the unpeened specimen [113]. These results converge in showing that fatigue improvement requires the LSP-induced CRS field to reach the damage-prone layer and remain continuous across the treated region.

#### 3.1.2. CRS Response and Surface-State Boundaries Within the Process Window

**Figure 10:**
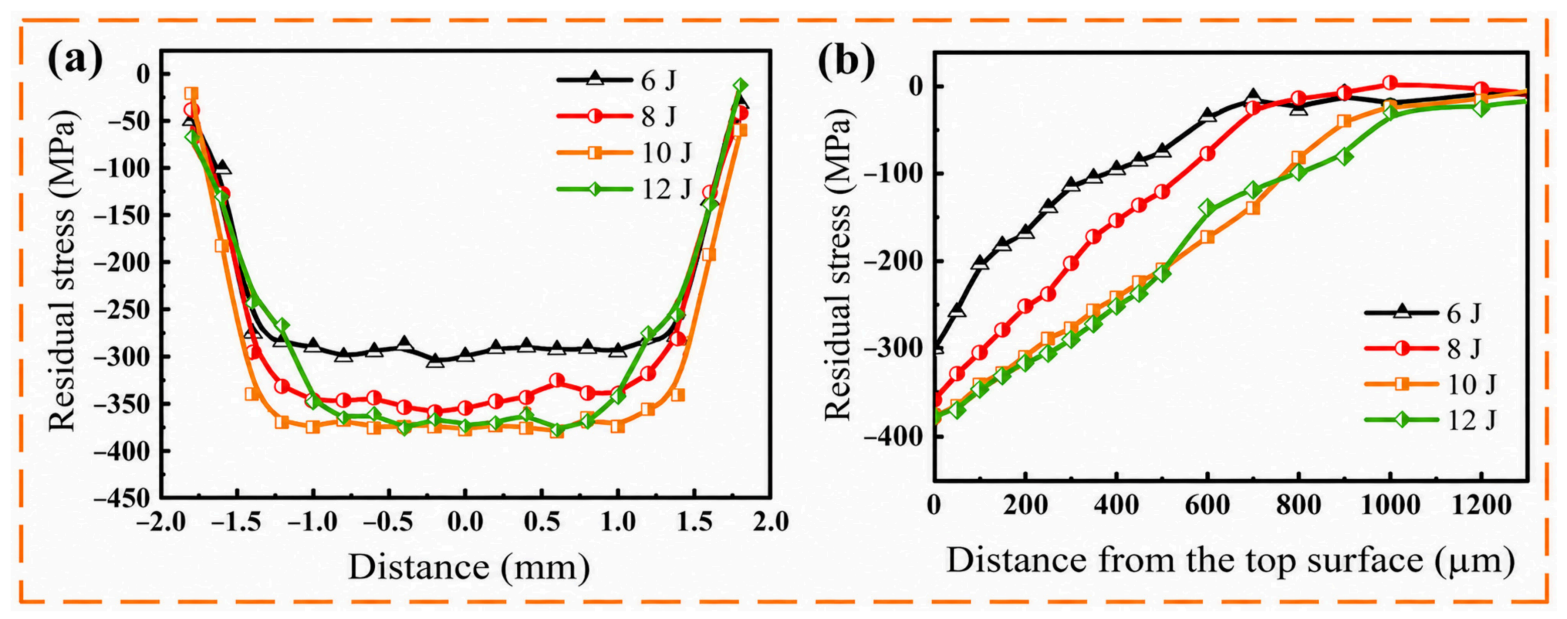
Residual-stress distributions in 40Cr steel and 316L stainless steel after LSP. For 40Cr steel: (**a**) surface residual-stress distributions under different pulse energies; (**b**) residual-stress depth profiles under different pulse energies. Panels (**a**,**b**) are reproduced from Ref. [114] under the terms of the Creative Commons Attribution license.

Process parameters affect the CRS response mainly through the coupled adjustment of residual-stress magnitude, affected depth, plastic deformation range, and surface state. Studies on 40Cr steel provide evidence for an energy window in LSP. Theoretical calculations suggested an appropriate shock-wave peak pressure range of 5.09–6.36 GPa, and simulations identified 20 ns as the preferred pulse duration. Under a pulse duration of 20 ns and a spot diameter of 3 mm, increasing the pulse energy from 6 J to 10 J enhanced both the surface CRS and the maximum CRS along the depth direction. As shown in Figure 10a,b, the maximum CRS values at 6, 8, 10, and 12 J were −300, −359, −378, and −375 MPa, respectively, while the corresponding residual-stress-affected depths were 690, 810, 1050, and 1058 μm. When the energy was further increased from 10 J to 12 J, the CRS peak decreased slightly and the increase in affected depth nearly ceased, indicating that higher energy input did not produce further CRS improvement. This behavior is associated with residual-stress relaxation caused by surface rarefaction waves and reverse plastic deformation [114]. CRS saturation can be judged from depth-resolved stress profiles obtained across an increasing parameter series. In 40Cr steel, the 10–12 J range shows almost no further increase in peak compression or compressive affected depth, indicating a shift from useful CRS gain to stress relaxation and redistribution [114]. LSPwC studies show the same tendency: further input weakens CRS improvement while surface roughening becomes more severe [115]. In H13 steel, repeated impacts still extend the compressive layer, but a residual-stress hole develops at the spot center, where CRS and plastic strain become lower than those on both sides [116]. Such profile plateaus, stress holes, and roughening define the upper boundary of the useful LSP process window in gear-related applications because additional input may weaken stress-field continuity and surface integrity.

### 3.2. Dislocation-Dominated Substructure Refinement and Gradient Microstructural Evolution

The microstructural gradient produced by LSP arises from the nonuniform accumulation of high-strain-rate plastic deformation in the near-surface region. In gear steels and gear-related high-strength steels, grain-orientation dispersion, increased kernel average misorientation (KAM), dislocation tangles, and substructural subdivision are typically concentrated within the LSP-affected layer and gradually diminish with increasing depth. This depth-dependent gradient corresponds closely to the formation ranges of the CRS layer and the hardened layer.

**Figure 11:**
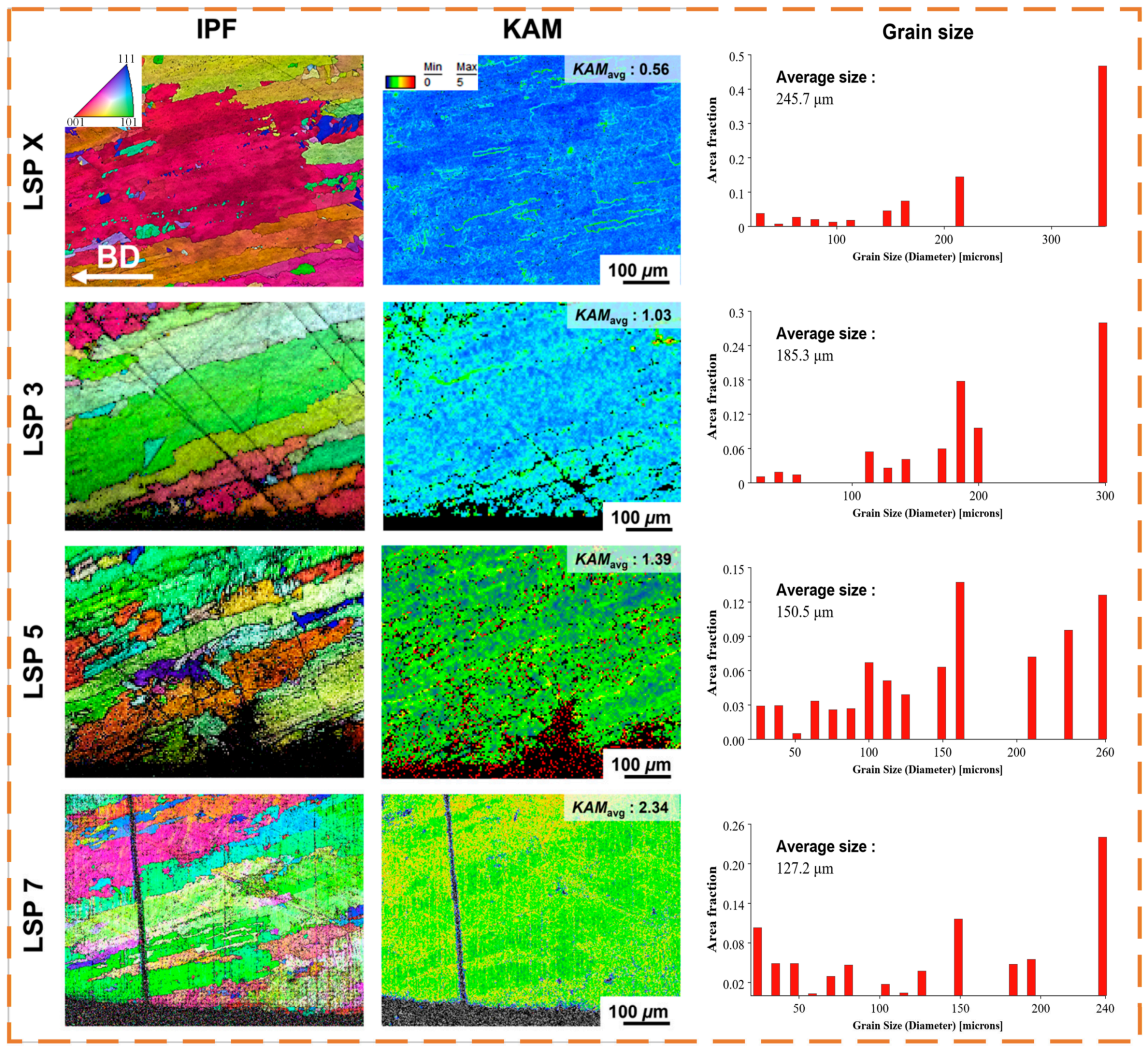
EBSD characterization of L-DED 316L stainless steel after different LSP passes: IPF maps, KAM maps, and average grain size measurements for unpeened and LSP-treated specimens. Panels are reproduced from Ref. [117] under the terms of the Creative Commons Attribution License.

Electron backscatter diffraction (EBSD)maps of LSP-treated laser directed energy deposition (L-DED) 316L stainless steel captured the near-surface refinement process in direct crystallographic terms. The inverse pole figure (IPF) maps changed from coarse columnar grains to a finer, more fragmented structure after repeated LSP, and seven passes reduced the average grain size from approximately 245.7 to 127.2 μm. In the KAM maps, KAM_avg increased from about 0.56 to 2.34, consistent with stronger local orientation gradients and a higher density of deformation-induced dislocations within the shocked layer (Figure 11) [117].

**Figure 12:**
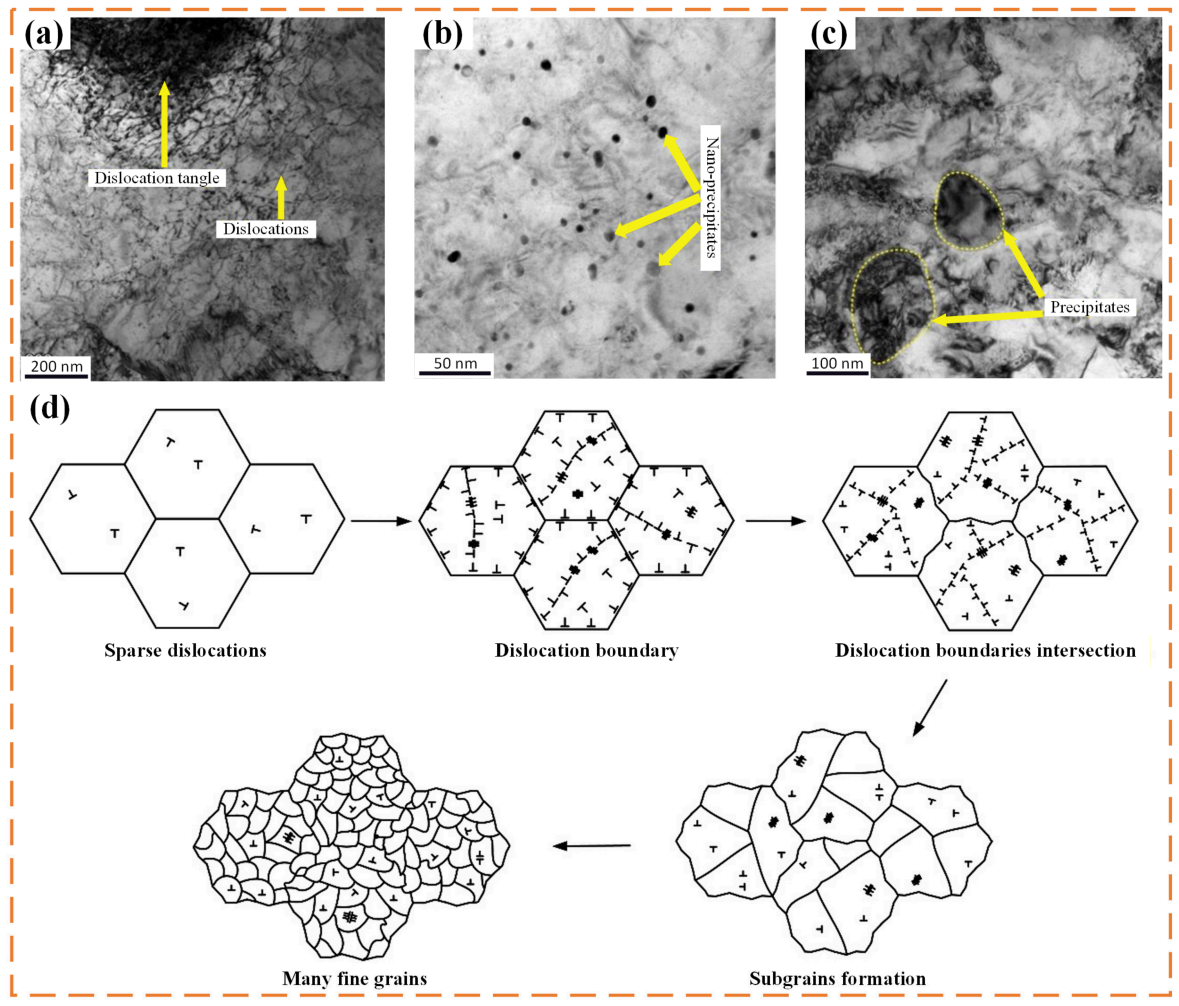
Dislocation configurations and refinement mechanism in an LSP-treated NV E690 high-strength steel cladding layer: TEM observations after LSP at (**a**) 4.77, (**b**) 7.96, and (**c**) 11.15 GW cm^−2^; (**d**) dislocation-mediated grain-refinement model. In panel (**d**), the T-shaped and other short-line symbols schematically represent dislocations, while their increasing density and rearrangement illustrate the formation of dislocation boundaries, subgrains, and fine grains.Panels are adapted from Ref. [118] under the terms of the Creative Commons Attribution License.

TEM observations of an LSP-treated NV E690 high-strength steel cladding layer trace the defect evolution produced by shock loading. At 4.77 GW cm^−2^, dense dislocation tangles formed near grain boundaries, pointing to rapid multiplication of mobile dislocations in the surface layer. Raising the power density to 7.96 GW cm^−2^ introduced nanoscale precipitates into a more defect-rich matrix, where particle obstruction and dislocation accumulation intensified local defect interactions. At 11.15 GW cm^−2^, dislocations with different orientations intersected more extensively, and locally refined regions began to appear. The model in Figure 12d condenses this sequence into dislocation accumulation, boundary formation and intersection, subgrain subdivision, and final refinement into fine grains [118]. With continued plastic strain accumulation, the dense dislocation network underwent further entanglement and rearrangement, subdividing the original grains into smaller substructural units and increasing the grain-boundary density. Similar dislocation rearrangement, subgrain-boundary formation, and substructural evolution have also been observed in severely plastically deformed 304L stainless steel [74]. The increased KAM reflects local orientation gradients and the accumulation of geometrically necessary dislocations, whereas the dislocation cells and walls observed by TEM indicate that these defects contribute to substructural subdivision after rearrangement. Together with the depth-dependent attenuation of microstructural refinement, these observations suggest that the gradient microstructure produced by LSP arises mainly from the coupled effects of shock-wave attenuation, nonuniform plastic deformation, and dislocation-structure rearrangement. Near the surface, this response is manifested as grain refinement; at greater depths, it is expressed as the nonuniform evolution of dislocation structures along the depth direction.

Further local plastic strain accumulation may drive substructural refinement toward a nanocrystalline structure. TEM and SAED analyses of the surface layer of E690 high-strength steel revealed nanocrystalline features, with near-surface grain sizes of approximately 80–200 nm [119]. This observation suggests that dislocation accumulation and substructural subdivision can continue to promote grain refinement in locally high-strain regions. The gradient microstructure formed through dislocation rearrangement and substructural subdivision provides the defect-structural basis for hardened-layer formation and CRS retention. Its contribution to damage evolution in tooth-flank contact regions and tooth-root transition regions still needs to be assessed in connection with fatigue, wear, and crack behavior.

### 3.3. Summary

LSP mainly strengthens gear-related steels by leaving a near-surface compressive stress field, together with local hardening and dislocation rearrangement. Its benefit depends less on the peak stress alone than on whether this strengthened layer reaches the tooth-flank or tooth-root damage zone without degrading surface integrity. Excessive peening may instead cause roughening, stress nonuniformity, or local damage. This depth–integrity balance still needs gear-level validation.

## 4. Effects of LSP on Surface Integrity and Failure Behavior

Near-surface strengthening in gears becomes technically meaningful when the retained CRS layer, hardened layer, plastically deformed zone, and gradient microstructure improve surface integrity and resistance to service-related damage. These near-surface states should therefore be evaluated together with surface morphology, defect distribution, and stability under cyclic loading, because crack initiation, fatigue response, and wear-assisted damage are controlled by their coupled evolution. Given the limited component-level evidence for LSP-treated gears, the analysis gives priority to bending fatigue, rolling contact fatigue (RCF), and wear results obtained from gear components, gear steels, and related load-bearing steels. Results from non-gear metallic materials are used mainly to clarify the mechanistic links between surface-integrity attributes and damage evolution.

### 4.1. Surface Integrity Characterization and Its Influence on Gear Failure Behavior

The surface integrity of steels and steel-alloy gears can be assessed in terms of surface morphology, CRS, hardness gradients, microstructural gradients, and defect state [120,121,122]. Surface morphology affects asperity contact, lubricant-film retention, and the susceptibility of the surface layer to microcracking. The peak value, affected depth, continuity, and cyclic relaxation of CRS modify crack-tip opening tendencies and the subsurface stress field. Hardness and microstructural gradients reflect the near-surface load-bearing support, plastic strain accumulation, and cyclic stability [123].

From the perspective of gear failure mechanisms, tooth-flank contact regions and tooth-root transition regions show different sensitivities to individual surface-integrity attributes. Tooth-flank contact regions are more strongly affected by surface morphology, wear grooves, local defects, and the position of the subsurface shear-stress maximum. Tooth-root transition regions depend more on CRS depth, compressive-stress continuity, and resistance to crack growth [124]. Accordingly, evidence from gear steels, gear-related steels, single-tooth bending tests, and rolling contact fatigue (RCF)RCF studies should form the primary basis for assessment. Results from high-strength steels, stainless steels, and selected non-steel materials are used as supporting evidence to clarify material responses such as roughness evolution, hardened-layer retention, crack-growth retardation, defect tolerance, and wear mechanisms.

### 4.2. LSP-Induced Modification of Surface Integrity

The influence of LSP on surface integrity is mainly reflected in three types of responses: surface morphology reconstruction, gradient hardening, and changes in defect state [125]. Within a stable process window, impact-induced plastic flow can blunt some sharp asperities and generate a near-surface hardening gradient through dislocation multiplication and rearrangement [126]. When the process parameters or interfacial conditions become unstable, however, ablation pits, surface roughening, and local stress nonuniformity may act as new crack-sensitive sites [127]. For gear steels and related materials, these responses primarily affect asperity contact, near-surface load-bearing support, and early damage initiation. Corrosion and passivation behavior is more relevant under humid–thermal, salt-spray, or lubrication-disturbed environments, where surface damage may be further amplified.

#### 4.2.1. Surface Roughness and Local Topographical Response

In tooth-flank contact, surface topography governs how asperities enter contact, how lubricant is stored or expelled, and where near-surface cracks are likely to initiate. After LSP, $R_{a}$ or $S_{a}$ cannot fully characterize this state. Impact-pit depth, rim extrusion, local surface pile-ups, peak-height distribution, and texture directionality define the real bearing condition of the treated surface. When crater-like textures or periodic height variations develop across peening tracks, these local features may produce uneven contact, local stress concentration, and preferential crack-initiation sites.

**Figure 13:**
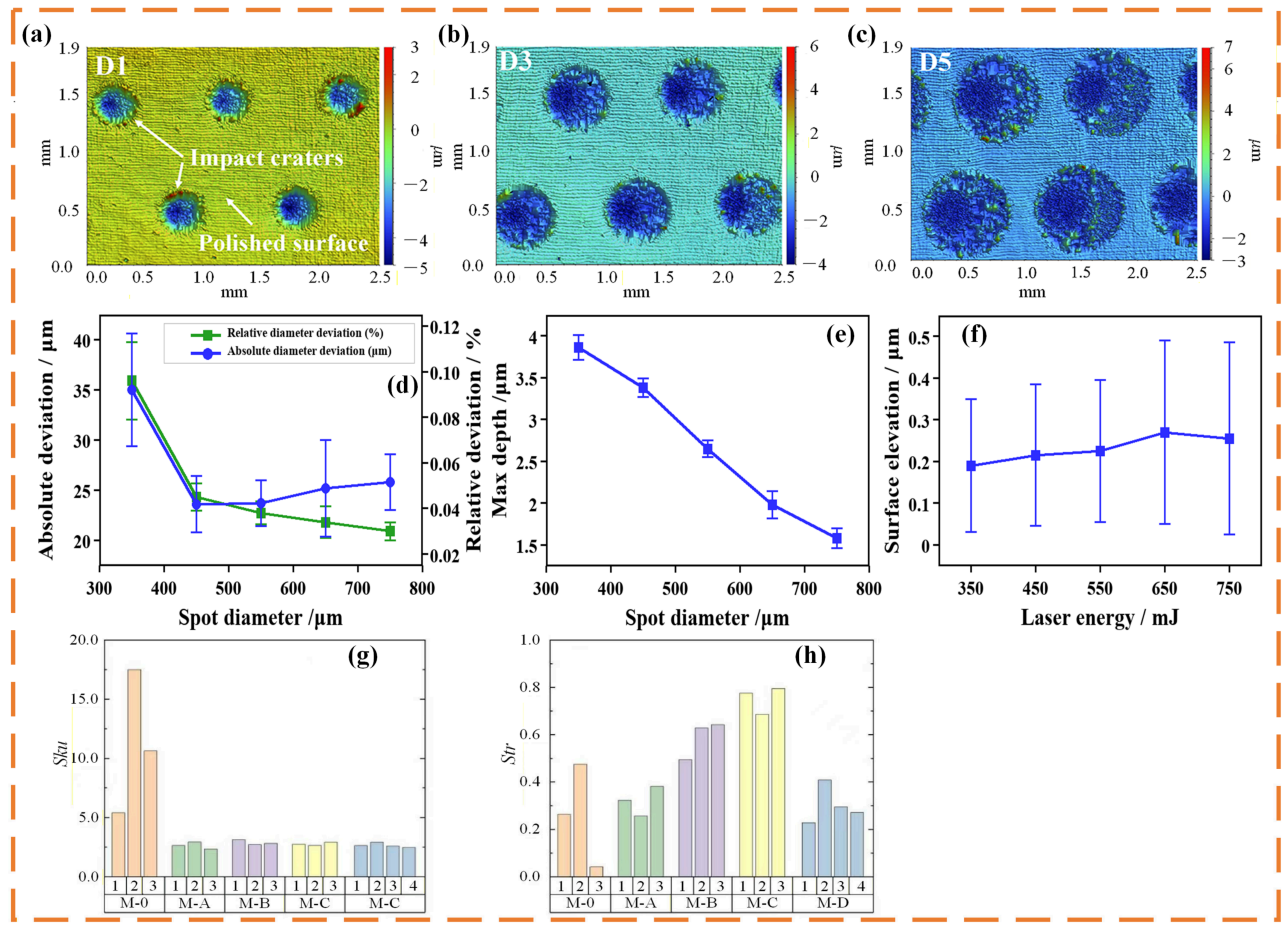
Local topography after LSP and LSPwC treatment: (**a**–**c**) representative three-dimensional crater textures of LSPwC-treated AISI 9310 steel under D1, D3, and D5 spot-diameter conditions; (**d**–**f**) diameter deviation, maximum crater depth, and surface roughness as functions of spot diameter; (**g**,**h**) *S_ku_* and *S_tr_* of LSP-treated 20CrMnTi steel after overlapped impacts. Panels (**a**–**f**) are adapted from Ref. [128], and panels (**g**,**h**) are adapted from Ref. [129], under the terms of the Creative Commons Attribution License.

LSPwC texturing of carburized AISI 9310 steel and overlapped LSP of 20CrMnTi steel illustrate how LSP reshapes surface topography beyond changes in average roughness. In the AISI 9310 case, D1, D3, and D5 correspond to increasing spot diameters from 350 to 750 μm at the same pulse energy; the resulting decrease in energy density changed the crater morphology from compact, sharply defined impact pits to larger textures with stronger rim extrusion and local surface pile-ups (Figure 13a–c). The diameter deviation and maximum crater depth decreased as the spot diameter increased, although micrometer-scale relief persisted around the impact region and reached its highest level near 650 μm (Figure 13d–f) [128]. In the 20CrMnTi study, overlapped LSP altered the peak–valley population rather than simply increasing the areal height parameters. S_ku_ decreased from 11.18 before LSP to about 2.54–2.89 after treatment, indicating a flatter and broader height distribution; S_tr_ varied with incident angle and impact arrangement, reflecting changes in surface periodicity and texture directionality (Figure 13g,h) [129]. In tooth-flank contact, these local features carry direct mechanical significance: crater depth and rim extrusion affect contact conformity, peak-height redistribution changes asperity loading, and texture directionality influences lubricant retention and early microcrack susceptibility.

Results for austempered ductile iron (ADI) and 2024-T351 aluminum alloy further indicate that high overlap ratios, absorbing-layer failure, or unstable processing conditions can aggravate ablation pits, material removal, and surface roughening [34,130].

Absorbing/confinement stability and impact scale define the surface-integrity side of the LSP window through crater depth and peak–valley features. Higher energy, overlap, or impact number may deepen the compressive zone, but excessive input can induce stress relaxation, tensile pockets, or residual-stress holes [114,115,116], together with crater relief, rim extrusion, ablation pits, material removal, and unstable height distributions [128,129,130]. In tooth-flank contact, these features affect asperity loading, lubricant-film continuity, and micropitting sensitivity [131]. For gear-related steels, $R_{a}$ and $S_{a}$ should be considered together with local pit/dimple geometry, profile retention after peening or finishing, and retained CRS depth when judging whether deeper compression remains mechanically useful [103,131,132].

#### 4.2.2. Microhardness Gradient and Near-Surface Load-Bearing Support

After LSP, gear steels and related load-bearing steels usually develop a hardness gradient that transitions gradually from a hardened surface layer to the substrate. Increased surface hardness improves the initial resistance to indentation, while a deeper hardened zone expands the load-bearing region. A smooth hardness gradient can also suppress local plastic strain accumulation and reduce crack-initiation susceptibility under cyclic contact.

**Figure 14:**
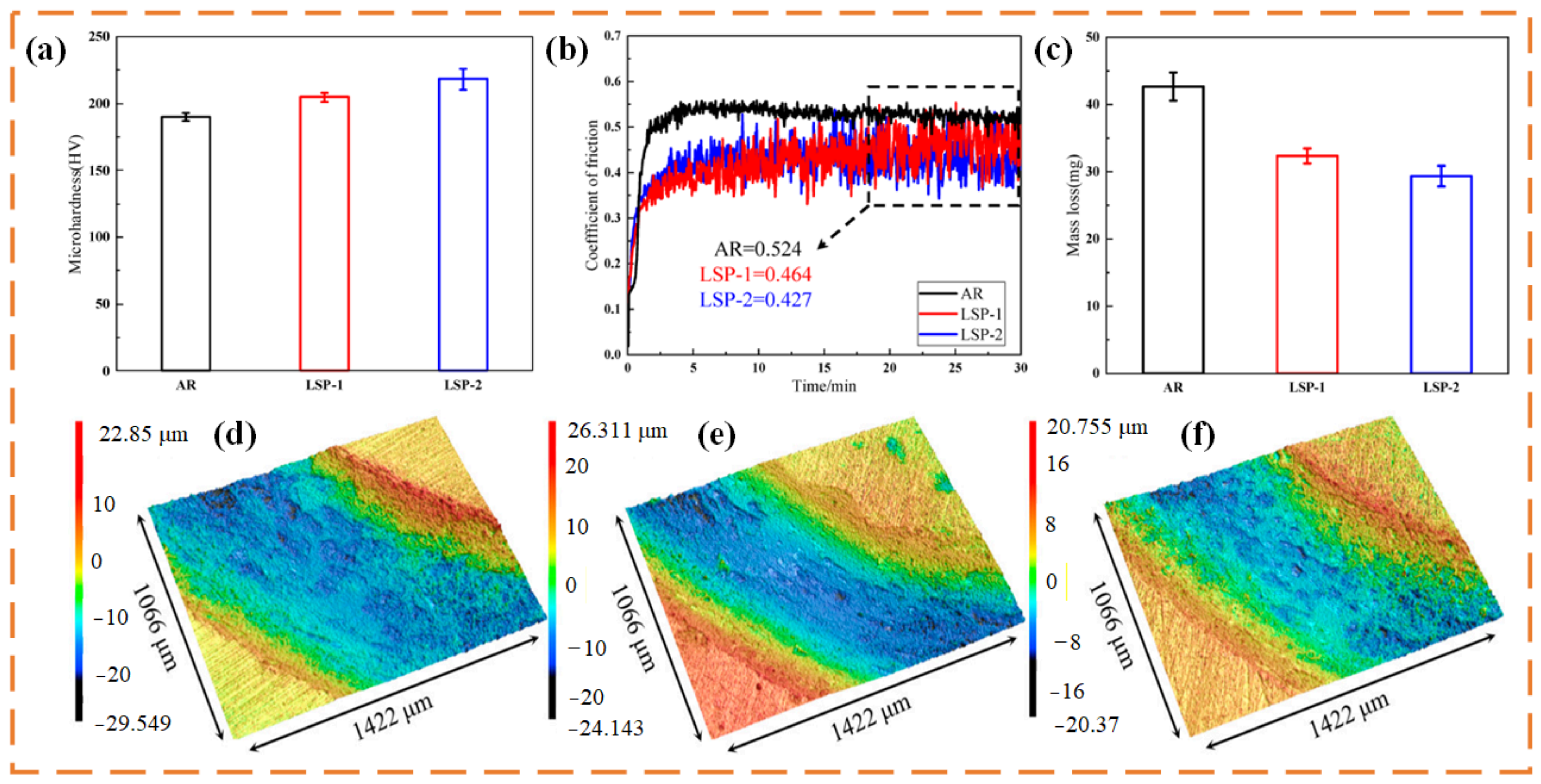
Microhardness and wear response of LSP-treated AH32 steel: (**a**) surface microhardness of untreated, LSP-1, and LSP-2 samples; (**b**) friction coefficient curves; (**c**) mass loss after sliding wear; (**d**–**f**) three-dimensional wear-track profiles of untreated, LSP-1, and LSP-2 samples. Panels are adapted from Ref. [134] under the terms of the Creative Commons Attribution License.

The carburized layer of 15CrNiMo steel illustrates the coupling between the hardness gradient and CRS depth. Wang et al. reported that LSP increased the surface microhardness by 20.5% and the average hardness within the top 850 μm by 18.8%, while producing a CRS layer approximately 1 mm thick [133]. In this carburized layer, the hardened region and the CRS layer overlap near the surface, thereby suppressing abrasive indentation, local plastic strain accumulation, and crack-opening tendencies under contact loading. A wear study on AH32 steel gives the tribological counterpart to this near-surface load-bearing mechanism, although it should be read as steel-based support rather than direct gear-steel evidence. As shown in Figure 14a, repeated LSP raised the surface microhardness from 190.2 HV in the untreated state to 204.7 HV after LSP-1 and 218.3 HV after LSP-2. The friction curves then shifted to lower steady levels, with the average coefficient of friction decreasing from 0.524 to 0.464 and 0.427 as the number of LSP passes increased (Figure 14b). This reduction was accompanied by lower material loss, with the LSP-2 sample giving the smallest mass loss after sliding (Figure 14c). The three-dimensional wear-track profiles provide the corresponding morphology evidence: compared with the untreated surface, the LSP-treated tracks became less deeply cut and less severe, especially after two passes (Figure 14d–f) [134]. Within this limited steel-based comparison, the data support the view that an LSP-hardened surface can reduce adhesive transfer and spalling during sliding, thereby promoting a milder abrasive-wear response.

M50 bearing steel further highlights hardening retention under thermal disturbance. After LSP, M50 steel retained higher hardness at both room and elevated temperatures, together with a CRS layer with a peak magnitude of approximately 1200 MPa and an affected depth of approximately 2 mm [135]. Component geometry [136], impact angle [137], and absorbing-layer condition can also alter shock-wave coupling and the distribution of the plastically deformed zone [138], leading to spatial variations in the hardened layer on curved surfaces or under oblique incidence. The absorbing-layer condition affects the transfer of impact energy into near-surface plastic deformation. When the energy input exceeds the stable processing range, local damage and excessive plastic deformation may reduce the hardness gain and disrupt the continuous transition of the hardness gradient [139,140].

The 15CrNiMo results establish the gear-steel basis of this subsection by linking the hardness gradient with a CRS layer extending to approximately 1 mm within the carburized near-surface region. The AH32 and M50 results further show that gradient-hardened layers can remain effective under frictional loading and thermal disturbance. The microhardness gradient reflects the combined effects of hardening magnitude, affected depth, and stability under load, and it influences subsequent wear, fatigue-crack initiation, and near-surface damage propagation.

#### 4.2.3. Surface Defect Sensitivity, Crack-Initiation Depth, and Damage Morphology

Under rolling–sliding contact and cyclic Hertzian stress, contact fatigue cracks on gear tooth flanks generally initiate through two main routes: surface-initiated pitting or micropitting [141], and subsurface-initiated spalling [142]. The former is more strongly governed by surface frictional shear stress and surface condition, whereas the latter usually originates near the subsurface cyclic shear-stress maximum and then propagates toward the surface [143]. Changes in surface and subsurface integrity can therefore alter the preferred crack-initiation depth and the associated damage path.

**Figure 15:**
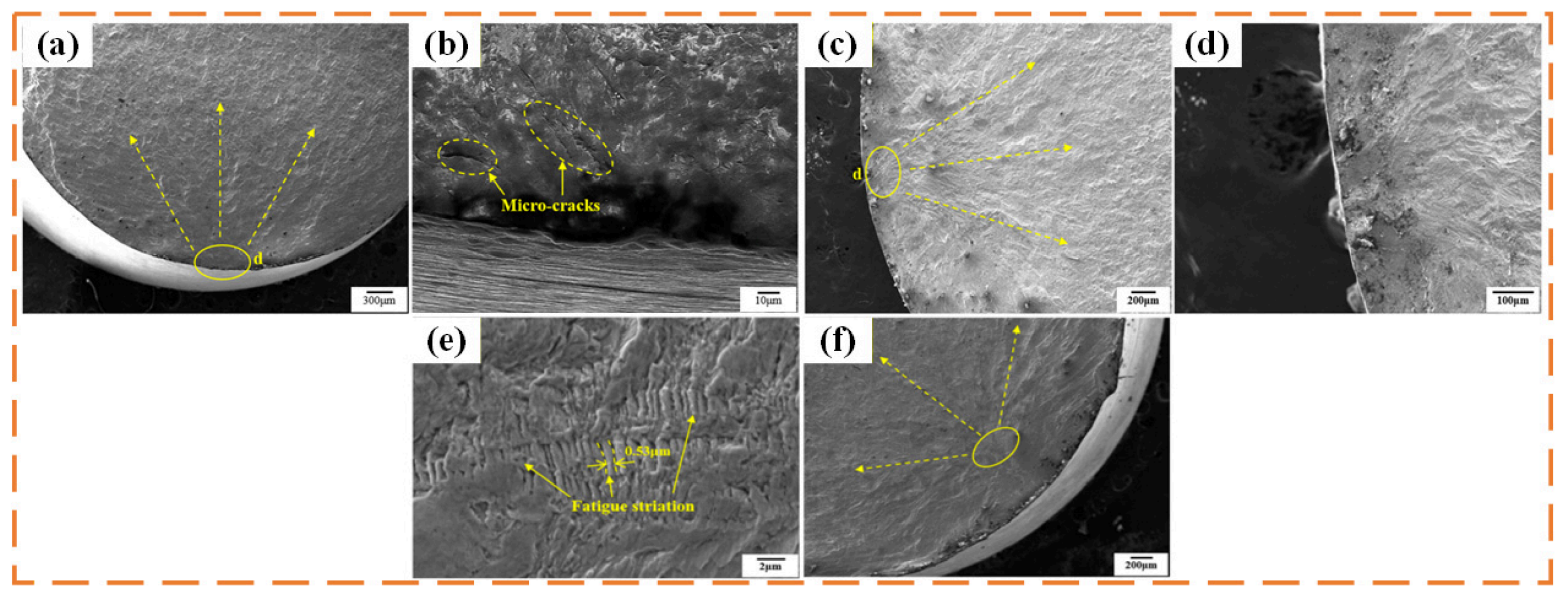
Fatigue fracture morphology and crack-initiation response after LSP: (**a**,**b**) surface-origin fatigue cracking and microcracks in untreated 1Cr12Ni3Mo2VN steel; (**c**) subsurface fatigue-source location after LSP at 3 J, where the region marked d indicates the fatigue source; (**d**) enlarged view of the fatigue-source region marked d in (**c**); and (**e**,**f**) mixed surface/subsurface fatigue initiation and finer fatigue striations after LSP at 4 J. Panels are adapted from Ref. [48] under the terms of the Creative Commons Attribution License.

After LSP, suppression of surface crack initiation and a shift of crack initiation sites toward the subsurface have been reported in gear steels and related high-strength steels. In 18CrNiMo7-6 gear steel, cracks after LSP were more likely to form beneath the surface [125]. Rotating-bending fatigue results for 1Cr12Ni3Mo2VN martensitic steel provide a useful fracture-morphology analogue. In the untreated sample, the fracture surface contained multiple surface fatigue sources; radial marks extended inward from the surface origin, and microcracks were visible near the initiation region (Figure 15a,b). After LSP at 3 J, one fatigue source appeared below the surface, and the fatigue-striation spacing decreased to about 0.63 μm, compared with about 1.12 μm in the high-frequency-hardened sample (Figure 15c,d). At 4 J, the fatigue source became mixed surface/subsurface in character, but the subsurface origin was still evident and the striation spacing further decreased to about 0.53 μm (Figure 15e,f) [48].

**Figure 16:**
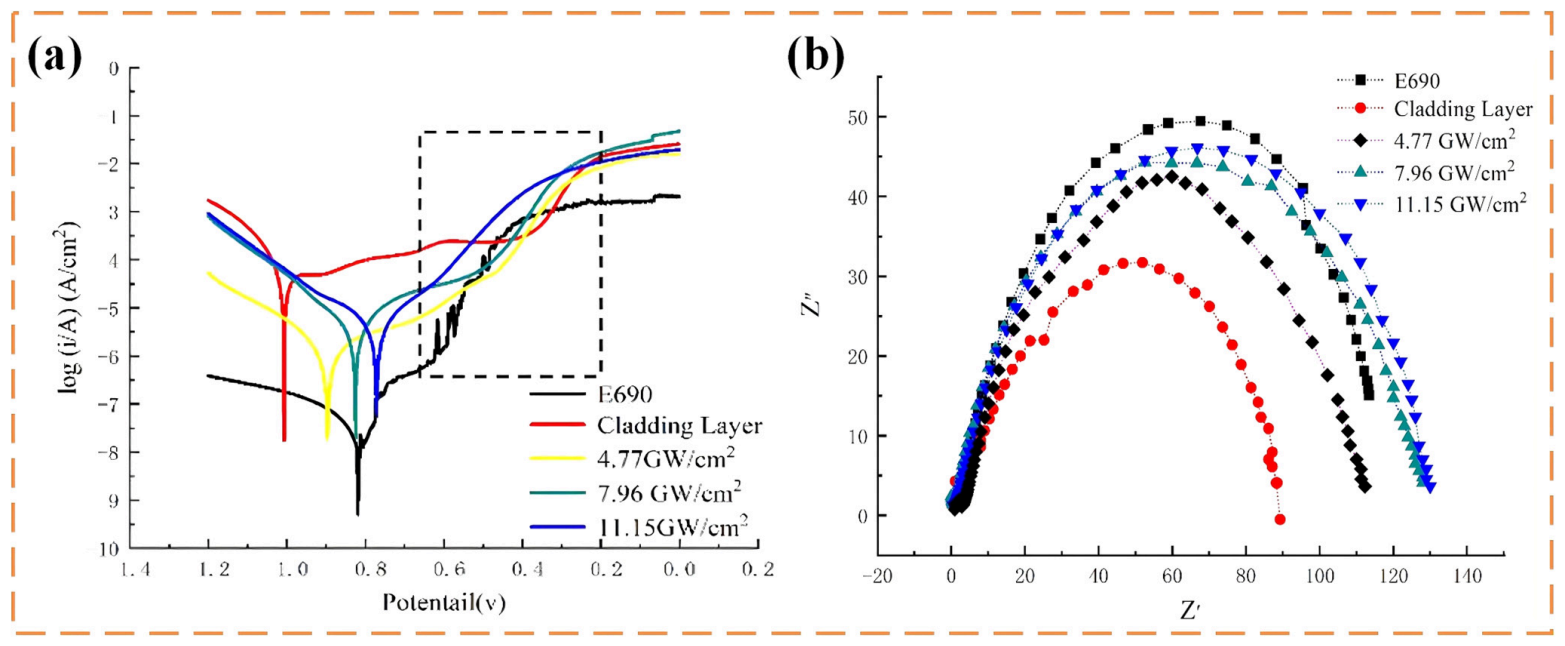
Electrochemical corrosion response of LSP-treated E690 high-strength steel cladding in 3.5 wt.% NaCl solution: (**a**) potentiodynamic polarization curves and (**b**) Nyquist plots. Panels are adapted from Ref. [153] under the terms of the Creative Commons Attribution License.

The change in crack-initiation depth is also related to the LSP-induced redistribution of residual stress, microstructural state, and defect condition from the surface to the subsurface region [45,144]. As the CRS layer extends inward, the local stress concentration at the tips of surface crack-like defects is reduced, thereby improving the tolerance of the surface layer to such defects [145]. As shown in Figure 16, after LSP treatment under a contact stress of 715 MPa, 316L stainless steel exhibited markedly less surface delamination, and the dominant damage mode changed from layer peeling to microplastic deformation [146].

Across the studies discussed here, the LSP benefit is concentrated near crack-source activation and short-crack growth. CRS and hardening make machining marks, pits, and shallow microcracks less prone to opening, so surface origins may move inward in pitting, micropitting, and bending-fatigue cases. The reduced striation spacing after LSP, together with the change from layer peeling to microplastic deformation, also suggests slower early growth while the crack remains inside the retained strengthened layer; this effect should weaken once the path leaves that layer. On tooth flanks, the controlling balance involves surface defect severity, stress-layer depth, hardening support, local microstructure, and the subsurface shear-stress maximum. Related evidence is listed in Table S1 [50,147,148,149,150,151,152].

#### 4.2.4. Passivation Stability and Environmental Damage

Contact fatigue and wear remain the primary damage modes of transmission gears, but humid, saline, or degraded-lubrication environments can turn wear scars, microcracks, and locally exposed surfaces into corrosion-active sites. E690 high-strength steel cladding provides a gear-related steel example for this environmental-damage boundary. In 3.5 wt.% NaCl solution, LSP shifted the corrosion potential of the cladding layer from −1.004 to −0.771 V and reduced the corrosion current density from 114.5 to 5.41 μA cm^−2^ as the laser power density increased to 11.15 GW cm^−2^ (Figure 16a) [153]. The Nyquist response followed the same trend: the capacitive-arc radius increased after LSP, indicating improved interfacial stability and lower corrosion activity (Figure 16b). This improvement was attributed to grain refinement, increased residual compressive stress, and a denser, more stable passivation film that reduced Cl^−^ attack and suppressed corrosion-microcrack propagation. When tooth-surface damage has already exposed fresh material, a more stable near-surface electrochemical state can slow corrosion-assisted growth of local defects under disrupted lubrication or aggressive service environments. Corrosion fatigue is a more severe form of this environmental boundary because cyclic crack opening is superimposed on pit formation, local dissolution, and CRS relaxation. In AISI 420 martensitic stainless steel, Wang et al. showed that massive laser shock peening treatment (MLSPT)MLSPT reduced the corrosion-fatigue crack-growth rate in NaCl solutions with different pH values; for gear-related assessment, the key point is that the benefit came from the combined action of a gradient CRS field and surface microstructural evolution, rather than from electrochemical stability alone [154]. Hydrogen-assisted effects may also become relevant when highly hardened gear steels are exposed to water-contaminated lubricants, humid environments, or other hydrogen sources. Evidence from L-DED 316L stainless steel suggests that LSP can improve hydrogen-embrittlement resistance [117], indicating that retained CRS and the LSP-modified near-surface microstructure may influence hydrogen-related cracking. For carburized or nitrided gear steels, this issue is more likely to appear through changes in crack origin, delayed or accelerated CRS relaxation, and hydrogen-sensitive fracture features during fatigue or RCF loading.

### 4.3. Effects of Surface-Integrity Changes on Gear Fatigue-Life Response and Damage Evolution

Gears operate under combined rolling–sliding contact and alternating bending loads. Tooth-flank contact fatigue and tooth-root bending fatigue are both governed by the near-surface stress state, microstructural stability, and crack-initiation susceptibility. Previous studies have shown that LSP can increase the fatigue limit and alter the S–N response [49]. The resulting life improvement is also influenced by the coverage of the strengthened layer, CRS retention, and service loading conditions [155]. In some gear steels and related materials, improved surface integrity is accompanied by higher wear resistance and slower early-stage damage growth [156].

#### 4.3.1. Effects of LSP on Gear Bending Fatigue Life and Crack Propagation

**Figure 17:**
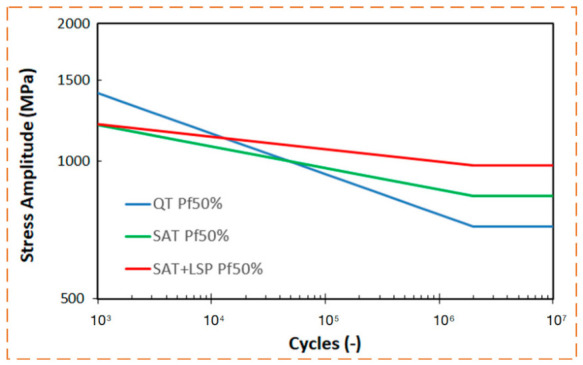
Bending-fatigue response of LSP-treated 54SiCr6 high-strength spring steel: comparison of mean S–N curves for quenching and tempering (QT), strain-assisted tempering (SAT), and SAT + LSP states obtained from three-point bending-fatigue tests. Panel is adapted from Ref. [157] under the terms of the Creative Commons Attribution License.

The improvement in tooth-root bending-fatigue life is governed by CRS coverage in the critical root-fillet region and by the surface-integrity state left after peening. In the absence of directly reusable gear-level S–N figures, three-point bending-fatigue data for LSP-treated 54SiCr6 high-strength spring steel offer a bending-dominated mechanical analogue. In that study, strain-assisted tempering (SAT) was followed by LSP, and the S–N mean curve shifted upward; the estimated fatigue limit increased from 837 MPa after SAT to 980 MPa after SAT + LSP (Figure 17) [157]. Although this spring-steel result is not a gear test, it follows the same bending-fatigue logic: a compressive near-surface layer suppresses surface-origin cracking on the highly stressed bending surface and redirects fatigue damage toward subsurface or internal defects.

Hybrid surface modification of AISI 9310 gear steel further emphasizes the role of surface-state control in bending fatigue. LSPwC followed by vibratory polishing reduced ablation and remelting features while retaining a work-hardened layer and a near-surface CRS field, leading to a much larger bending-fatigue improvement than LSPwC alone [132]. The benefit came from the combined control of compressive stress, surface defects, and near-surface microstructural stability, rather than from residual-stress magnitude alone.

The spatial match between the strengthened region and the fatigue-critical region further controls the life benefit. For 42CrMo, a steel relevant to heavily loaded gears, combined surface and side peening produced a longer fatigue life than surface peening alone. Although cracks could still initiate from edge corners, CRS coverage on the side surface delayed their initiation and early growth [158]. In 30CrMnSiNi2A steel, the crack origin shifted from the surface to the interior after LSP [47], consistent with the role of CRS in reducing the driving force for surface crack initiation. Stable fatigue-life improvement requires the CRS field and hardened layer to cover the local stress-concentration zone, particularly the tooth-root fillet and edge corners.

**Figure 18:**
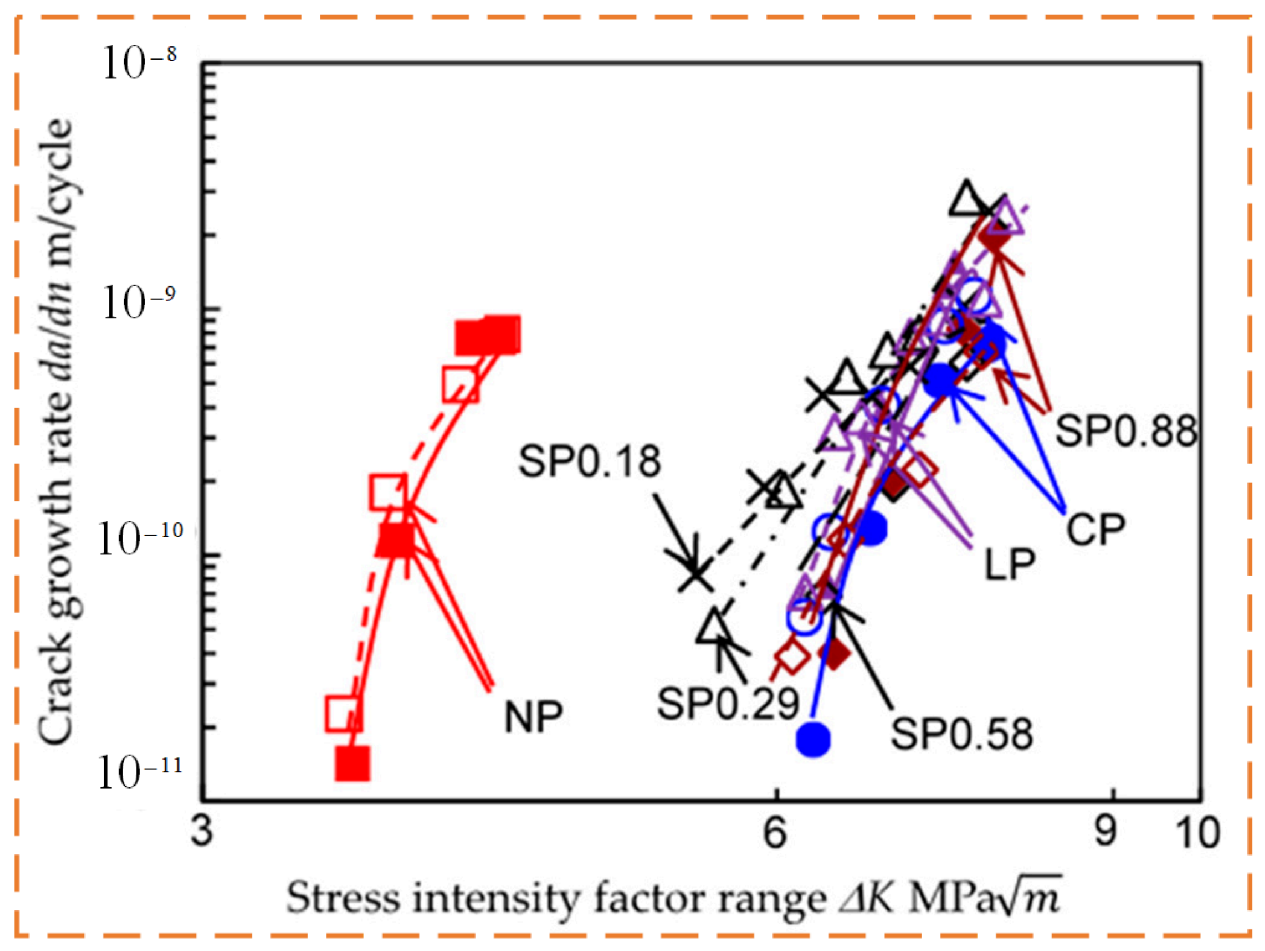
Fatigue crack-growth response of laser-peened JIS SUS316L austenitic stainless steel: Δ*K–da/dN* curves obtained by the *K*-decreasing test, showing the increase in near-threshold crack-growth resistance after peening. Panel is adapted from Ref. [159] under the terms of the Creative Commons Attribution License.

Microstructural stability under cyclic loading controls the persistence of the strengthening effect. Once a crack enters the stable-growth regime, CRS can still contribute by reducing the effective driving force at the crack tip. In 42CrMo4, another gear-related Cr–Mo steel, LSPwC produced a near-surface gradient structure consisting of a heat-affected layer, a severely plastically deformed (SPD) layer, and the substrate. Under cyclic loading, carbides, dislocation structures, and martensitic laths in the SPD layer remained relatively stable, which limits local microstructural degradation [47]. Crack-growth data for laser-peened JIS SUS316L austenitic stainless steel provide a steel-based fracture-mechanics analogue. As shown in Figure 18, the Δ*K–da/dN* curve shifted to higher Δ*K* values after laser peening, and the fatigue crack-growth threshold increased from 3.71 ± 0.11 to 6.33 ± 0.20 Mpa m^1/2^ [159]. This shift indicates that a larger stress-intensity-factor range was required to sustain near-threshold crack growth after peening, consistent with a reduced effective crack-tip driving force.

Fractographic results for 1Cr12Ni3Mo2VN and 25Cr2Ni4MoV further show that LSP reduced the fatigue striation spacing, indicating a smaller crack advance per cycle and a lower stable crack-growth rate [48,160]. Representative results on bending-fatigue life, crack-origin location, and crack-growth behavior in gear steels and related high-strength steels after LSP are summarized in Table S2 [47,132,157,158,159,160,161].

Accordingly, LSP enhances the bending-fatigue resistance of gear steels by combining CRS coverage in the critical tooth-root region with improved surface topography and a stabilized near-surface gradient microstructure. The CRS layer lowers the crack-opening driving force and reduces the effective stress-intensity-factor range, while improved surface topography diminishes potential crack nuclei such as ablation pits, remelted layers, and sharp asperities. The gradient microstructure further retards local cyclic plasticity. When these strengthened regions cover critical features such as tooth-root transitions and edge corners, the effects are manifested as an upward shift of the S–N curve and a reduced crack-growth rate.

#### 4.3.2. Influence of Surface Integrity on Tooth-Flank Contact Fatigue and Wear Damage Evolution

**Figure 19:**
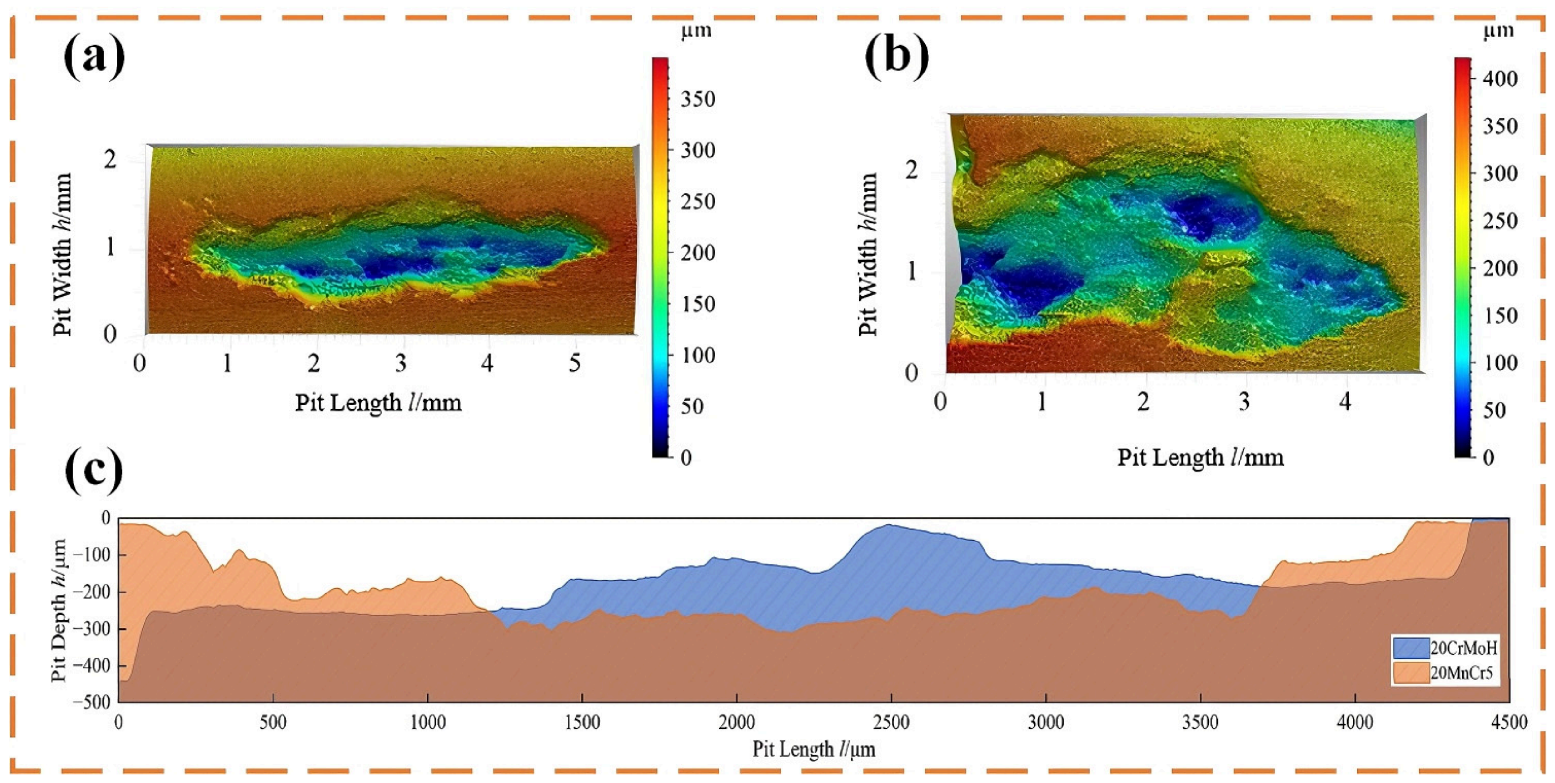
Three-dimensional pitting morphologies: (**a**) pitting on the 20MnCr5 gear; (**b**) pitting on the 20CrMoH gear. (**c**) pit cross-sectional profiles of the 20MnCr5 and 20CrMoH gears. Reproduced from Ref. [124] under the terms of the Creative Commons Attribution license.

In rolling–sliding tooth-flank contact, micropitting and spalling are associated with different damage-driving conditions. Micropitting is closely linked to asperity-scale surface contact, whereas spalling is more strongly controlled by the subsurface stress field. A gear model based on 8620H steel showed that realistic surface roughness, the near-surface stress field, and the hardness gradient can change the site of microcrack initiation and the pitting risk near the pitch line [48]. Local residual-stress modeling further showed that surface CRS increases the fatigue crack-initiation limit and shifts microcrack initiation toward the subsurface region [162]. A comparison between 20MnCr5 and 20CrMoH gear steels demonstrated that the hardness gradient, CRS retention, and microstructural stability affect the severity of micropitting and spalling. As shown in Figure 19, a gentler hardness gradient, stable CRS, and cyclic transformation of retained austenite help maintain shallow micropitting. Rapid residual-stress relaxation and insufficient support from the hardened gradient, by contrast, increase the susceptibility to deeper spalling [124].

**Figure 20:**
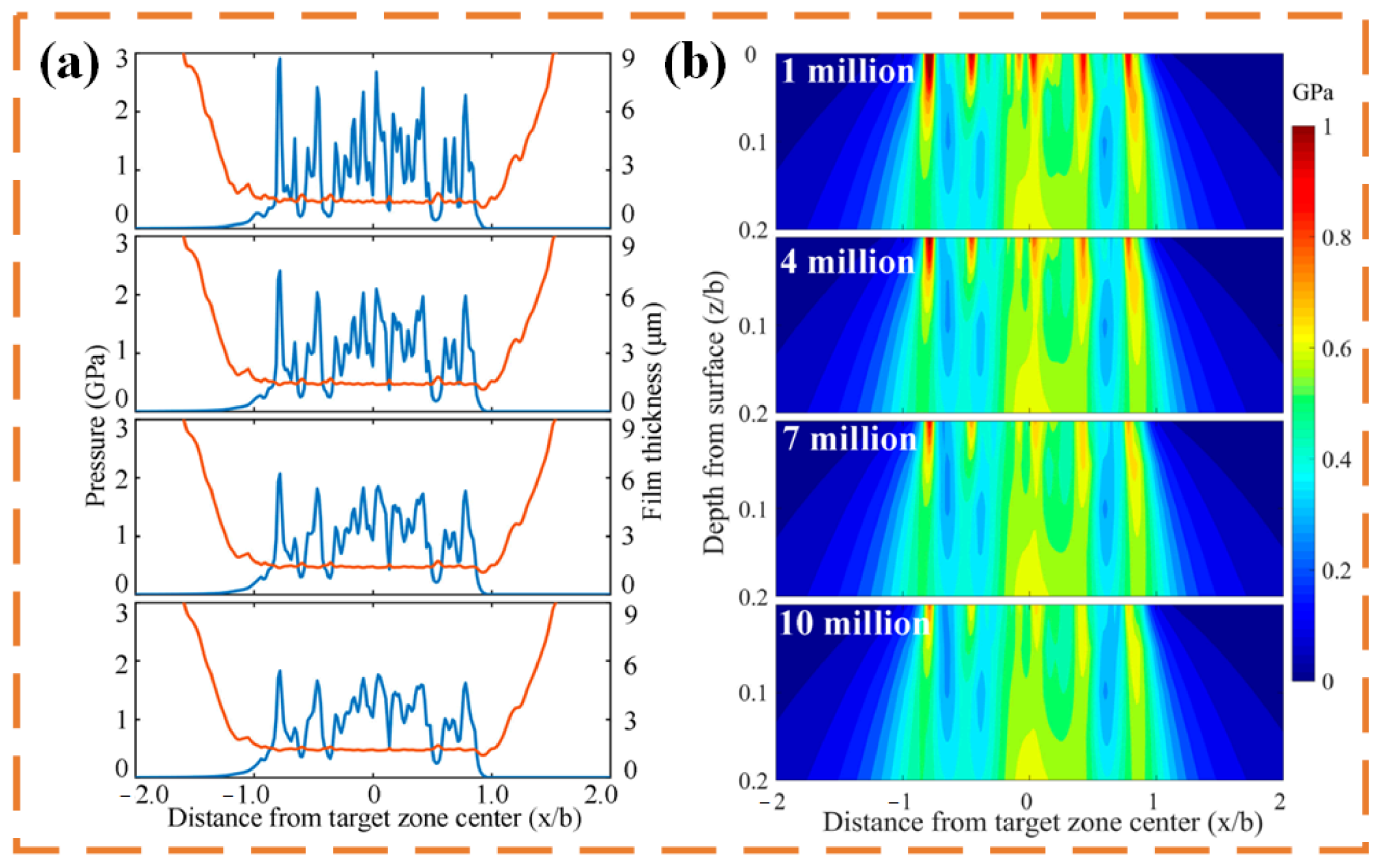
Mixed-EHL modelling of steel gear micropitting under rough sliding and mild wear, showing reduced pressure peaks and near-surface stress concentration over 10 million cycles: (**a**) pressure and film-thickness distributions, where the blue curves represent pressure and the red curves represent film thickness; (**b**) von Mises stress distribution.Panel is adapted from Ref. [131] under the terms of the Creative Commons Attribution License.

Mixed-EHL modeling of steel gear micropitting supports the surface-controlled side of this distinction. Mild wear progressively removed asperity peaks, reducing the maximum pressure from nearly 3 GPa after 1 million cycles to about 1.8 GPa after 10 million cycles and markedly weakening the near-surface von Mises stress concentration (Figure 20) [131]. For tooth-flank LSP, the CRS layer should still reach the vicinity of the subsurface shear-stress maximum associated with spalling, but post-peening topography must also be controlled so that asperities, local pits, or ablation marks do not become new micropitting initiation sites. Mixed-lubrication tests on case-carburized AISI 8620-type rollers add the material-state side of this problem. Paladugu and Hyde showed that, under mixed lubrication, fine martensitic microstructures gave nearly threefold longer rolling-contact life than coarse microstructures; RCF also reduced near-surface retained austenite and produced different subsurface residual-stress redistribution [163]. The implication is that a peened tooth flank should not be judged only by its initial CRS depth, but also by how film formation, asperity contact, retained-austenite stability, and micropitting/spalling morphology evolve during lubricated rolling contact.

**Figure 21:**
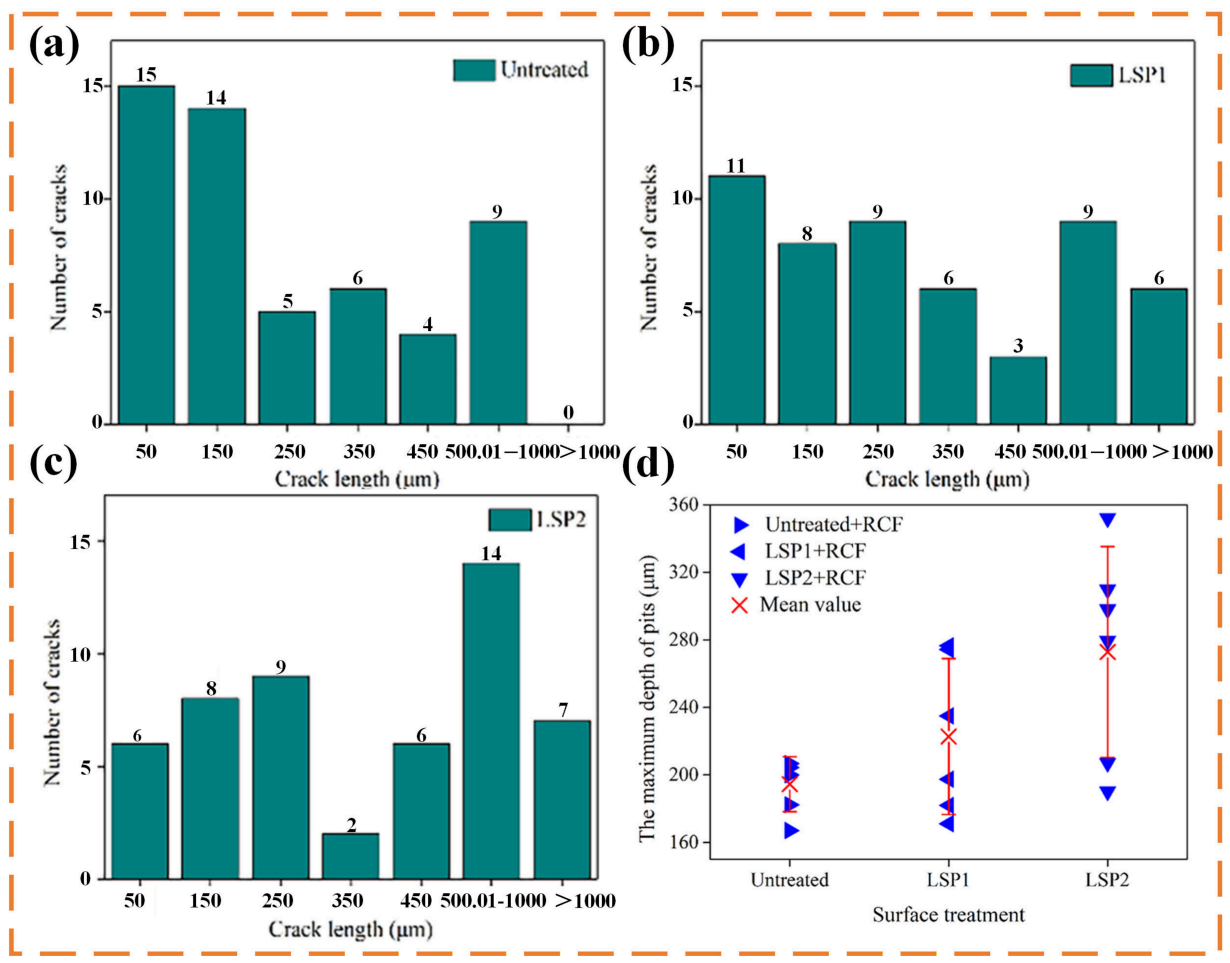
Fatigue crack-length distributions in the wheel discs at failure: (**a**) untreated, (**b**) LSP1, and (**c**) LSP2; (**d**) pit depth in the wheel discs at failure. In panel (**d**), the red crosses denote the mean values, while the red vertical lines indicate the corresponding variability ranges. Panels (**a**–**d**) are reproduced from Ref. [164] under the terms of the Creative Commons Attribution license.

Twin-disc tests on ferrite–pearlite steel provide additional evidence that LSP can alter crack paths under rolling contact. As shown in Figure 21a–c, the hardened layer after LSP extended to the millimeter scale, and the surface CRS approached 1 GPa. The untreated sample failed after 61,190 cycles, whereas the LSP1 and LSP2 samples failed after 69,032 and 88,537 cycles, respectively. Changes in crack inclination, crack-length distribution, and pit depth indicate that CRS and strain hardening redirected near-surface crack propagation (Figure 21d) [164]. High-load wear tests on H13 steel also showed that grooves, lamellar spalling, and microcutting marks were reduced after LSP [165].

The strengthening response of LSP-treated tooth flanks is ultimately controlled by the depth and continuity of the CRS layer, the support provided by the hardened layer, and the quality of the post-peening surface topography. The CRS field needs to reach the subsurface shear-stress maximum, where it can reduce crack opening and retard spalling-crack growth. The hardened layer must provide a continuous load-bearing gradient capable of accommodating near-surface plastic deformation under rolling–sliding contact. At the same time, the treated surface should not introduce asperities, grooves, or local pits that can serve as new micropitting initiation sites. When these stress, hardening, and topographical features are aligned with the depth and spatial distribution of the tooth-flank damage-prone region, the near-surface state produced by LSP can be expressed as longer RCF life, delayed micropitting, slower spalling growth, and lower crack sensitivity under wear-coupled contact.

#### 4.3.3. Service Stability of LSP-Induced CRS

CRS measured after LSP represents the initial strengthened state rather than the effective stress field retained during long-term service. Thermal exposure can weaken or redistribute CRS through recovery, dislocation rearrangement, phase transformation, and changes in microstructural stability; cyclic contact or bending loads can further promote relaxation through local cyclic plasticity, crack-tip stress release, and stress rebalancing [135,159,161]. Thus, the fatigue benefit of LSP should not be judged only by the initial surface CRS peak, but by whether the compressive layer retains sufficient depth, continuity, and overlap with the damage-prone region.

Wear-induced relaxation is more direct in rolling–sliding tooth-flank contact. Mild wear may remove asperity peaks and reduce local contact pressure, whereas sustained wear, micropitting, or spalling can consume the CRS-bearing surface layer and alter the subsurface shear-stress field [124,131]. From this viewpoint, LSP-treated tooth flanks should be evaluated by the retained CRS depth after wear and its match with the subsurface shear-stress maximum, while tooth-root regions should be evaluated by whether CRS continuity remains after bending cycles.

### 4.4. Summary

The fatigue and wear response of LSP-treated gear materials is governed by the coupled evolution of surface topography, CRS fields, hardness gradients, and microscopic defects. Within an appropriate process window, LSP can develop a stable near-surface compressive stress layer and a gradient-hardened structure, reduce the tendency of surface crack-like defects to open and grow, and shift crack initiation toward the subsurface. If the parameter combination is inappropriate, or if confinement and surface protection are not well controlled, increased roughness, local pitting, and thermally induced defects can offset the strengthening effect and reintroduce surface crack-initiation sites.

LSP-treated tooth flanks require stable post-peening topography, continuous load-bearing support from the hardened layer, and extension of the CRS layer toward the subsurface shear-stress maximum to delay micropitting initiation, spalling growth, and wear-assisted crack sensitivity. At the tooth root, the treatment should ensure CRS coverage of high-stress-concentration features, including the tooth-root transition and edge corners, while maintaining compressive-stress continuity and crack-growth resistance under cyclic loading. For long-term service, this assessment should further consider CRS relaxation and redistribution caused by thermal exposure, cyclic loading, and wear-induced surface removal, rather than relying only on the initial post-peening CRS peak.

Two issues are worth noting. The first is evidential: most available data still come from bending fatigue or crack-growth tests, whereas lubricated contact, micropitting, and post-wear stress/topography measurements remain much thinner for LSP-treated gear steels. The second is methodological. Future work should follow the same specimen or component through peening, lubricated rolling–sliding, post-test residual-stress/topography measurement, and crack-origin analysis; otherwise, improvements in initial CRS or fatigue life remain difficult to compare across studies.

## 5. Characteristics and Applicability of Stand-Alone Surface-Strengthening Routes

The preceding analysis linked LSP-induced changes in surface topography, CRS, hardness gradients, and defect sensitivity to the fatigue responses of tooth flanks and tooth roots [62]. For any individual strengthening route, however, the effective depth of action and the failure modes it can address must be defined more explicitly [166]. On this basis, different surface-strengthening routes are compared here in terms of surface crack-initiation control, load-bearing layer formation on tooth flanks, subsurface stress regulation, and long-term stability in gear damage-prone regions.

### 5.1. Subsurface Stress Regulation by Stand-Alone LSP

LSP can introduce a relatively deep CRS layer beneath the material surface [99], together with deformation hardening and microstructural refinement [56]. These changes raise the threshold for crack initiation and slow the early development of fatigue damage [55]. Direct evidence for this effect has been obtained under gear RCF conditions [167]. When LSP is used as a single treatment, its principal value lies in deep compressive-stress development, the inward shift of crack origins, and the reduction in crack-growth driving force.

**Figure 22:**
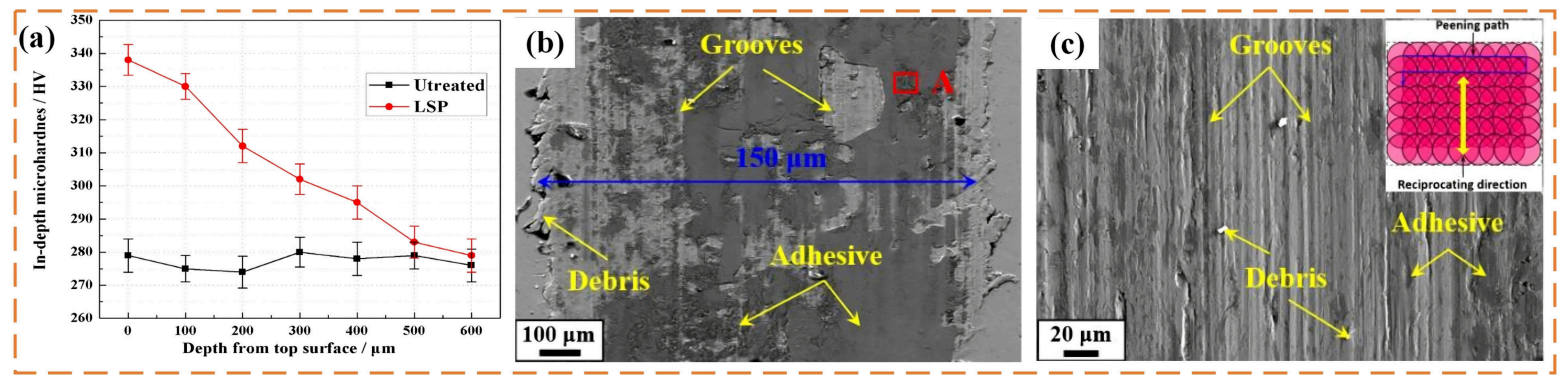
Hardening and wear response of LSP-treated 40CrMo steel: (**a**) depth-direction microhardness profiles before and after LSP; (**b**,**c**) worn surface morphologies of the untreated and LSP-treated samples. The red box labeled A in panel (**b**) indicates the region enlarged and shown in panel (**c**). Panels are adapted from Ref. [31] under the terms of the Creative Commons Attribution License.

Results for 40CrMo steel further delineate the wear-resistance contribution of stand-alone LSP in a gear-related Cr–Mo steel. After LSP, the surface microhardness increased from approximately 278 HV to 338 HV, and the hardened influence layer extended to about 600 μm (Figure 22a) [31]. Reciprocating wear tests showed that the mass loss decreased from about 167 mg to 121 mg, corresponding to a reduction of 27.5%. The wear-scar width also decreased from approximately 150 μm in the untreated sample to about 105 μm after LSP. Consistent with this reduction, the untreated surface exhibited deeper and denser grooves, whereas the LSP-treated surface showed only slight grooves (Figure 22b,c). The improved wear response was mainly associated with the higher surface hardness and LSP-induced microstructural strengthening, including increased dislocation density and deformation twins. Even so, the result should be read as a limited wear-resistance gain from stand-alone LSP, because severe contact conditions still require additional control of surface topography and material removal.

LSP alone is therefore more effective for subsurface stress regulation and fatigue-resistance improvement than for sustained surface wear control. Under high-load contact, its ability to maintain surface wear resistance and long-term stability remains limited [168,169].

### 5.2. Surface Plasticity and Crack-Initiation Control in Mechanical Surface Strengthening

Mechanical surface-strengthening methods, such as shot peening (SP), ultrasonic surface rolling processing (USRP), and deep rolling, act primarily by imposing plastic deformation on the surface and near-surface layers. The resulting CRS, work hardening, and microstructural refinement mainly suppress surface crack initiation and early short-crack development. In situ fatigue observations in martensitic spring steel show that mechanical strengthening lowers the density of short-crack networks and moves the critical crack origin from the surface to depths of about 130–160 μm [170].

These methods differ chiefly in how they modify surface topography, residual-stress gradients, and affected depth. USRP improves surface hardness and residual-stress distribution while promoting dislocation multiplication and microstructural refinement [171,172]. Shot peening introduces surface plasticity and CRS rapidly through discrete particle impacts; however, it can also leave sharp asperities and local dimples that participate in subsequent contact damage. In 42CrMoA steel, for example, stronger hardening was accompanied by degraded surface topography [173]. Deep rolling uses continuous rolling contact to produce a smoother surface and a more continuous stress gradient, which helps reduce surface stress concentration [174,175,176].

Even so, the affected zone in these treatments remains concentrated in the surface and near-surface layers, and the depths of CRS and hardening are usually limited [177]. Mechanical strengthening is most effective when tooth-flank damage is governed by surface micropitting or shallow short cracks. Its capability becomes constrained when the critical damage process shifts to the subsurface shear-stress maximum, deep crack-driving zones near the tooth-root transition, or long-term residual-stress retention [4,178,179].

Shot peening is usually selected first in gear production because its equipment base, media delivery, coverage practice, and finishing checks already fit batch manufacture [19]. Surface dimples and shallow CRS/hardening can still limit lubricated flank use [173,177]. LSP is better reserved for roots, repaired or additively manufactured regions, or local zones requiring deeper compression [180]. Its repeatability depends on pulse/spot stability, confinement, absorbing-layer condition, and profile verification. Whole-surface production therefore favors shot peening, while LSP is justified when local CRS depth offsets the added control burden [179].

### 5.3. Microstructural Gradients and Load-Bearing Layer Development in Carburizing and Nitriding

Carburizing and nitriding are thermochemical diffusion treatments involving thermal cycling. In gears, the load-bearing capacity of the tooth flank derives mainly from the coupling between the surface diffusion case, the hardness gradient, and the tough core matrix [181]. The hardness gradient, retained-austenite stability, precipitate distribution, and residual-stress state jointly determine the resistance of the load-bearing layer to indentation, plastic deformation, and wear [182]. In carburized AISI 8620 steel, RCF life was improved when a higher retained-austenite content acted together with a higher CRS level [183].

The performance of nitrided layers is more closely tied to the structure of the compound layer and the diffusion layer. Nitride precipitation in the diffusion layer increases near-surface hardness and load-bearing capacity, while the compound layer governs surface wear resistance and contact reliability. If the compound layer becomes too thick, or if the ε-phase fraction is excessive, surface toughness decreases and contact fatigue reliability is compromised [184]. In this sense, thermochemical treatments are more suitable for developing tooth-flank load-bearing layers, hardness gradients, and microstructural stability. Relative to LSP, their independent ability to tailor deep subsurface CRS fields is weaker, and their control over tooth-root crack-driving forces and subsurface stress redistribution remains limited [185].

### 5.4. Depth of Action, Industrial Applicability, and Complementary Design of Individual Strengthening Routes

Mechanical surface strengthening, carburizing/nitriding, and LSP cover different parts of the gear damage zone. Mechanical treatments mainly act on surface crack sources, carburizing and nitriding build the tooth-flank load-bearing case, and LSP modifies the subsurface stress field and crack-growth driving force [180,186,187,188]. Their depth ranges and main applicability limits are summarized in Table S3 [19,20,170,171,172,173,174,175,176,177,179,186,187,188,189,190,191].

This depth distinction becomes important once manufacturability is considered. Established peening/finishing routes remain the production baseline for many gears because they are cheaper, faster, and easier to integrate into batch processing, although polishing or superfinishing may still be needed when shot-peened dimples affect lubricated flank contact. Local LSP is more relevant for tooth-root fillets, repaired or additively manufactured regions, and contact-damage zones that require additional subsurface stress regulation after carburizing or nitriding. Its use is limited by scanning access, beam incidence, absorbing-layer renewal, water confinement, and flank-form verification. Tool-contact routes, including ultrasonic nanocrystal surface modification (UNSM)/ ultrasonic surface rolling process (USRP), surface mechanical attrition treatment (SMAT), deep rolling, and burnishing, face similar access limits in tooth gaps, small modules, and inner features. Laser surface hardening is included as a heat-based comparator because its benefit comes from martensitic hardening rather than shock-induced CRS, while distortion and finishing allowance restrict its use on precision gears.

A quantitative comparison of representative LSP studies is provided in Table S4, covering laser parameters, CRS/hardening response, fatigue/RCF or wear performance, and roughness/topography. Table S4 also distinguishes actual gear or gear-like component evidence from evidence obtained using gear-steel simplified specimens and analogue materials, and lists the main limitation of each study [31,49,103,112,113,114,128,129,133,134,135,157,167,192,193,194,195,196].

### 5.5. Relative Contributions of LSP-Induced Strengthening Mechanisms

Among the changes introduced by LSP, the residual-stress field remains the clearest indicator for interpreting fatigue improvement. In tooth-root bending fatigue and surface-initiated high-cycle fatigue, compressive residual stress lowers the effective tensile stress and suppresses crack opening, as shown in LSP-treated AISI 9310 gears [49]. This effect is easily overstated, however, when only the peak surface value is considered. For gear steels, the more relevant issue is whether the compressive layer is deep, continuous, and stable enough to overlap the fatigue-critical region, as indicated by the CRS-depth response of 20CrMnTi steel [112], and the stress-field uniformity effect in 20CrNiMo steel [113].

Work hardening is more local. It improves resistance to indentation and cyclic plastic strain accumulation near the surface, so it becomes important in rolling–sliding contact, micropitting, and wear-assisted fatigue. This interpretation is consistent with the hardness-gradient response of carburized 15CrNiMo steel [125] and the reduced material loss of LSP-treated AH32 steel [126]. It should still be viewed as a supporting mechanism: hardening cannot compensate for insufficient CRS depth, and its benefit may be weakened by roughening or local contact defects.

Grain refinement is closely tied to dislocation evolution. Repeated LSP increases local misorientation and grain fragmentation in EBSD observations [117], while TEM results show dislocation cells, subgrain subdivision, and local fine grains [118]. Dislocation multiplication is therefore not a separate fatigue-strengthening source in the same sense as CRS; it links plastic deformation, hardening, and refinement. Dislocation-density-based modeling further supports this link [197]. Because these variables change together with LSP parameters and are affected by fatigue mode, damage depth, stress relaxation, and surface integrity, a transferable quantitative contribution ratio is unlikely. Differences among reported LSP trends should therefore be compared with caution, because a parameter window that suppresses surface-crack initiation may not give the same benefit for subsurface RCF, wear-assisted damage, or long-term CRS retention.

### 5.6. Summary

Each single strengthening route operates over a characteristic depth range and modifies a different part of the near-surface state. LSP provides relatively deep CRS support and mainly reduces the driving force for crack growth. Mechanical surface strengthening relies on near-surface plastic deformation and is more effective in suppressing surface crack initiation and early short-crack growth. Carburizing and nitriding form a high-hardness diffusion case that supports tooth-flank load bearing. These routes therefore differ in surface crack-source control, tooth-flank load-bearing support, subsurface stress regulation, and long-term stability.

Different damage-prone regions in gears call for different strengthening responses. Asperity contact and early surface cracking require control of surface topography and suppression of shallow crack-source activity. Tooth-flank resistance to plastic deformation is supported by a continuous hardness gradient and a stable microstructure. Subsurface spalling and tooth-root bending fatigue require a deep and stable CRS field to retard crack growth.

## 6. Hybrid Strengthening Routes and Synergistic Control Mechanisms

The benefits and limits of individual strengthening routes lead naturally to hybrid strategies that combine LSP with thermochemical treatment, mechanical strengthening, heat treatment, or surface texturing [198,199]. The effects of different hybrid routes are therefore evaluated from three aspects: the characteristics of gradient-strengthened layers, the evolution of surface integrity, and changes in crack-propagation paths [191,200]. Because studies on hybrid LSP in gear steels remain limited, the discussion gives priority to gear steels and related load-bearing steels, while evidence from non-gear metallic materials is used to clarify diffusion, phase transformation, microstructural gradients, and interfacial control in hybrid strengthening.

### 6.1. Coupling Between LSP and Thermochemical Surface Modification for Gradient-Strengthened Layer Development

Hybrid routes combining LSP with thermochemical surface modification have mainly been developed along two lines [166]. One concerns the processing sequence between LSP and diffusion treatment, including pre-LSP-assisted diffusion and post-LSP re-strengthening [133,198]. The other concerns the final architecture of the strengthened layer, where an LSP-induced structural gradient is superimposed on a chemically graded case [201]. The following analysis therefore follows this sequence: pre-LSP-assisted diffusion and case growth, post-LSP modification of existing diffusion layers and compressive-stress enhancement, and the coupled development of chemical and structural gradients.

#### 6.1.1. Pre-LSP-Assisted Diffusion and Case Growth

Pre-LSP can promote thermochemical diffusion by introducing dislocations, subgrain boundaries, and near-surface plastic deformation before carburizing or nitriding. These defects provide short-circuit diffusion paths for carbon or nitrogen and may extend the diffusion case or effective hardened layer. In 20Cr2Mn2Mo steel, pre-LSP increased the hardened layer formed by low-temperature vacuum carbonitriding from approximately 170 μm to 320 μm, but excessive laser energy or repeated impacts increased surface roughness, raising the coefficient of friction and wear severity again [198]. AISI 9310 steel showed the same diffusion-enhancement tendency: pre-LSP increased the carburized layer thickness from approximately 10 μm to 34 μm through dislocation walls, deformed grains, high-angle grain boundaries, and reduced carbon-diffusion activation energy [202]. After subsequent vacuum carbonitriding, part of the LSP-induced surface CRS could relax, but subsurface compressive stress and defect-assisted strengthening were still retained, with Orowan and dislocation strengthening making major contributions [203].

Studies in other steel systems are consistent with this trend. In M50 steel, LSP pretreatment promoted the growth of nitrided layers or ion-implantation-modified layers and improved hardness, compressive stress, and wear response [204,205]. Plasma nitriding studies on 42CrMo and H13 steel likewise showed that the defect structures and refined microstructures introduced by pre-LSP favored the growth of the compound layer or effective hardened layer [166,206]. Results for 17-4PH stainless steel further indicate that excessive overlap or unsuitable spot conditions can cause surface ablation and roughening, thereby weakening the benefit of the hybrid treatment [207].

Thus, pre-LSP is useful for diffusion-case growth only within a controlled surface-state window. It can deepen the case through defect-assisted diffusion, but excessive peening damage may offset this benefit through roughness-driven friction and wear. Representative routes and key findings are summarized in Table S5 [166,198,202,203,204,205,206,207].

#### 6.1.2. Post-LSP Re-Strengthening and Gradient-Structure Control in Pre-Existing Diffusion Cases

After carburizing or carbonitriding, the near-surface region of gear steels contains a diffusion case with depth-dependent chemistry, phase constitution, hardness, and residual stress. At this stage, the shock pulse meets a hardened diffusion case rather than an initially uniform surface. For highly hardened cases, the additional hardness increment after LSP can be limited; changes in CRS distribution, retained-austenite stability, defect structure, and crack-origin behavior may be more relevant to fatigue control.

**Figure 23:**
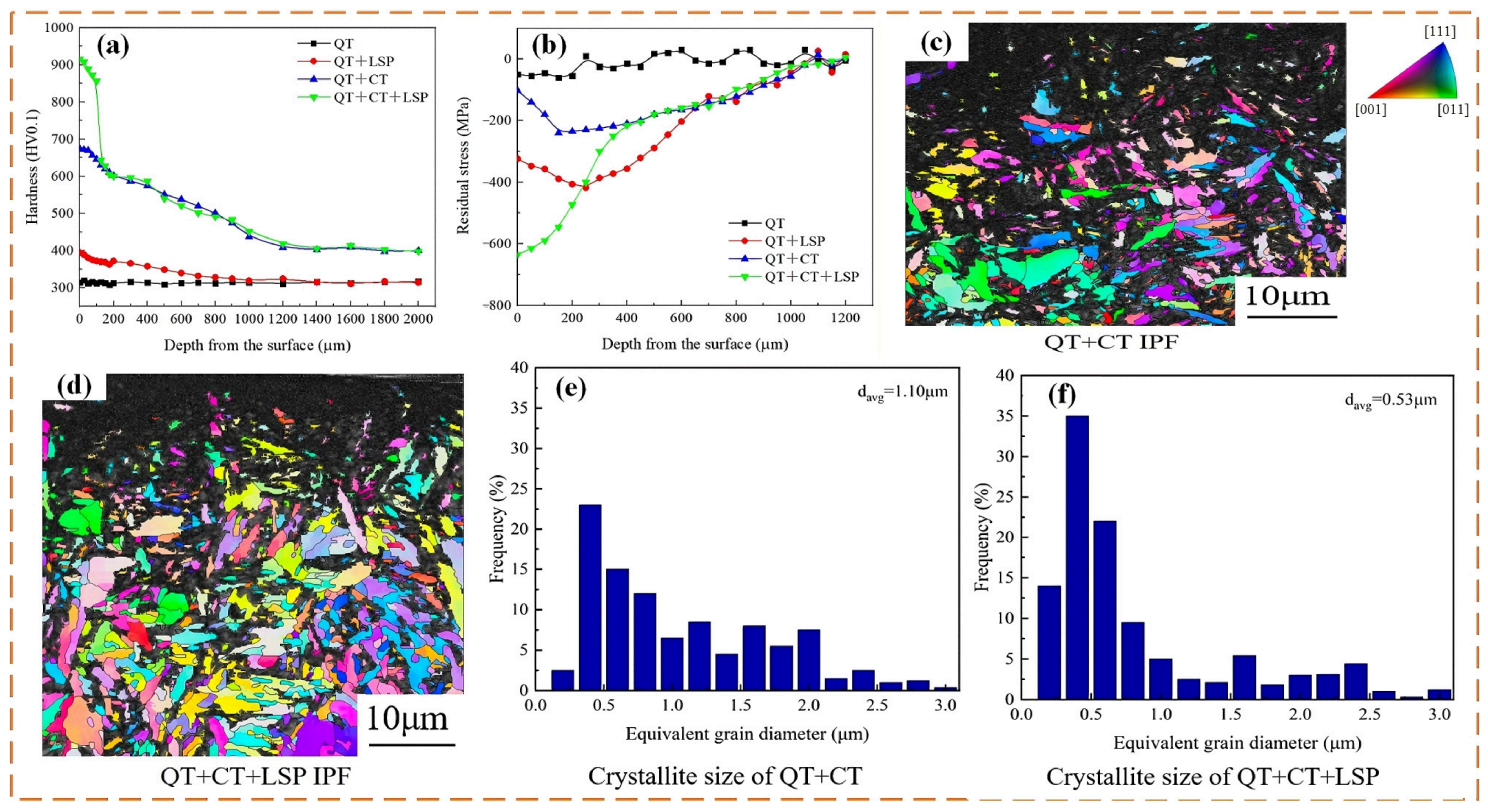
Surface properties and microstructural evolution of QT + CT and QT + CT + LSP samples: (**a**) surface hardness; (**b**) residual stress; EBSD inverse pole figure (IPF) maps of (**c**) QT + CT and (**d**) QT + CT + LSP samples; grain-size distributions of (**e**) QT + CT and (**f**) QT + CT + LSP samples. Panels (**a**–**f**) are reproduced from Ref. [50] under the terms of the Creative Commons Attribution license.

The QT–CT–LSP route in 20CrNiMo steel provides a representative example for gear-related steels. After carburizing, the surface microhardness increased from 314 HV_0.1_ in the QT substrate to 674 HV_0.1_; after LSP, it further reached 916 HV_0.1_. The surface CRS magnitude also increased from −103 MPa to −635 MPa (Figure 23a,b). EBSD results revealed further refinement in the surface region of the carburized layer after LSP, together with retained-austenite transformation into martensite (Figure 23c–f) [50]. This case indicates that, under a specific case state and processing condition, post-LSP can modify the stress state, surface microstructure, and phase constitution of a carburized layer. The magnitude of the hardness increment, however, should not be generalized without considering the prior case condition.

Another carburizing–LSP study on 20CrNiMo steel further links post-LSP modification to fatigue response. The bending-fatigue life increased from 7.3 × 10^6^ cycles to 3.83 × 10^6^ cycles, but decreased again when the impact number was further increased [192]. Fracture observations showed that the crack origin shifted from machining marks on the carburized surface to subsurface or internal inclusions, accompanied by fish-eye features. In this case, the main role of post-LSP was not simply to increase hardness, but to adjust the stress field and the dominant crack-origin population within the pre-existing case.

Similar gradient-superposition behavior has also been reported in diffusion-treated steels outside the gear-steel literature. For carburized EX30 bearing steel, subsequent LSP introduced a nanocrystalline gradient and CRS within the pre-existing compositionally graded case, while improving hardness, wear resistance, and the graded strain distribution [201].

On this evidence, post-LSP is better described as a secondary regulation step governed by the prior diffusion case and LSP parameters, rather than as a universal re-hardening treatment. For carburized or carbonitrided gear steels, the key question is not whether LSP necessarily produces a large hardness increase; it is whether it can introduce a stable CRS field, adjust the near-surface defect and phase state, and move crack initiation away from vulnerable surface sites without damaging the hardened case. In 20CrNiMo steel, this regulation is reflected in the reported crack-origin transition. Representative routes, strengthening mechanisms, main findings, and process-window characteristics are summarized in Table S6 [50,192,201,208].

### 6.2. Layered Synergistic Strengthening Through Mechanical Treatment and LSP

Mechanical pre-treatments such as shot peening (SP) alter the surface topography, near-surface plastic state, and shallow CRS distribution. A subsequent LSP step can drive the compressive stress field deeper into the material and reshape the subsurface stress profile [85]. Ultrasonic-assisted hybrid routes introduce high-frequency vibration into impact, rolling, or laser-shock loading, promoting surface plastic deformation, dislocation multiplication, grain refinement, and CRS formation [209,210]. Because direct evidence for these hybrid routes in gear steels is still limited, CRS-depth improvement in gear steels is used as the main basis, while results from other steels and light alloys are drawn on to discuss fatigue, wear, and crack-resistance responses.

In SP–LSP sequential treatment, SP first modifies the shallow plastic-deformation layer and near-surface CRS state, after which LSP extends the compressive stress field to greater depths. In 20Cr2Ni4 steel, increasing the laser-shock peak pressure from 3.0 to 4.5 GPa increased the CRS depth from 0.19 to 0.38 mm. When LSP was applied after SP pretreatment, a deeper CRS distribution was more readily obtained [193]. Results for SP + LSP-treated 30CrMnSiA steel further show that the hybrid route does not provide a uniform benefit at all stress levels. SP + LSP reduced surface roughness, increased the fatigue limit from 391.44 to 413.24 MPa, and produced a more uniform ultrafine-grained structure with higher dislocation density [85]. The benefit was mainly observed in the low-stress regime. Above 478 MPa, SP-treated samples showed longer fatigue lives, indicating that high-stress loading can reduce the contribution of deep CRS and refined microstructure through rapid crack propagation and surface-damage effects.

**Figure 24:**
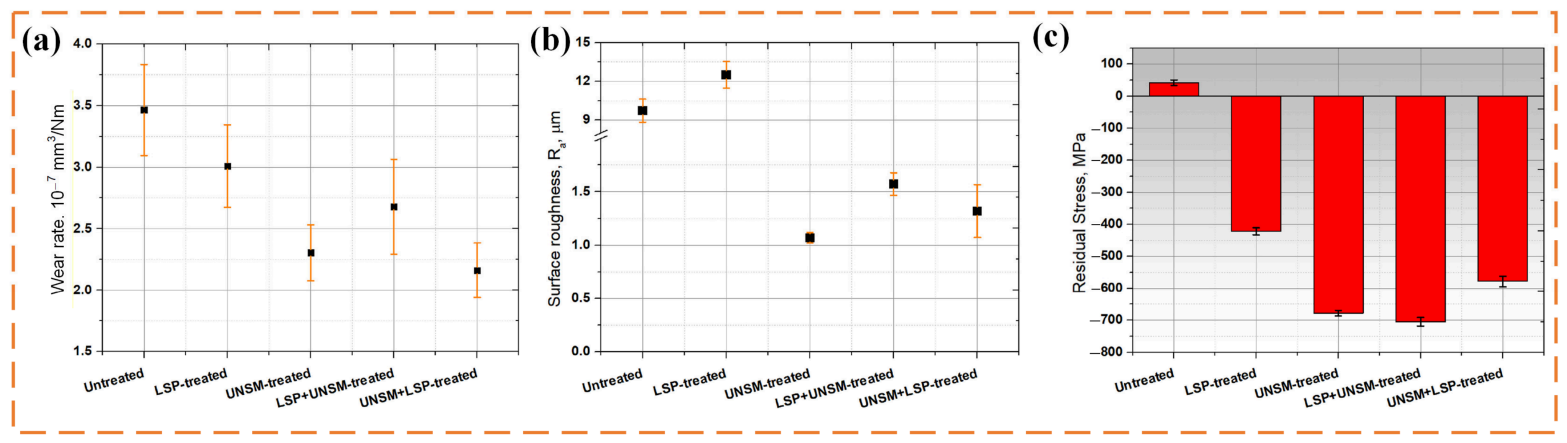
Sequence-dependent surface response after laser shock peening (LSP) and ultrasonic nanocrystal surface modification (UNSM) of SLM Ti–6Al–4V: (**a**) surface roughness; (**b**) surface residual stress; and (**c**) wear rate after LSP, UNSM, LSP + UNSM, and UNSM + LSP treatments. This non-steel alloy case is used only as an illustrative analogue for treatment-order effects. Panels are adapted from Ref. [190] under the terms of the Creative Commons Attribution License.

Ultrasonic-assisted LSP represents a vibration–shock coupled route, but directly reusable figure evidence is mainly available from non-steel alloy systems. In SLM Ti–6Al–4V, combined LSP and ultrasonic nanocrystal surface modification (UNSM) changed surface roughness, surface CRS, hardness, and wear response (Figure 24) [190]. LSP alone increased the roughness of the as-built surface, whereas UNSM reduced the roughness sharply; the combined LSP + UNSM and UNSM + LSP routes also produced much smoother surfaces than the untreated and LSP-only states. Residual-stress results showed that LSP, UNSM, and their combined routes all converted the near-surface tensile stress into CRS, with LSP + UNSM giving the highest surface CRS of about −705 MPa. In wear testing, LSP reduced the wear rate from 3.57 × 10^−8^ to 3.04 × 10^−8^ mm^3^ / (N m), while UNSM reduced it further to 2.06 × 10^−8^ mm^3^ / (N m); the combined routes also improved wear resistance compared with the untreated and LSP-only states.

Sequential SP–LSP treatment mainly uses shallow pre-deformation to support the development of a deeper CRS field, whereas ultrasonic-assisted routes rely on high-frequency vibration to promote surface plastic deformation, hardening, and CRS formation. Current evidence from gear steels mainly confirms CRS-layer deepening; the associated fatigue and wear responses still need to be verified under gear-steel bending fatigue, RCF, and tooth-flank wear conditions. Representative routes, strengthening mechanisms, key results, and process-window limitations are summarized in Table S7 [85,190,193,211].

### 6.3. Microstructure and Stress Regulation by LSP in Phase-Transformed and Precipitation-Strengthened Layers

Quenching, laser hardening, high-frequency induction quenching, and aging can produce hardened surface layers dominated by martensite, retained austenite, carbides, or precipitates [212,213]. When LSP is superimposed on these hardened layers, high-strain-rate plastic deformation further modifies the dislocation structure, grain size, retained-austenite stability, and CRS distribution. The additional strengthening is therefore governed by the coupled effects of microstructural refinement, phase-constitution adjustment, and stress-field regulation [214].

#### 6.3.1. LSP Response of Martensitic Hardened and Precipitation-Strengthened Layers

After carburizing, quenching, and tempering, the surface layer of gear steels typically contains martensite, retained austenite, carbides, and pre-existing residual stresses. Direct evidence for the coupled evolution of such heat-treated gear-steel surfaces under LSP is still limited. Hardened steels and precipitation-strengthened steels with comparable microstructural features therefore provide the basis for discussing LSP-induced changes in phase constitution, defect structure, and CRS response.

**Figure 25:**
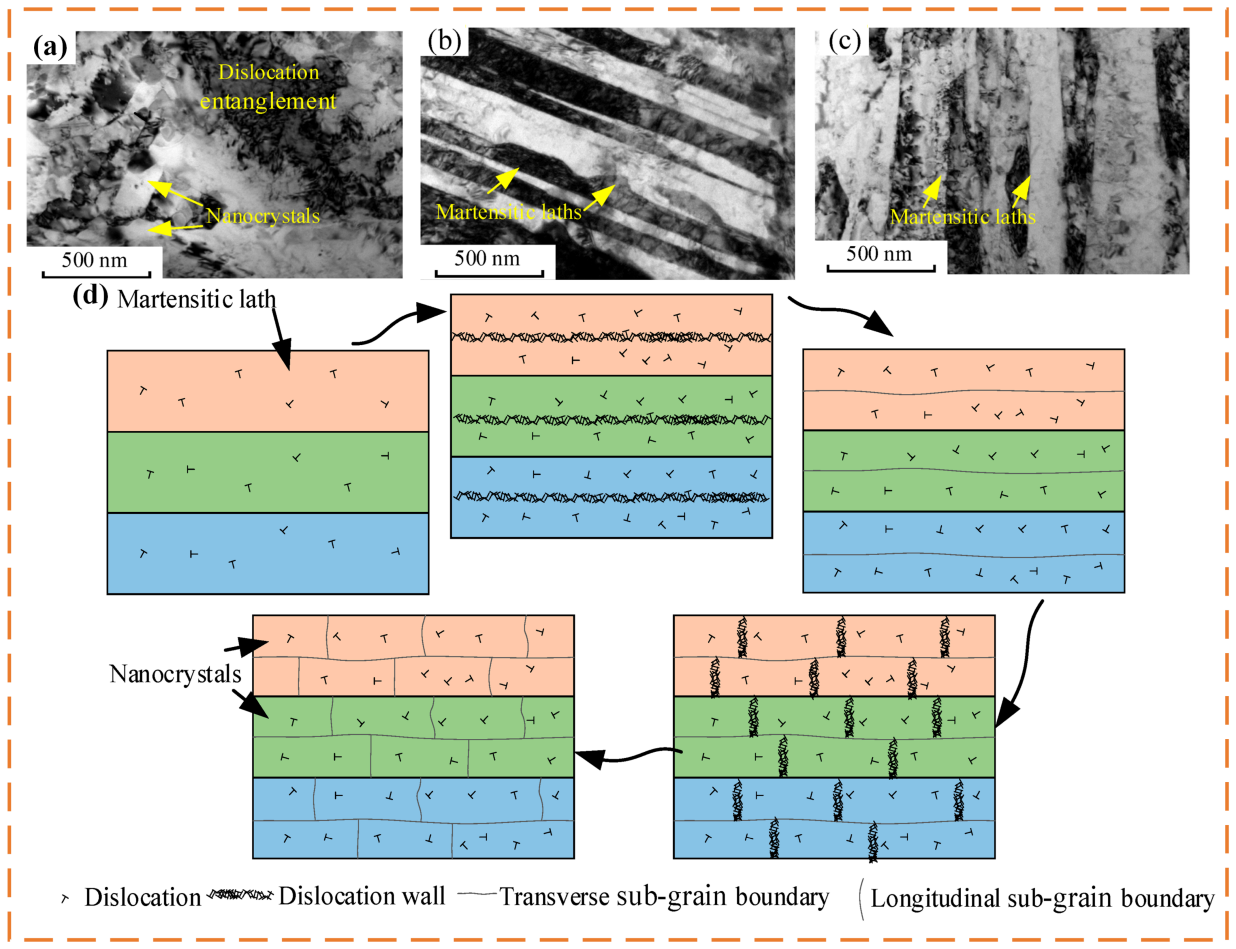
LSP response of a martensitic hardened layer in 14Cr12Ni3Mo2VN steel: TEM microstructures of the HFIQ–LSP-treated sample showing (**a**) nanocrystalline surface refinement, (**b**) short-lath martensite in the subsurface layer, and (**c**) coarse-lath martensite in the core; (**d**) schematic process of LSP-induced nanocrystal formation through dislocation accumulation, dislocation-wall formation, subgrain-boundary development, and surface-layer recrystallization. Panels are adapted from Ref. [216] under the terms of the Creative Commons Attribution License.

Martensitic hardened layers generally consist of a martensitic matrix, retained austenite, carbides, and a high density of lattice defects. The response to LSP is mainly reflected in phase transformation, carbide refinement, defect-structure evolution, and redistribution of the CRS field. Feng et al. combined laser hardening with LSP in Cr12MoV steel and reported that LSP promoted the transformation of retained austenite into martensite, refined the carbides, and improved the CRS distribution. The surface microhardness increased to 722.3 HV, the peak surface CRS reached −383.84 MPa, and the retained-austenite content decreased to 0.8% [215]. In 14Cr12Ni3Mo2VN martensitic stainless steel, high-frequency induction quenching (HFIQ) combined with LSP produced a layered martensitic gradient consisting of a nanocrystalline surface layer, a short-lath martensitic subsurface layer, and a coarse-lath martensitic core (Figure 25a–c) [216]. TEM observations showed nanocrystals and dislocation entanglement near the surface, martensitic laths with an average width of approximately 80 nm in the subsurface layer, and coarser martensitic laths of approximately 250 nm in the core. The proposed refinement route involves dislocation accumulation in martensitic laths, followed by dislocation tangling, dense dislocation-wall formation, subgrain-boundary development, and eventual nanocrystal formation in the highly strained surface layer (Figure 25d). The hybrid-treated sample also developed a hardness gradient, with the surface hardness reaching about 524 HV_0.1_, and a near-surface CRS field with a maximum magnitude of approximately −575 MPa at a depth of about 0.1 mm.

For martensitic hardened layers, LSP mainly promotes retained-austenite transformation, carbide refinement, martensitic-lath subdivision, nanocrystal formation, and CRS redistribution. The Cr12MoV and 14Cr12Ni3Mo2VN cases provide a mechanistic reference for interpreting the response of quenched, tempered, carburized, or otherwise martensitic surface layers in gear steels. Representative routes, strengthening mechanisms, and applicable conditions are summarized in Table S8 [215,216].

#### 6.3.2. Precipitation and Surface-Hardening Responses After LSP Pre-Deformation

Applying LSP before aging or surface hardening-existing deformation features can alter precipitate morphology, hardened-layer depth, and the hardness gradient developed during the subsequent thermal step. Direct evidence from gear steels remains limited, so age-hardenable alloys and surface-hardened steels are used here as mechanistic references.

**Figure 26:**
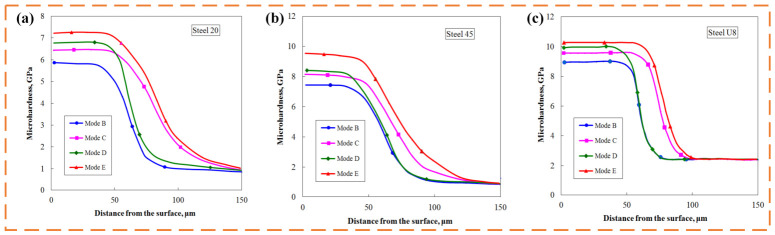
Effect of combined laser shock peening (LSP) and laser surface hardening (LSH) on hardened-layer depth and microhardness in Fe–C steels: depth-direction microhardness distributions of (**a**) 20 steel, (**b**) 45 steel, and (**c**) U8 steel after conventional LSH and different LSP/LSH sequences. Panels are adapted from Ref. [217] under the terms of the Creative Commons Attribution License.

In Alloy 625, LP before aging produced a deeper affected layer than SP and, after aging, extended the strengthened layer to approximately 3000 μm; the near-surface region contained finer and denser γ″ precipitates, whereas SP-treated samples were more prone to coarse acicular δ-phase formation [200]. This case is retained only as a precipitation-related analogue. A closer steel-based example is provided by Bragov et al., who compared different LSP and laser surface hardening (LSH) sequences in 20 steel, 45 steel, and U8 steel. Compared with conventional LSH, the LSP–LSH–LSP route produced the strongest hardening response (Figure 26) [217], hardened-layer depth increased by factors of 1.53, 1.41, and 1.29, respectively, while maximum microhardness increased by 22%, 27%, and 13%. LSP pre-deformation can modify later thermal strengthening, but for gear steels this route remains a mechanism-level reference rather than direct component evidence.

#### 6.3.3. Synergistic Boundaries and Non-Synergistic Responses

Thermo-mechanical hybrid treatments do not always produce additive benefits. In SLM-fabricated 17-4PH stainless steel, Kumar et al. compared AP, AP + LSP, AP + HT, and AP + HT + LSP conditions under high-temperature sliding wear [218]. The AP + LSP condition showed a more stable wear-resistance advantage, whereas the HT + LSP route provided limited synergy. As temperature increased, oxide films repeatedly formed, fractured, and spalled under shear, and the wear process gradually shifted toward oxidation-assisted delamination and three-body abrasion. Under this damage mode, CRS, work hardening, and microstructural refinement introduced by LSP could still reduce local damage, but oxide-film instability and progressive removal of the hardened layer continuously consumed their contribution. This case indicates that hybrid strengthening should be evaluated not only by post-treatment hardness and CRS, but also by stress retention, oxide-film stability, and wear-mechanism evolution during service.

### 6.4. Hybrid Regulation of the Load-Bearing Layer and Tribological Interface by LSP and Surface Texturing

Tooth-flank wear is controlled by both the load-bearing state of the near-surface layer and the response of the tribological interface. LSP strengthens the surface layer through hardening, CRS, and microstructural refinement, whereas surface texturing modifies real contact area, debris transport, and lubricant retention. Their combination is therefore useful only when the strengthened layer and the textured interface are matched to the lubrication regime and wear mechanism.

In 9Cr18 steel, LSP increased the surface hardness to 820 HV_0.1_, produced a maximum CRS of −876 MPa, and reduced the grain size by 10.20% [219]. Under boundary lubrication, the best response was obtained by combining 6 J LSP with a texture depth of 18.0 μm and a surface density of 37%; the coefficient of friction (COF) decreased from 0.114 to 0.035, and the wear loss decreased from 50.5 to 0.3 mg. Under dry sliding, the optimal texture depth shifted to 14.6 μm, while the surface density remained 37%, showing that texture geometry cannot be separated from the friction regime. A related YG8 cemented carbide study also showed that coating-free LSP combined with laser-ablation texturing increased hardness, reduced COF, and suppressed adhesive wear [220].

### 6.5. Summary

Hybrid strengthening is needed because LSP alone cannot satisfy all gear-surface requirements. Thermochemical treatment mainly builds the hardened case, whereas LSP extends compressive-stress support into deeper regions; finishing or texturing becomes necessary when roughness, defects, or lubrication behavior dominate. The benefit depends on treatment sequence and the prior surface state, and poor matching may cause stress relaxation, roughening, or case damage. For gears, route selection should follow the local failure risk, especially tooth-flank surface integrity, tooth-root CRS continuity, and strengthening uniformity in restricted bores.

## 7. Component-Level Bottlenecks and Process Development Directions for Gear LSP

The preceding analysis has shown how LSP modifies the near-surface state, surface integrity, and hybrid strengthening response of gear-related materials. For actual gear components, the unresolved issue is how to establish and retain a stable strengthened state in geometrically complex regions. Gear LSP must accommodate tooth-flank contact regions, tooth-root transitions, and near-bore regions while maintaining reproducible CRS fields, hardness gradients, and surface topographies under variations in tooth curvature, initial surface integrity, and batch-processing conditions [49,221]. The following discussion therefore focuses on adaptation to the initial material state, boundaries of strengthening benefit, strengthening in complex regions, process consistency, and manufacturing integration [41].

### 7.1. Influence of Initial Surface Integrity and Manufacturing Route on the LSP Strengthening Response

The growing use of additive manufacturing (AM), laser repair, and complex gear-blank manufacturing further increases the variability of the initial state. As-built defects, microstructural anisotropy, and intrinsic residual stresses affect both fatigue performance and the response to subsequent surface strengthening [222,223]. In laser powder bed fusion (LPBF) -built miniature 17-4 PH spur gears, LSP near the gear root introduced CRS to a depth of about 0.4 mm, improved surface roughness by approximately 32%, and reduced grain size by about 15.6% [102]. Studies on 316L stainless steel fabricated by different laser-based additive routes, including selective laser melting (SLM), laser deposition (LD), laser metal deposition (LMD), further show that build orientation, initial tensile stress, and component thickness influence microstructural evolution, CRS magnitude, and strengthened-layer depth after LSP [224,225,226,227].

### 7.2. Material-Level Surface-Integrity Tolerance and Mapping to Gear Damage Response

Material-level LSP studies have revealed a clear trade-off between strengthening gains and surface damage [228,229,230]. In LPBF 316L stainless steel, repeated impacts continued to increase CRS, hardness, and corrosion resistance; once roughness and local damage accumulated, however, the wear response no longer improved [231]. In LPBF ultra-high-strength low-alloy steel, a moderate impact input increased the fatigue limit from 467 to 720 MPa. Further increasing the input aggravated surface pits and roughening, and the fatigue performance fell below that of the substrate [232]. Cold-sprayed AM 316L coatings showed a different response: when pore closure and surface densification dominated, higher hardness and lower frictional wear could be achieved simultaneously [233].

The evidence points to a finite energy-input window for LSP. Additional shock input is useful only while CRS deepening and hardening outweigh the penalty from roughening, pits, and local damage; beyond that point, a higher CRS level or a thicker hardened layer may no longer translate into better gear-relevant performance. Because most data still come from material specimens, the mapping from surface-integrity descriptors to gear damage under rolling–sliding and bending loads remains insufficiently resolved. Simple regression is unlikely to describe such a coupled response. Data-driven models, including neural networks, are better used at this stage to connect measured strengthening tolerances with candidate process windows for gears, rather than as stand-alone predictors of service performance.

### 7.3. Laser Access and Continuous Strengthening in Constrained Inner-Bore Regions

**Figure 27:**
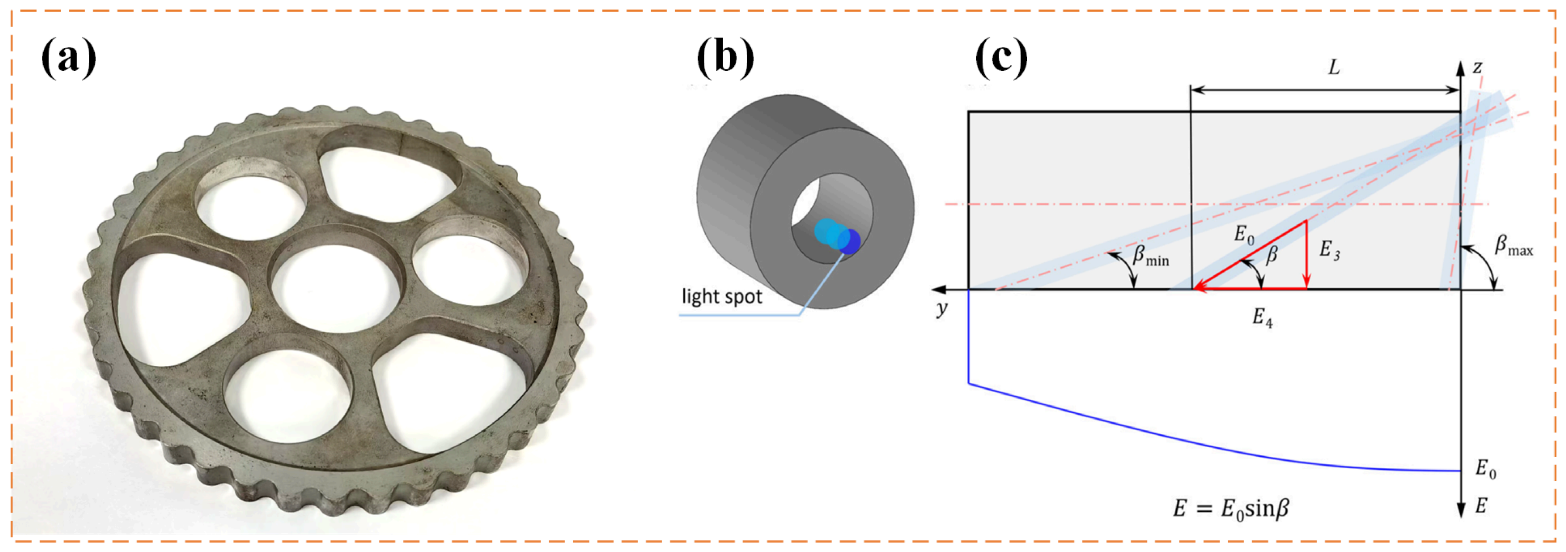
(**a**) Cycloidal gear of an RV reducer; (**b**) schematic of the axial laser spot and (**c**) axial decomposition of OLSP for the guide hole. The blue shaded regions represent the obliquely incident laser beams, the red dash-dotted lines indicate the corresponding beam centerlines, and the blue curve schematically represents the variation in the effective normal energy component according to $E=E_{0}\sin\beta$; the remaining symbols are defined in the main text and equations. Panel (**b**,**c**) is reproduced from Ref. [234] under the terms of the Creative Commons Attribution license.

Exposed regions such as tooth flanks and tooth roots generally provide good laser accessibility, and continuous strengthening coverage can be obtained by adjusting the scanning path, spot size, and overlap ratio. Constrained regions, including bore walls, internal cavities, and deep holes, are restricted by line-of-sight laser delivery and wall shielding, making sustained normal incidence difficult. Laser processing inside a bore usually requires oblique incidence, which reduces the normal component of the shock load and leads to nonuniform strengthening along the bore depth. The crankshaft bore in the cycloidal gear of an RV reducer is a typical constrained load-bearing region. The bore wall is subjected to local contact and support constraints while also being limited by laser-access space, so stable coverage is more difficult to achieve than on exposed tooth flanks (Figure 27a).

As shown in Figure 27b, Liu et al. proposed a nitriding–oblique laser shock peening process (N-OLSP) for inner bore walls and introduced a uniformity coefficient k, to describe the normal impact component of an oblique laser spot on the bore wall. The coefficients $k_{x}$ and $k_{y}$ correspond to the circumferential and bore-depth directions, respectively. In axial strengthening, $k_{y}$ is related to the laser deflection angle β. A smaller β allows the beam to reach deeper positions along the bore wall, but it also reduces the normal impact component and weakens the axial strengthening uniformity. Under the reported bore-diameter and spot-size conditions, $k_{y}$ decreased to approximately 0.5 at $\beta \approx 30^{\circ }$, corresponding to an effective strengthening depth of about 15.8 mm along the bore. Further extension into the bore provided limited additional strengthening. After the hybrid treatment, only slight friction marks were observed on the inner bore wall, and the maximum wear rate near the bore mouth decreased to 0.60 μm/h, representing reductions of 66.85% and 16.67% compared with the untreated and nitrided-only samples, respectively [234].

A smaller β under oblique incidence also elongates the beam footprint and increases the projected area. When the single-pulse energy remains unchanged, the pulse energy per unit area decreases, reducing the CRS magnitude and affected depth. Energy-density compensation can therefore improve the strengthening effect at smaller β values [235].

These results can be used to develop an energy-density compensation model for LSP of constrained bore walls. Bore diameter, bore depth, beam diameter, and laser deflection angle β can be used as geometric inputs to calculate oblique-spot coverage, $k_{y}$ attenuation, and the effective strengthening length along the bore depth. A footprint correction for oblique incidence can then be introduced to convert the pulse energy per unit area and relate it to CRS magnitude and affected depth. For load-bearing inner bores such as crankshaft bores, the required energy compensation at different β values and bore-depth positions can be calculated with this model to reduce CRS-field fluctuations during continuous oblique peening.

### 7.4. Process Sensing and Control of Strengthening Consistency

The LSP response is highly transient and nonlinear. Residual stress, hardness, and surface topography are jointly affected by shock input, confinement-layer stability, material elastoplasticity, and the initial surface condition [236,237]. Gear components introduce additional variations in curvature, incidence angle, and overlap trajectory. The same nominal parameter set can therefore produce different strengthening responses on the tooth flank, at the tooth root, near bore openings, and along bore walls. Process sensing should accordingly move beyond single-point prediction of residual stress or hardness and toward the identification of local process deviations, including under-peening, over-peening, excessive roughening, and insufficient affected depth [238,239].

**Figure 28:**
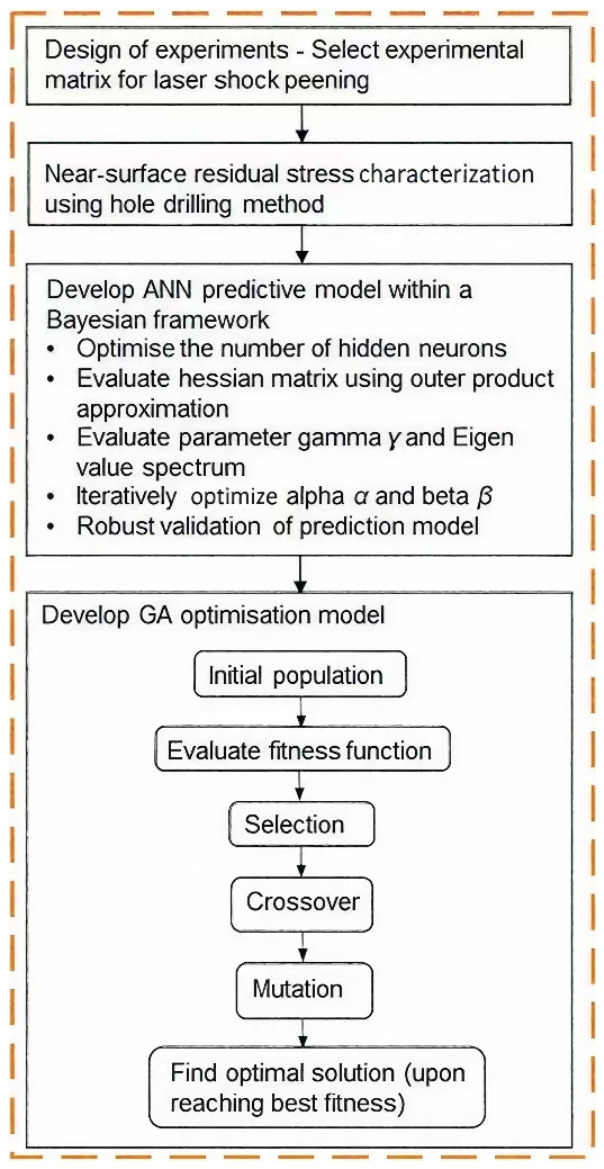
Machine-learning-based prediction and optimization framework for LSP-induced residual stress: ANN model for forward prediction of residual-stress distribution and genetic algorithm for reverse optimization of process parameters. Panel is adapted from Ref. [242] under the terms of the Creative Commons Attribution License.

Acoustic emission (AE) spectra can capture changes in plastic deformation and grain refinement caused by variations in impact energy, and can also distinguish material states with different hardness levels [240]. Spectral–acoustic fusion further connects optical spectral fields and acoustic fields with surface-integrity metrics such as residual stress and microhardness, improving both diagnostic accuracy and robustness [241]. Machine-learning prediction and optimisation frameworks provide another route for process-consistency control. Mathew et al. developed a Bayesian ANN model to predict LSP-induced residual-stress distributions from process parameters and coupled it with a genetic algorithm for reverse optimization of the peening parameters required to reach a target residual-stress state (Figure 28) [242].

Region-dependent geometry and boundary conditions further complicate the signal–response relationship in gear components. AE, optical spectral, and acoustic features therefore combine information from shock input with effects associated with local curvature, incidence condition, confinement-layer stability, and initial topography. Similar spectral changes may arise from fluctuations in impact input, but they may also reflect differences in local curvature, incidence condition, or surface state. A clear correspondence between these signal features and local deviations such as under-peening, over-peening, or excessive roughening has not yet been established. Before such correlations are available, process sensing mainly supports specimen-level prediction of residual stress and hardness, rather than direct control of strengthening consistency from tooth to tooth. Future work should establish region-specific links among process signals, CRS depth, hardness gradients, and topographical changes in representative tooth-flank, tooth-root, and bore-wall regions, and use these links to refine local process windows.

Recent work has begun to make this step less dependent on repeated finite element method (FEM) runs. Sun et al. used an FEM-augmented attention-enhanced graph convolutional generative adversarial network (AGCGAN) to reconstruct laser shock peening (LSP) residual-stress fields in GCr15 bearing steel, reaching 95.98% accuracy and a 445-fold speed gain [243]. Barile et al. are useful in another sense: random forest–SHapley Additive exPlanations (RF–SHAP) analysis of thick aluminum alloy (AA) 7050 specimens made parameter interactions and over-peening more explicit [244]. Gear use still needs local calibration near roots, flanks, tooth gaps, and bores.

### 7.5. Manufacturing-Cell Integration and Pulse-Energy Utilization

Clauer et al. framed the engineering implementation of LSP around the development of compact manufacturing cells. By integrating an enclosed processing space, a workpiece-motion platform, and a confinement-medium delivery system, laser shocking, workpiece motion, and medium supply can be coordinated as a continuous operation rather than treated as separate steps [245]. This form of cell-based integration moves LSP from discrete laboratory processing toward a manufacturing-compatible module, providing a more stable basis for repeatable component-level treatment.

**Figure 29:**
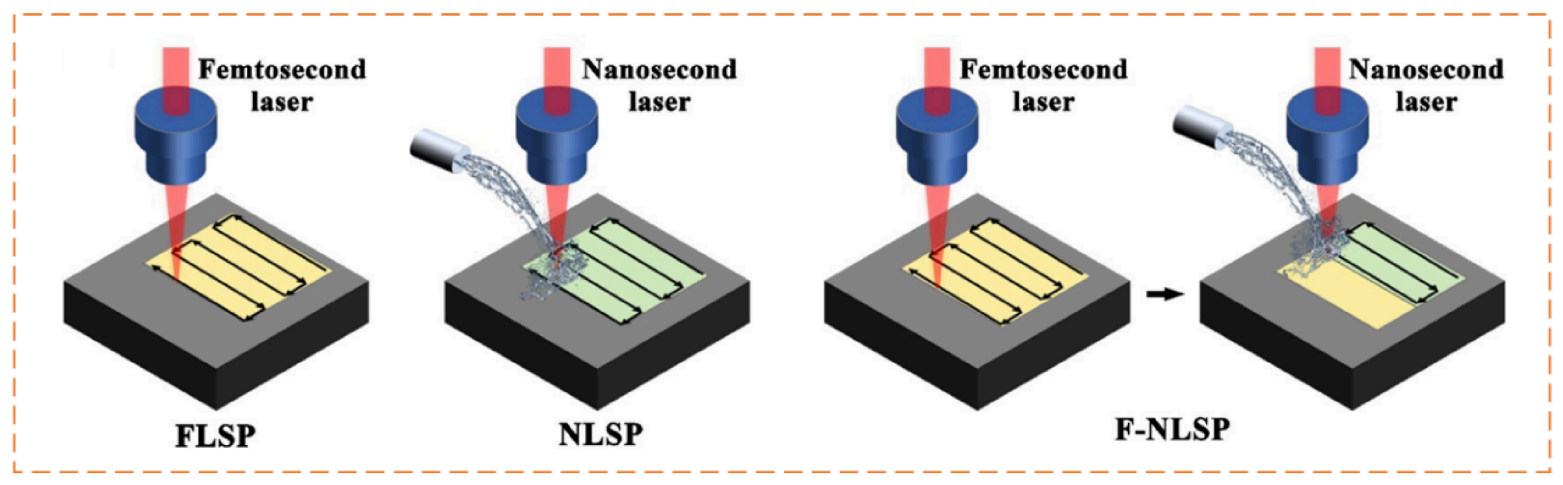
Schematic comparison of FLSP, NLSP, and combined F-NLSP processing routes. The black arrows on the specimen surfaces indicate the laser scanning paths, the yellow and green regions represent the areas treated by FLSP and NLSP, respectively, the water-spray symbol denotes the confining water layer used during NLSP, and the arrow between the two F-NLSP schematics indicates sequential FLSP followed by NLSP.Panels are adapted from Ref. [246] under the terms of the Creative Commons Attribution License.

Pulse-sequence design offers another route to improving energy utilization during LSP. In combined femtosecond-nanosecond LSP of Ti6Al4V alloy, femtosecond pulses first modified the treated surface and improved optical absorption, after which nanosecond LSP produced a stronger in-depth hardening response than nanosecond LSP alone (Figure 29) [246]. Compared with nanosecond laser shock peening (NLSP), the combined laser shock peening with combined femtosecond and nanosecond laser pulses (F-NLSP)route produced denser refined grains and a larger affected depth, together with a higher in-depth nanohardness amplitude. This result indicates that pulse sequencing can improve laser–material coupling and increase the strengthening response obtained from subsequent shock loading, although its transfer to gear steels still requires validation. Studies on temporal pulse-shape control further show that, even in nanosecond LSP without a confinement layer, CRS can be enhanced through pulse-time design [247]. Manufacturing-cell integration therefore addresses continuous operation, whereas pulse-sequence control improves the plastic response obtained per unit energy input.

### 7.6. Coupling with Manufacturing Processes and Earlier Introduction of Shock Treatment

**Figure 30:**
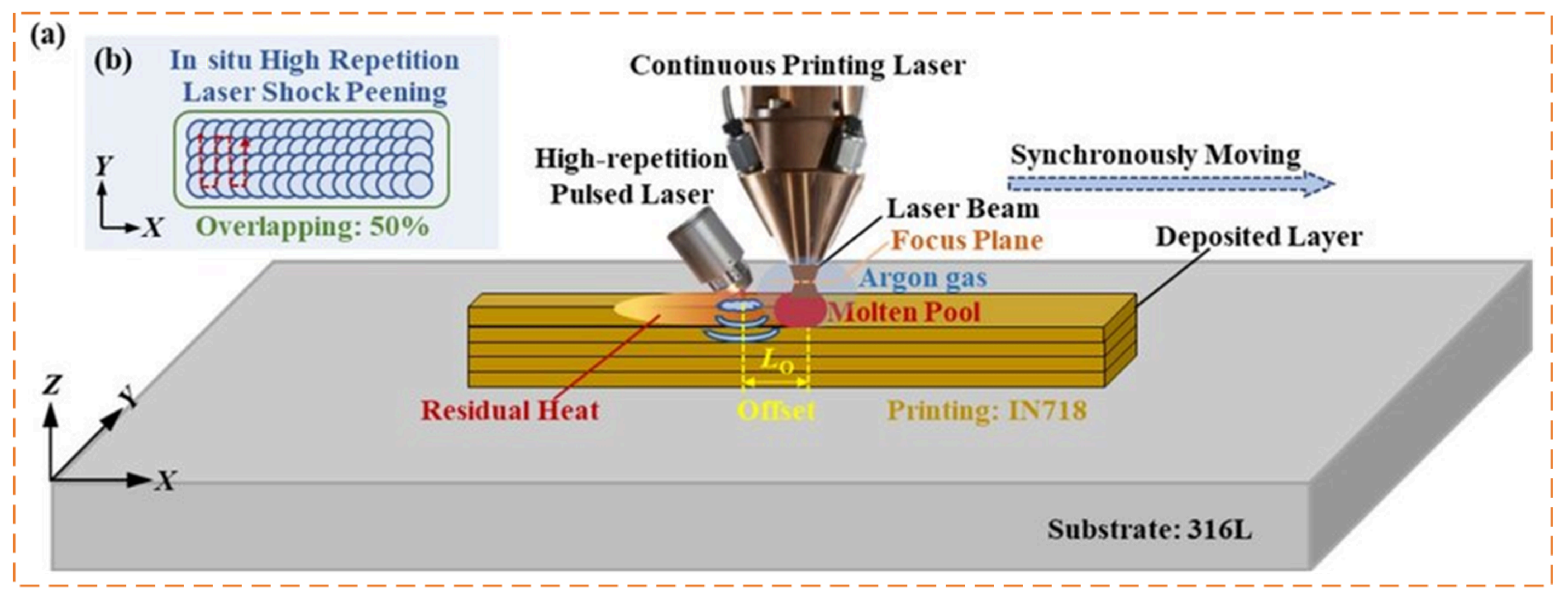
HRLSP during LDED of IN718 alloy: (**a**) schematic illustration of the coupled LDED–HRLSP process, in which the shock source moves with the deposition head and acts on the recently solidified region; (**b**) HRLSP path with 50% overlap. Panels are adapted from Ref. [248] under the terms of the Creative Commons Attribution License.

Synchronized high-repetition laser shock peening (HRLSP) during laser-based directed energy deposition (LDED) provides a clear example of this shift (Figure 30) [248]. In this route, the HRLSP source moves synchronously with the deposition head and acts on the recently solidified region behind the molten pool (Figure 30a). The peening path is arranged with overlapping impacts, allowing shock-induced plastic deformation and residual-stress regulation to be introduced during layer accumulation rather than only after final forming (Figure 30b). For AM gears and complex gear blanks, this strategy is relevant when the target is interlayer porosity, tensile residual stress, or a deeper affected layer. Its transfer to gear manufacturing remains conditional: the added shock step must not introduce tooth-profile error, excessive roughening, or dimensional deviation beyond the correction capacity of subsequent machining.

### 7.7. Hybrid Routes Should Be Selected as Process Chains

For gears, the near-term route is still a diffusion case followed by local LSP. Carburizing or carbonitriding provides the load-bearing case, while LSP adjusts residual stress, microstructure, and crack-origin behavior [50]. In 20CrNiMo steel, carburizing–LSP improved bending fatigue but showed a limit at excessive impacts [192]. Similar gradient superposition was reported in carburized bearing steel [201]. Pre-LSP can also refine the surface layer before plasma nitriding [206]. LSP–nitriding coupling has been reported for precipitation-hardened stainless steel [207].

Coating-assisted routes are less mature for precision gears. Current LSP studies often treat coatings as protective or sacrificial layers [78]. For functional coatings, LSP may improve densification and adhesion after suitable preconditioning, but may also cause delamination under unfavorable interface conditions [249]. Laser-shock micro-textured substrates with TiN coatings further indicate that interface adhesion should be evaluated beyond hardness [250].

AM + LSP and LSP + laser texturing are more selective routes. For printed or repaired gear blanks, LSP mainly targets tensile-stress reduction, defect mitigation, and compatibility with later machining [102]. For textured flanks, lubricant retention must be balanced against CRS depth, hardened-layer support, and contact pressure, because unsuitable textures may become crack-initiation sites [219,251].

### 7.8. Summary

LSP in gear-related materials and local gear surfaces is constrained by the way shock loading is delivered to, and retained within, the damage-prone surface-to-subsurface region. Prior heat treatment, grinding or polishing, residual stress inherited from earlier processing, AM defects, and geometric features such as fillets, bore walls, and curved cavities can all shift the resulting CRS profile, hardening response, and surface topography. Tooth flanks leave little tolerance for roughening because of rolling–sliding contact and lubrication requirements, whereas the tooth-root fillet benefits mainly from a continuous CRS field that slows crack growth. In bore walls and other confined regions, oblique incidence and wall shielding further reduce the normal shock component and make strengthening less uniform. Process sensing, compact processing cells, pulse control, and high-repetition-rate laser shock peening (HRLSP)during laser directed energy deposition (LDED) may help reduce this variability, but their significance for gear-related applications will be measured by a simpler criterion: whether a stable strengthened layer can be obtained without losing surface integrity or dimensional accuracy.

## 8. Conclusions and Perspectives

### 8.1. Conclusions

LSP can generate CRS fields, hardness gradients, and refined microstructures in the near-surface region of gear steels and related metallic materials, with corresponding improvements in fatigue, wear, and crack-growth behavior. The main conclusions drawn from this review are as follows:

1. The strengthening response of LSP is controlled by laser energy, pulse duration, spot size, scanning path, overlap ratio, and number of impacts. For gear applications, the process window must accommodate tooth-flank topography control, continuous CRS coverage at the tooth root, and stable beam access and interfacial confinement in constrained regions such as inner bores, tooth gaps, and curved cavity surfaces.
2. The primary near-surface responses induced by LSP are CRS fields, hardness gradients, plastically deformed zones, and gradient microstructures. For gear damage-critical layers, CRS depth, peak position, and spatial uniformity are more informative than the residual-stress peak alone in assessing strengthening quality.
3. The functional requirements of LSP differ between the tooth flank and the tooth root. Tooth-flank contact regions are more strongly governed by surface topography, lubrication state, and subsurface contact stress, whereas tooth-root transition regions require a deep and continuous CRS field, stable compressive-stress retention, and crack-growth retardation.
4. Within a suitable parameter range, LSP can increase the bending-fatigue limit, extend RCF life, reduce crack-growth rate, and mitigate wear damage. Excessive energy input or repeated impacts, however, can cause roughening, micropitting, and local ablation, while the additional CRS gain approaches saturation.
5. Stand-alone LSP cannot fully satisfy the multilevel service requirements of gears from the surface to the subsurface region. When coupled with carburizing, nitriding, shot peening, rolling, surface texturing, or coatings, LSP can complement surface load-bearing support, subsurface stress regulation, and friction reduction at the contact interface.

### 8.2. Perspectives

The unresolved issue is mainly one of translation. Results from flat specimens, simplified steel samples, and isolated parameter studies still need to be tied to gear geometry, retained surface integrity after service-relevant loading, and fatigue–wear response. The following points identify where this translation remains incomplete.

1. Initial surface integrity and manufacturing route need clearer reporting before peening. Prior heat treatment, finishing marks, inherited residual stress, AM defects, build orientation, and section thickness can change CRS development, hardening response, and microstructural refinement [100,102,219].
2. The surface-integrity tolerance of LSP has to be related to gear damage response. A higher CRS level or deeper hardened layer may bring little benefit if roughening, pits, or local depressions disturb contact and lubrication [119]. Data-driven methods may help translate material-level tolerance limits into gear-oriented process criteria [123,124].
3. Constrained inner bores require compensation based on laser access and bore geometry. Bore-wall shielding and oblique incidence reduce the normal shock component and can make CRS nonuniform along the bore depth. Laser deflection angle, bore-depth uniformity coefficient, effective strengthening length, footprint correction, and energy-density compensation should therefore enter bore-wall process design [228,229,230].
4. Process sensing needs closer links with local strengthening states. AE, optical spectral, and acoustic features should be calibrated against CRS depth, hardness gradients, surface-topography change, under-peening, and over-peening in tooth-flank, tooth-root, and bore-wall regions, rather than used only for residual-stress or hardness prediction [231,237].
5. Manufacturing-cell integration, repeatability, and cost control remain open for gear steels. For large-scale gear production, the issue is not simply generating a deep CRS layer, but doing so repeatedly on different teeth and batches without drifting from the required surface-integrity state. Aircraft-engine blades and blisks offer a useful industrial reference: their LSP cells rely on faster lasers, coating/removal automation, robot motion, diagnostics, and stable overlays, but the geometry and production rhythm of gears are different [29]. In gears, cycle time is quickly consumed by indexing, partial coverage, overlap control, and water or coating recovery. The tool path is also sensitive to small errors; a slight change in spot location, incidence, or stand-off can affect coverage near flanks, roots, gaps, and bore edges. Repeatability, in practice, has to be tied to recorded pulse energy, spot trajectory, confinement state, surface preparation, and post-peening topography. This is not only a quality-control issue, because curved M50 steel specimens already show geometry-dependent changes in CRS depth, roughness, hardened-layer depth, and wear response [136]. Coating-free LSP [41], F-NLSP [42], temporal pulse shaping, and newer laser systems may improve coupling efficiency and manufacturing flexibility, but their value for gears still has to be judged against cycle time, profile accuracy [239], contact-fatigue performance, and post-finishing layer retention [240].
6. Earlier shock treatment during manufacturing must be examined together with later finishing. HRLSP during LDED offers a route for introducing plastic deformation and residual-stress regulation during layer accumulation, but its use in AM gear blanks depends on whether machining or grinding can preserve the strengthened layer while controlling roughness, profile accuracy, and dimensional deviation [241].

Future studies should report material state, local geometry, laser-access condition, process signals, surface integrity, and failure response within the same experimental framework. Only with this information can separate gains in CRS, hardness, or fatigue life be translated into gear-specific process windows, rather than remaining isolated material-level indicators.

## Data Availability

No new data were created or analyzed in this study. Data sharing is not applicable to this article.

## Supplementary Materials

The following supporting information can be downloaded at: https://www.mdpi.com/article/10.3390/ma19153257/s1, Table S1. Effects of LSP on crack-initiation depth and damage path in gears and related materials. Table S2. Bending fatigue and crack-growth responses of gear steels and related high-strength steels after LSP. Table S3. Depth-dependent roles and applicability limits of individual strengthening routes and LSP. Table S4. Quantitative comparison of representative LSP studies on gear steels, gear components, and related load-bearing steels. Table S5. Typical hybrid routes, dominant mechanisms, representative results, and limitations of pre-LSP-assisted diffusion enhancement and case-layer formation. Table S6. Reconstruction routes and strengthening responses of pre-existing diffusion cases after post-LSP. Table S7. Representative routes, strengthening mechanisms, and main results/limitations of synergistic strengthening by mechanical pre-deformation and LSP. Table S8. Representative routes, strengthening mechanisms, and main results/limitations of LSP-based re-regulation after heat treatment.

## Abbreviations

The following abbreviations are used in this manuscript:

LSP	Laser Shock Peening
LSPwC	Laser Shock Peening without Coating
DPLP	Double-Pulse Laser Peening
LLSP	Layer-by-Layer Laser Shock Peening
LSF	Laser Shock Forging
AM	Additive Manufacturing
SLM	Selective Laser Melting
LPBF	Laser Powder Bed Fusion
LMD	Laser Metal Deposition
LD	Laser Deposition
LSH	Laser Surface Hardening
SP	Shot Peening
USRP	Ultrasonic Surface Rolling Process
CT	Carburizing
VCN	Vacuum Carbonitriding
QT	Quench and Temper
HFIQ	High-Frequency Induction Quenching
CRS	Compressive Residual Stress
TRS	Tensile Residual Stress
RCF	Rolling Contact Fatigue
PEEQ	Equivalent Plastic Strain
KAM	Kernel Average Misorientation
LAGB	Low-Angle Grain Boundary
HAGB	High-Angle Grain Boundary
FCG	Fatigue Crack Growth
COF	Coefficient of Friction
S_a_	Surface Arithmetic Mean Roughness
R_a_	Average Roughness
S_pk_	Reduced Peak Height
S_vk_	Reduced Valley Depth
S_sk_	Skewness
S_ku_	Kurtosis
*η*	Overlap Ratio
*β*	Laser Deflection Angle
*k*	Uniformity Coefficient
ANN	Artificial Neural Network
AE	Acoustic Emission
