# Supplementary material for: Laser Shock Peening of Gear Steels and Related Metallic Materials: Near-Surface States, Surface Integrity, and Component-Level Applications
Source: Materials (Basel). 2026 Aug 1;19(15):3257. doi: 10.3390/ma19153257 (PMC13467181; doi:10.3390/ma19153257)
Supplement: Supplementary file 1 [file materials-19-03257-s001.zip › materials-4439741-supplementary.pdf]

materials

Supplementary

Laser Shock Peening of Gear Steels and Related Metallic Materials: Near-Surface States, Surface Integrity, and Component-Level Applications

Yuxuan Sheng [1,2], Xin Hou [3,4], Yi Hou [1,2], Wenjie Chen [3,4], Qianjin Liu [3,4], Xiaoqiang Li [1,2] and Shengguan Qu [1,2,*]

[1] School of Mechanical and Automotive Engineering, South China University of Technology, Guangzhou 510640, China
[2] Guangdong Provincial Key Laboratory for Processing and Forming of Advanced Metallic Materials, National Engineering Research Center of Near-Net-Shape Forming for Metallic Materials, South China University of Technology, Guangzhou 510640, China
[3] KUKA Robotics Automation (Guangdong) Co., Ltd., Foshan 528300, China
[4] State Key Laboratory of High-End Heavy-Load Robots, Midea Group, Foshan 528300, China
* Correspondence: qusg@scut.edu.cn

Supporting Information Abstract

This supporting information file presents seven tables extracted from the main manuscript to streamline the layout and enhance readability. The tables provide detailed data and analyses related to laser shock peening (LSP) in gear steels and related high-strength materials:

- Table S1. Effects of LSP on crack-initiation depth and damage path in gears and related materials.
- Table S2. Bending fatigue and crack-growth responses of gear steels and related high-strength steels after LSP.
- Table S3. Depth-dependent roles and applicability limits of individual strengthening routes and LSP.
- Table S4. Quantitative comparison of representative LSP studies on gear steels, gear components, and related load-bearing steels.
- Table S5. Typical hybrid routes, dominant mechanisms, representative results, and limitations of pre-LSP-assisted diffusion enhancement and case-layer formation.
- Table S6. Reconstruction routes and strengthening responses of pre-existing diffusion cases after post-LSP.
- Table S7. Representative routes, strengthening mechanisms, and main results/limitations of synergistic strengthening by mechanical pre-deformation and LSP.
- Table S8. Representative routes, strengthening mechanisms, and main results/limitations of LSP-based re-regulation after heat treatment.

Table S1. Effects of LSP on crack-initiation depth and damage path in gears and related materials.

Material relevance	Interpretive focus	Material system	Key findings	Implications for gear failure analysis	Ref.
Gear-related material	Shift in crack-initiation depth	Carburized 20CrNiMo gear steel	After hybrid strengthening, the crack-initiation depth increased, and the surface was no longer the most failure-prone region.	Surface crack-initiation sensitivity was reduced, and the dominant crack-initiation depth shifted toward the subsurface region.	[1]

 https://doi.org/10.3390/ma19153257

Gear-related material	Shift in crack-initiation depth	AISI 9310 steel	Cracks initiated more frequently in the subsurface region, while surface crack initiation was suppressed.	The optimized residual stress field increased the threshold for surface crack initiation.	[2]
Related steel	Shift in crack-initiation depth	SS 304	Surface microcracks were reduced, and the damage location shifted inward.	Surface crack initiation was suppressed, with a shift in crack-initiation depth.	[3]
Related steel	Damage morphology and processing route	20Cr13 martensitic stainless steel	Increasing the number of coverage passes delayed crack initiation and changed the crack-propagation path.	Multi-step impact routes can regulate the surface microstructure and crack behavior.	[4]
Mechanistic reference	Improved defect tolerance	A7075-T651 aluminum alloy	Larger surface crack-like defects became tolerable after treatment.	Stress concentration at surface defect tips was reduced, leading to improved defect tolerance.	[5]
Mechanistic reference	Damage morphology and process window	Aluminum alloy	Higher power density enhanced CRS but aggravated surface roughening.	A processing trade-off exists between strengthening effect and surface integrity; both need to be balanced.	[6]
Mechanistic reference	Damage morphology and process window	AlZnMgCu1.5 aluminum alloy	Changes in processing parameters were accompanied by changes in roughness and residual stress state.	Damage morphology is governed by the process window.	[7]

Table S2. Bending fatigue and crack-growth responses of gear steels and related high-strength steels after LSP.

Material	LSP treatment route	Fatigue test type	Surface-integrity change	Fatigue and crack response	Ref.
54SiCr6	Strain-Assisted Tempering (SAT) SAT + LSP	Three-point bending fatigue	A compressive surface layer was introduced, and surface origin cracking was suppressed.	The S–N curve shifted upward; the mean fatigue limit increased from 837 to 980 MPa, with fatigue origins moving toward subsurface or internal defects.	[8]
AISI 9310	LSPwC followed by vibratory polishing	Bending fatigue	Ablation and remelting features were reduced, and a hardened layer of approximately 570 μm and a CRS layer of approximately 510 μm were formed.	The bending fatigue limit increased from 379.3 MPa to 598.6 MPa after LSPwC, and further to 705.7 MPa after vibratory polishing.	[9]
42CrMo	surface LSP; combined surface and side LSP	High-cycle fatigue	CRS coverage was introduced on the side surface.	Combined surface and side peening produced a longer fatigue life than surface peening alone, and crack initiation at edge corners was delayed.	[10]
30CrMnSiNi2A	LSP	High-cycle fatigue	A gradient CRS layer formed in the surface region.	The crack origin shifted from the surface to the interior.	[11]
42CrMo4	LSPwC	Cyclic loading/fatigue	A gradient structure formed in the near-surface region, consisting of a heat-affected layer, a severely plastically deformed layer, and the substrate.	Oxides, dislocation structures, and martensitic-lath degradation in the SPD layer were suppressed.	[12]

JIS SUS316L austenitic stainless steel	LSP	*K* decreasing fatigue crack growth test	The ΔK–da/dN curve shifted to higher ΔK values; ΔK_{th} increased from 3.71 ± 0.11 to 6.33 ± 0.20 Mpa $m^{1/2}$.	Laser peening increased near threshold crack-growth resistance, indicating a reduced effective crack tip driving force.	[13]
25Cr2Ni4MoV alloy steel	LSP	Fatigue test	The surface residual stress state was improved.	Fatigue striation spacing decreased from 1.34 μm to 1.03 μm, indicating a reduced crack-growth rate.	[14]

Table S3. Depth-dependent roles and applicability limits of individual strengthening routes and LSP.

Method category	Main role	Affected region	Applicability limits	Method category	Main role	Ref.
Shot peening	Surface plastic deformation, CRS, and work hardening; mainly controls surface crack initiation	Surface and near-surface layer	Limited CRS depth; roughening and dimples may require polishing for tooth flanks	Shot peening	Surface plastic deformation, CRS, and work hardening; mainly controls surface crack initiation	[15, 16, 17, 18,19, 20]
UNSM/USRP/ SMAT	Near-surface hardening and microstructural refinement	Surface and near-surface layer	Tool access limits use in tooth gaps, small modules, root fillets, and inner features; SMAT may require finishing	ultrasonic nanocrystal surface modification (UNSM), ultrasonic surface rolling process (USRP), and surface mechanical attrition treatment (SMAT)	Near-surface hardening and microstructural refinement	[21, 22, 23, 24, 25]
Deep rolling/burnishing	CRS combined with surface smoothing	Surface to moderate subsurface depth in accessible regions	Restricted by tool path and possible change in flank geometry	Deep rolling/burnishing	CRS combined with surface smoothing	[26-28]
Carburizing/nitriding	Diffusion case and hardness-gradient formation	Diffusion case and near-surface load-bearing layer	Deep CRS cannot be independently tailored; distortion, retained austenite, compound-layer brittleness, and finishing need control	Carburizing/nitriding	Diffusion case and hardness-gradient formation	[29-31]

https://doi.org/10.3390/ma19153257

Table S4. Quantitative comparison of representative LSP studies on gear steels, gear components, and related load-bearing steels.

Evidence focus	Material/evidence	Processing route and key parameters	CRS/hardening response	Performance response	Roughness/topography response	Main limitation	Ref.
Fatigue/RCF improvement	AISI 9310 carburized spur gear; G	Tooth-root LSP; E = 7/9 J, τ = 16 ns, d = 3 mm, η = 50%	Surface CRS near −700 MPa at 7 J; higher CRS magnitude and depth at 9 J	Single-tooth bending fatigue limit: 543.67 → 588.996/597 MPa, ↑8.3–9.8%	NR	Actual gear tooth-root fatigue evidence; hardness, roughness, and RCF were not reported	[32]
Fatigue/RCF improvement	20CrNiMo steel; GS	LSP uniformity + FEM; E = 4–6 J, τ = 14–26 ns, d = 3–5 mm, η = 30–62.5%	Optimal LSP-9: E_s = 45.69, Ep = 0.08; parameter influence: $\eta > \tau > E > d$	Bending fatigue life: 1.07 × 10^5 → 5.77 × 10^5 cycles, up to ↑5.41×	$R_a/S_a/S_q$: NR; surface deformation evaluated by plastic-strain uniformity	Hardness, microstructural refinement, roughness, RCF, and wear were not reported	[33]
Fatigue/RCF improvement	AISI 9310 steel roller; GS	Pre-LSP + RCF; E = 4 J, τ = 20 ns, d = 2.2 mm, η = 50%; p_{max} = 3200 MPa	Hardness: 236.7 → 297.7 $HV_{0.05}$, ↑~25%; dH ≈1600 μm; peak CRS ≈−600 MPa; dCRS ≈1500 μm	RCF life: 5.37 × 10^5 → 9.04 × 10^5 cycles, ↑~68%; plastic deformation layer: 547 → 440 μm	Initial roughness: NR; spalling length along rolling direction: 3519 → 2647 μm	Gear-steel roller RCF evidence; not an actual gear flank	[34]
Fatigue/RCF improvement	20CrNiMo steel, carburizing–LSP; GS	C-LSP; E = 2/3/4 J, τ = 20 ns, d = 3 mm, η = 50%, n = 1–3	Surface CRS: UT −56 → C −295 → C-LSP(4J)×2 −1300 MPa; dCRS ≈800 μm; hardness: ~375 → 740 → 958 HV0.1	Fatigue life: C 7.3 × 10^5 → C-LSP 3.83 × 10^6 cycles, ↑~5.25×; crack initiation shifted from surface to interior	NR	Rotating-bending fatigue evidence; not an actual gear or tooth-root specimen; RCF and roughness were not reported	[35]

Table S4. Continued. Quantitative comparison of representative LSP studies on gear steels, gear components, and related load-bearing steels.

Evidence focus	Material/evidence	Processing route and key parameters	CRS/hardening response	Performance response	Roughness/topography response	Main limitation	Ref.
Fatigue/ rolling contact fatigue (RCF) improvement	54SiCr6 high-strength spring steel; A	SAT + LSP; E = 2.25 J, τ = 17 ns, d = 1.3 mm, I = 9.98 GW/cm^2, η = 50%	CRS value: NR; quantitative hardness/microstructure after LSP: NR	3PB fatigue limit: 837 → 980 MPa at Pf50%; 751 → 944 MPa at Pf10%; crack initiation shifted from surface to interior	NR	Analogue bending-fatigue evidence; not gear steel or tooth-root specimen	[8]
Wear/friction performance	40CrMo steel; GS	LSP; E = 8 J, τ = 8 ns, d = 3 mm, η = 50%; Si_3N_4-ball reciprocating wear	Surface RS: ~+30 → −425 MPa; affected depth ≈600 μm; hardness: ~278 → 338 HV, ↑21.58%	Mass loss: 167 → 121 mg; wear-scar width: 150 → 105 μm	Roughness parameters: NR; shallower grooves and fewer debris after LSP	Gear-relevant Cr–Mo steel wear evidence; not actual gear or RCF test	[36]
Wear/friction performance	15CrNiMo carburized layer; GS	LSP: E = 5 J, d = 3 mm, η = 50%, n = 2; multi-	Peak CRS ≈−900 MPa; dCRS ≈1 mm; hardness:	COF decreased under 600/800/1000 N;	S_a slightly increased; S_{ku}: 4.12 → 2.46; S_p: 18.396	Multi-body dry-friction evidence; not actual gear,	[37]

		body dry friction	681 → 820 HV, ↑20.5%; grain size ↓14.3%	wear-scar width markedly reduced	→ 7.998 μm; peaks became blunter	RCF, or bending fatigue	
Wear/friction performance	AH32 steel; *A*	E = 1 J, τ = 8 ns, d = 2 mm, η = 50%; n = 1/2	Surface CRS: −17 → −133/−162 MPa; hardness: 190.2 → 204.7/218.3 HV; grain size ↓32.4%	COF: 0.524 → 0.464/0.427; mass loss: 42.6 → 29.3 mg	S_z: 2.428 → 6.261/9.824 μm; roughness increased but wear-scar width decreased	Marine-steel wear analogue; not gear steel	[38]

Table S4. Continued. Quantitative comparison of representative LSP studies on gear steels, gear components, and related load-bearing steels.

Evidence focus	Material/evidence	Processing route and key parameters	CRS/hardening response	Performance response	Roughness/topography response	Main limitation	Ref.
Wear/friction performance	M50 bearing steel; GS/A	LSP: E = 5 J, d = 2.2 mm, η = 50%, n = 3; dry friction at 25/300 °C	Peak CRS ≈1200 MPa; dCRS ≈2 mm; hardness ↑~14%; CRS showed thermal stability	At 300 °C, coefficient of friction (COF)↓41.4% and wear rate ↓55.8%	Worn-surface S_a: 0.152 → 0.189 μm at 25 °C; 0.376 → 0.305 μm at 300 °C	Bearing-steel high-temperature dry-friction evidence; not gear fatigue or RCF	[39]
Surface roughness/topography	laser powder bed fusion (LPBF)17-4 PH micro-spur gear; G	Water-confined LSP; E = 200 mJ, τ = 15 ns, d = 0.7 mm, η = 70%	dCRS ≈0.4 mm; RS at ~0.2 mm: +95 → −259 MPa; grain size ↓15.6%	NR	Average roughness: 12 → 8.6 μm, ↓32%; V_{mp} ↓48%; minor microgeometry change	AM micro-gear evidence; fatigue, RCF, and wear were not reported	[40]
Surface roughness/topography	AISI 9310 carburized steel; GS	LSPTwC/LSPwC; E = 90 mJ, τ = 10 ns, d = 350–750 μm	Microhardness ↑>10%; dH ≈700 μm for D3; CRS: NR	NR	Diameter deviation <3%; depth deviation <4%; higher relief near 650 μm spot diameter	Surface texturing/modeling study; fatigue, RCF, and wear were not reported	[41]
Surface roughness/topography	20CrMnTi steel; GS	Surface morphology statistics; E = 9 J, τ = 20 ns, d = 3 mm, η = 50%; single-point/multiple LSP	Single-point center CRS: ~−495 to −528 MPa; maximum after multiple impacts <−632 MPa	NR	Single-point S_a: 0.54 → 0.37/0.65/0.88/1.31 μm; multiple-impact S_a: 0.07 → 1.40–3.39 μm; S_{ku}: 11.18 → 2.54–2.89	Surface morphology statistics; hardness, fatigue, RCF, and wear were not reported	[42]

Table S4. Continued. Quantitative comparison of representative LSP studies on gear steels, gear components, and related load-bearing steels.

Evidence focus	Material/evidence	Processing route and key parameters	CRS/hardening response	Performance response	Roughness/topography response	Main limitation	Ref.
Parameter–CRS/hardening depth	20CrMnTi carburized steel; GS	E = 4–7 J; τ and n varied; optimized at 5 J, 20 ns, n = 5	Peak CRS at ≈200 μm; CRS layer ≈0.9 mm; maximum CRS ≈1085	NR	NR	FEM/XRD parameter optimization; fatigue, wear, and	[43]

			MPa and dCRS >1 mm at n = 5			roughness were not reported	
Parameter–CRS/hardening depth	40Cr steel; GS	E = 6/8/10/12 J, τ = 20 ns, d = 3 mm	Maximum CRS: −300/−359/−378/−375 MPa; dCRS: 690/810/1050/1058 μm; dH: 600–1260 μm	NR	NR	Parameter optimization; CRS/hardening gain tended to saturate at 10–12 J	[44]
Parameter–CRS/hardening depth	20Cr2Ni4 gear steel, LSP–SP; GS	FEM; LSP peak pressure = 3.0–4.5 GPa; SP velocity = 40–60 m·s^{-1}	dCRS after LSP–SP: 0.19 → 0.38 mm; dCRS was 2–3× that of SP; maximum CRS reached 1.5× that of LSP alone	NR	Roughness parameters: NR; surface deformation at 100/200/300% LSP coverage was ~2.6/4.4/6.0 μm	Numerical study; no performance experiment	[45]
FLSP/wear	AISI 9310 gear steel; GS	Heat-treated AISI 9310 + FLSP; λ = 1030 nm; τ = 290 fs; E = 50–200 μJ; d = 30 μm; pulse spacing = 10/15/30 μm; no coating/confinement	Surface CRS ↑30.5–103.1%; hardness ↑9.4–17.4%; dH ≈150–300 μm	Dry reciprocating wear; COF ↓3.4–19.0%; wear rate ↓10.1–61.7%	S_a increased; UT ≈0.085 μm; max *Sa* ≈0.188 μm; LIPSS/ablation features formed	FLSP, not conventional ns-LSP; no fatigue, RCF, lubricated contact, or gear-tooth validation	[46]

Table S4. Continued. Quantitative comparison of representative LSP studies on gear steels, gear components, and related load-bearing steels.

Evidence focus	Material/evidence	Processing route and key parameters	CRS/hardening response	Performance response	Roughness/topography response	Main limitation	Ref.
LSPwC/surface texturing	AISI 9310 gear steel; GS	C + quenching + cryogenic treatment + LSPwC; λ = 532 nm; τ = 10 ns; E = 30–90 mJ; d = 500 μm; spot spacing = 1000 μm; no coating	Peak CRS ≈488 MPa at ≈40 μm; dCRS ≈180 μm; dH ≈700 μm; hardness ↑9.7%	NR; dry-running wear-risk-oriented surface modification	Micro-dimple depth ≈1.5–3.0 μm; R_a ≈0.02 → 0.15–0.35 μm	LSPwC texturing/FEM study; no fatigue, RCF, wear test, or actual gear validation	[47]
LSP / tribological response	M50 bearing steel; A	Heat-treated M50 + LSP; λ = 532 nm; τ = 10 ns; E = 30/50/70/90 mJ; d = 0.4 mm; η ≈50%; water confinement; no coating	At 70 mJ: surface CRS = −547 MPa; dCRS ≈200 μm; peak hardness = 11.75 GPa; excessive E caused carbide damage	Dry ball-on-disk wear; at 70 mJ: COF 0.738 → 0.474, ↓35.77%; wear loss 6.431 → 1.047 mg, ↓83.72%	S_a after polishing = 0.08–0.14 μm; max wear-track depth 13.23 → 5.39 μm at 70 mJ	Recent carbide-bearing bearing-steel analogue; dry sliding wear only; no gear steel/component, fatigue, RCF, or lubricated-contact validation	[48]

Notes:

G = actual gear or gear-like component evidence; GS = gear steel or gear-relevant simplified-specimen evidence; A = analogue-material evidence.
UT = untreated; C = carburizing; C-LSP = carburizing + LSP; SP = shot peening; SAT = strain-assisted tempering; CRS = compressive residual stress; RS = residual stress; RCF = rolling contact fatigue; COF = coefficient of friction; NR = not reported in the original study.
E = pulse energy; τ = pulse duration; d = spot diameter; η = overlap ratio; n = number of impacts; I = power density; dCRS = CRS-affected depth; dH = hardened-layer depth.
"→" indicates evolution from untreated/baseline to treated state; "↑/↓" indicates increase/decrease; "×" indicates fold change; "≈" indicates approximate value.

E = pulse energy; τ= pulse duration; d= spot diameter; η= overlap ratio; n= number of impacts; I= power density; λ= laser wavelength; E_S= residual-stress uniformity coefficient; E_P= plastic-strain uniformity coefficient; p_{max}= maximum point-contact pressure; P_f= failure probability; d_{CRS}= CRS-affected depth; d_H= hardened-layer depth; R_a= arithmetical mean roughness; S_a= arithmetical mean height; S_q= root mean square height; S_{ku}= surface kurtosis; S_p= maximum peak height; S_z= maximum height; V_{mp}= peak material volume.

Table S5. Typical hybrid routes, dominant mechanisms, representative results, and limitations of pre-LSP-assisted diffusion enhancement and case-layer formation.

Material system	Hybrid route	Strengthening mechanism	Representative result	Main limitation / processing window	Ref.
42CrMo steel	laser shock peening followed by plasma nitriding (LSP-PN)	Surface activation and near-surface defect channels jointly promote nitriding	Compound layer thickness increased from 6.25 μm to 13.04 μm; effective hardened layer increased from 120 μm to 230 μm	Surface roughness increased from 0.015 μm to 0.454 μm	[49]
20Cr2Mn2Mo steel	laser shock peening followed by vacuum carbonitriding (LSP-VCN)	Dislocation proliferation and second-phase strengthening jointly promote vanadium carbonitriding, accompanied by residual stress redistribution and changes in wear mechanism	Hardened layer increased from about 170 μm to about 320 μm; wear rate decreased by 29% compared with carbonitriding alone	Excessive energy or impact number may increase COF	[50]
AISI 9310 steel	LSP–low-temperature carburizing	Defect-channel construction and reduced activation energy for carbon diffusion	Carburized layer thickness increased from about 10 μm to about 34 μm, with a deeper work-hardened layer	Thermal stability still requires further verification	[51]
20Cr2Mn2Mo steel	LSP-VCN	Gradient microstructure, dislocations and second phases act synergistically	Yield strength and tensile strength increased by 10.63% and 4.15%, respectively	Plasticity decreased by 0.99%	[52]
M50 steel	LSP-PN	Dislocation-assisted N diffusion with reticulated-structure weakening	Nitrided layer increased to 433.6 μm; hardened layer reached 520 μm; wear resistance improved	Reticulated structure was reduced but not completely eliminated	[53]
M50 steel	laser shock peening followed by nitrogen ion	Synergy between deep compressive stress and shallow amorphization	Amorphous layer thickened from about 20 nm to about 33 nm; nanohardness increased to 12.62 GPa; surface hardness	—	[54]

	implantation (LSP-NII)		increased from about 995 HV to 1307 HV		

Table S5. Continued. Typical hybrid routes, dominant mechanisms, representative results, and limitations of pre-LSP-assisted diffusion enhancement and case-layer formation.

Material system	Hybrid route	Strengthening mechanism	Representative result	Main limitation / processing window	Ref.
H13 steel	LSP-PN	Dislocation cells and nanocrystals promote plasma nitriding	White/compound layer increased from 2.37 μm to 5.20 μm; effective hardened layer increased from 80 μm to 120 μm	—	[55]
17-4PH stainless steel	LSP-PN	Grain refinement and dead-zone suppression promote nitriding	Effective hardened layer thickness increased by about 115%	A 40% overlap was optimal; obvious surface roughening occurred at 80% overlap	[56]

https://doi.org/10.3390/ma19153257

Table S6. Reconstruction routes and strengthening responses of pre-existing diffusion cases after post-LSP.

Material system	Hybrid route	Strengthening mechanism	Representative results	Main limitation/process window	Ref.
20CrNiMo	Quenching and Tempering–Carburizing Treatment–Laser Shock Peening (QT–CT–LSP)	Surface refinement, CRS enhancement, and phase-constitution adjustment.	Surface hardness increased to 916 $HV_{0.1}$, surface CRS reached −635 MPa, and retained austenite transformed into martensite.	—	[1]
20CrNiMo	CT–LSP	High hardness, deep compressive stress, and surface refinement jointly suppress crack initiation and growth.	Fatigue life was approximately 5.25 times higher than that of the carburized sample, and the crack origin shifted from surface machining marks to subsurface or internal inclusions.	The benefit tended to saturate with increasing laser energy and number of impacts.	[35]
EX30	CT–LSP	Compositional and nanocrystalline gradients jointly disperse strain.	Wear rates decreased by 53.9% and 54.3%, with a clearer gradient strain distribution retained.	—	[57]
AISI 321	Aluminizing–LSP	Dislocation and residual-stress gradients are superimposed on the chemically graded layer.	At 620 °C, the yield strength, tensile strength, and elongation after fracture reached 265 MPa, 430 MPa, and 37.5%, respectively.	Aluminizing alone can introduce pores or defects, which reduce high-temperature tensile performance.	[58]

Table S7. Representative routes, strengthening mechanisms, and main results/limitations of synergistic strengthening by mechanical pre-deformation and LSP.

Material system	Hybrid route	Main mechanism	Representative results	Main boundary/limitation	Ref.
20Cr2Ni4 gear steel	Shot Peening–Laser Shock Peening (SP–LSP)	Coupled development of a mechanically strengthened layer and a deep CRS layer.	When the peak pressure increased from 3.0 to 4.5 GPa, the CRS depth increased from 0.19 to 0.38 mm.	Once the shot-peening coverage exceeded 100%, further increases in maximum CRS and affected depth became limited.	[45]
30CrMnSiA medium-carbon steel	SP–LSP	Ultrafine grains, dislocation tangles, and high-angle grain boundaries jointly impeded short-crack growth.	With 478 MPa as the transition stress, SP + LSP produced longer fatigue life than SP in the low-stress regime, whereas SP was more favorable in the high-stress regime.	The hybrid benefit did not extend across the full stress range.	[59]
SLM Ti–6Al–4V alloy	LSP–UNSM /UNSM–LSP	Sequence-dependent smoothing, plastic deformation, CRS formation, and hardening.	Roughness decreased to about 1.5–1.6 μm; surface hardness increased to about 364–367 HV; wear resistance improved over untreated and LSP-only states.	Non-steel analogue; sequence effect requires validation in gear steels.	[24]
GH5188 Co-based superalloy	Laser Shock Peening–Ultrasonic Surface Rolling (LSP–USR)	Deep LSP deformation and surface USR refinement jointly promoted gradient structure, dislocations, nanotwins, and HDI strengthening.	Gradient depth ≈400 μm; surface hardness ↑108.9%; YS ↑31.5%; UTS ↑10.4%; EL remained 48.5%.	Non-gear superalloy analogue; fatigue, RCF, wear, and gear validation were not reported.	[60]

https://doi.org/10.3390/ma19153257

Table S8. Representative routes, strengthening mechanisms, and main results/limitations of LSP-based re-regulation after heat treatment.

Material system	Hybrid route	Strengthening mechanism	Representative results	Main limitation/applicability boundary	Ref.
Cr12MoV steel	Laser Quenching–Laser Shock Peening (LQ–LSP)	Phase-transformation regulation and compressive-stress optimization superimposed on a martensitic hardened layer.	Surface hardness increased to 722.3 HV, the peak surface CRS reached −383.84 MPa, and the retained-austenite content decreased to 0.8%.	Three impacts gave the optimal response.	[61]
14Cr12Ni3Mo2VN steel	High-Frequency Induction Quenching–Laser Shock Peening (HFIQ–LSP)	Gradient microstructure and stress field developed on the quenched layer.	Surface hardness reached 524 $HV_{0.1}$, and the cavitation fatigue life increased by 2.88 times.	The benefit depends on the synergy between the gradient microstructure and compressive stress.	[62]

References

1. Chen, X.; Sun, J.; Li, K.; Lin, Y.; Xu, Z.; Guo, B.; Chen, J.; Jiang, Q. Effect of Carburizing Composite Laser-Shock Processing on Properties and Microstructure of 20CrNiMo Steel. *Metals* **2024**, *14*, 10, doi:10.3390/met14010010.
2. Wang, H.; Peng, C.; Xiao, Y. "Influence of Residual Stress on Fatigue Lives of AISI9310 Gear Processed by Laser Shock Peening," 2019 IEEE International Conference on Mechatronics and Automation (ICMA), Tianjin, China, **2019**, pp. 728–732, doi:10.1109/ICMA.2019.8816407.
3. Raj, D.; Tyagi, R.; Chaubey, S.K.; D'Souza, A.; Palani, I.A.; Jain, N.K. Investigation on Solid State Nd-YAG nanosecond Laser Assisted Shock Peening of Miniature Gears. *Materials Today: Proceedings* **2015**, *2*, 1755–1762, doi:10.1016/j.matpr.2015.07.012.
4. Deng, W.; Wang, C.; Lu, H.; Xu, X.; Luo, K.; Lu, J. Fatigue crack initiation and growth of laser shock peened 2Cr13 martensitic stainless steel as a function of the coverage layer. *International Journal of Fatigue* **2023**, *175*, 107792, doi:10.1016/j.ijfatigue.2023.107792.
5. Takahashi, K.; Kogishi, Y.; Shibuya, N.; Kumeno, F. Effects of laser peening on the fatigue strength and defect tolerance of aluminum alloy. *Fatigue & Fracture of Engineering Materials & Structures* **2020**, *43*, 845–856, doi:10.1111/ffe.13201.
6. Attolico, M.A.; Barile, C.; Casavola, C.; Moramarco, V.; Furfari, D.; Busse, D.O. Effects of Laser Shock Peening on Surface Roughness and Residual Stress of AA 7050-T7451. *Journal of Materials Engineering and Performance* **2022**, *31*, 7973–7988, doi:10.1007/s11665-022-06857-7.
7. Schubnell, J.; Carl, E.-R.; Sarmast, A.; Hinterstein, M.; Preußner, J.; Seifert, M.; Kaufmann, C.; Rußbüldt, P.; Schulte, J. Surface Conditions after LASER Shock Peening of Steel and Aluminum Alloys Using Ultrafast Laser Pulses. *Materials* **2023**, *16*, 6769, doi:10.3390/ma16206769.
8. Procházka, R.; Stehlík, A.; Kotous, J.; Salvetr, P.; Bucki, T.; Stránský, O.; Zulić, S. Fatigue Properties of Spring Steels after Advanced Processing. *Materials* **2023**, *16*, 3327, doi:10.3390/ma16093327.
9. Yang, F.; He, P.; Wang, W.; Yu, Y.; Pan, X.; Yang, H.; Liang, X.; Xie, C.; Zhou, L. A duplex surface modification method for increasing bending fatigue life of aviation carburized gear steel considering its mechanical properties and microstructure gradients. *Journal of Materials Processing Technology* **2025**, *339*, 118812, doi:10.1016/j.jmatprotec.2025.118812.
10. Sun, R.; Huang, W.; Cao, Z.; Che, Z.; Zou, S.; Wu, J. Fatigue life extension of 42CrMo high-strength steel and its micro strengthening mechanism under laser shock peening. *IOP Conference Series: Materials Science and Engineering* **2020**, *892*, 012005, doi:10.1088/1757-899X/892/1/012005.
11. Shu, S.; Shen, Y.; Cheng, Z.; Xiong, W.; He, Z.; Song, S.; Liu, W. Laser shock peening regulating residual stress for fatigue life extension of 30CrMnSiNi2A high-strength steel. *Optics & Laser Technology* **2024**, *169*, 110094, doi:10.1016/j.optlastec.2023.110094.
12. Chen, H.; Dou, M.; Li, L.; Huang, W.; Li, W.; Zhao, D.; Sun, S. Plastic deformations of 42CrMo4 under LSPwC and subsequent cyclic stresses. *International Journal of Mechanical Sciences* **2023**, *259*, 108623, doi:10.1016/j.ijmecsci.2023.108623.

13. Soyama, H. Comparison between Shot Peening, Cavitation Peening, and Laser Peening by Observation of Crack Initiation and Crack Growth in Stainless Steel. *Metals* **2020**, *10*, 63, doi:10.3390/met10010063.
14. Yang, J.; Chen, J.; Liu, Y.; Sun, B.; Zhu, R. The effect of laser shock peening on the fatigue performance of 25Cr2Ni4MoV alloy. *Engineering Failure Analysis* **2025**, *169*, 109150, doi:10.1016/j.engfailanal.2024.109150.
15. Xie, X.; Zhang, L.; Zhu, L.; Li, Y.; Hong, T.; Yang, W.; Shan, X. State of the Art and Perspectives on Surface-Strengthening Process and Associated Mechanisms by Shot Peening. *Coatings* **2023**, *13*, 859, doi:10.3390/coatings13050859.
16. Wildeis, A.; Christ, H.-J.; Brandt, R. Influence of Residual Stresses on the Crack Initiation and Short Crack Propagation in a Martensitic Spring Steel. *Metals* **2022**, *12*, 1085, doi:10.3390/met12071085.
17. Wang, Z.Y.; Yang, X.F.; Zhang, Z.Q.; Zhu, Y.Q.; Song, F.; Dong, W.L.; Wu, M. Study on shot peening strengthening 42CrMoA steel and its fretting wear properties. *Materials Science and Technology* **2025**, *41*, 1431–1441, doi:10.1177/02670836241282770.
18. Qian, W.; Wang, Y.; Liu, K.; Yin, X.; He, X.; Xie, L. Experimental Study on the Effect of Shot Peening and Re-Shot Peening on the Residual Stress Distribution and Fatigue Life of 20CrMnTi. *Coatings* **2023**, *13*, 1210, doi:10.3390/coatings13071210.
19. Wang, C.; Fan, K.; Li, C.; Abdel Wahab, M. Prediction of the effect of shot peening residual stress on fretting fatigue behaviour. *International Journal of Fatigue* **2023**, *176*, 107909, doi:10.1016/j.ijfatigue.2023.107909.
20. Xu, C.; Wang, X.; Zhang, B.; Luo, X.; Tang, Z.; Dai, S. Effect of the Pre-Shot Peening and Nitrogen Ion Implantation Combined Surface Treatments on the Surface Structure and Properties of Gear Steel 16Cr3NiWMoVNbE. *Metals* **2022**, *12*, 1509, doi:10.3390/met12091509.
21. Dang, J.; Zhang, H.; An, Q.; Lian, G.; Li, Y.; Wang, H.; Chen, M. Surface integrity and wear behavior of 300M steel subjected to ultrasonic surface rolling process. *Surface and Coatings Technology* **2021**, *421*, 127380, doi:10.1016/j.surfcoat.2021.127380.
22. Liu, C.; Liu, D.; Zhang, X.; Yu, S.; Zhao, W. Effect of the Ultrasonic Surface Rolling Process on the Fretting Fatigue Behavior of Ti-6Al-4V Alloy. *Materials* **2017**, *10*, 833, doi:10.3390/ma10070833.
23. Wang, H.; Wang, X.; Tian, Y.; Ling, Y. Study on microstructure of 42CrMo steel by ultrasonic surface rolling process. *Scientific Reports* **2023**, *13*, 21289, doi:10.1038/s41598-023-48697-y.
24. Amanov, A.; Yeo, I.K.; Jeong, S.H. Advanced post-processing of Ti–6Al–4V alloy fabricated by selective laser melting: A study of laser shock peening and ultrasonic nanocrystal surface modification. *Journal of Materials Research and Technology* **2025**, *35*, 4020–4031, doi:10.1016/j.jmrt.2025.02.038.
25. Soyama, H.; Korsunsky, A.M. A critical comparative review of cavitation peening and other surface peening methods. *Journal of Materials Processing Technology* **2022**, *305*, 117586, doi:10.1016/j.jmatprotec.2022.117586.
26. Noronha, D.J.; Sharma, S.; Prabhu Parkala, R.; Shankar, G.; Kumar, N.; Doddapaneni, S. Deep Rolling Techniques: A Comprehensive Review of Process Parameters and Impacts on the Material Properties of Commercial Steels. *Metals* **2024**, *14*, 667, doi:10.3390/met14060667.
27. Schubnell, J.; Farajian, M. Fatigue improvement of aluminium welds by means of deep rolling and diamond burnishing. *Welding in the World* **2021**, *66*, 699–708, doi:10.1007/s40194-021-01212-1.
28. Pujono; Ilman, M.N.; Kusmono; Muslih, M.R.; Priyanto, T.H.; Apriansyah, R.; Isnaini, A. Diminishing residual stress and distortion by in-situ rolling tensioning to increase fatigue performance of friction stir welded AA2024-T3 joints. *International Journal of Fatigue* **2025**, *190*, 108659, doi:10.1016/j.ijfatigue.2024.108659.
29. Wang, W.; Liu, H.; Zhu, C.; Du, X.; Tang, J. Effect of the residual stress on contact fatigue of a wind turbine carburized gear with multiaxial fatigue criteria. *International Journal of Mechanical Sciences* **2019**, *151*, 263–273, doi:10.1016/j.ijmecsci.2018.11.013.
30. Ooi, G.T.C.; Roy, S.; Sundararajan, S. Investigating the effect of retained austenite and residual stress on rolling contact fatigue of carburized steel with XFEM and experimental approaches. *Materials Science and Engineering: A* **2018**, *732*, 311–319, doi:10.1016/j.msea.2018.06.078.
31. Sommer, M.; Sitzmann, A.; Hantzsche, K.; Tobie, T.; Stahl, K.; Fechte-Heinen, R.; Hoja, S. Optimized Compound Layer Design for Highly Loaded Nitrided Gears. *Journal of Materials Engineering and Performance* **2024**, *33*, 13293–13310, doi:10.1007/s11665-024-09659-1.
32. Peng, C.; Xiao, Y.; Wang, Y.; Guo, W. Effect of laser shock peening on bending fatigue performance of AISI 9310 steel spur gear. *Optics & Laser Technology* **2017**, *94*, 15–24, doi:10.1016/j.optlastec.2017.03.017.
33. Chen, X.; Li, K.; Lin, Y.; Xu, Z.; Guo, B.; Chen, J.; Jiang, Q.; Zhao, Z. Effect of laser shock peening uniformity on bending fatigue life of 20CrNiMo. *Optics & Laser Technology* **2024**, *175*, 110704, doi:10.1016/j.optlastec.2024.110704.
34. Song, J.; Luo, S.; Liang, X.; Cao, Z.; Zhao, W.; Pu, C.; He, W. Rolling contact fatigue and damage characteristic of AISI 9310 steel with pre-laser shock peening treatment. *International Journal of Fatigue* **2022**, *155*, 106588, doi:10.1016/j.ijfatigue.2021.106588.

35. Chen, X.; Jiang, W.; Sun, J.; Li, Y.; Ma, W.; Xu, Z.; Jiang, Q.; Chen, J.; Li, Y. The effects of composite surface modification on the bending fatigue properties of 20CrNiMo steel. *Optics & Laser Technology* **2025**, *180*, 111566, doi:10.1016/j.optlastec.2024.111566.
36. Pan, X.; Gu, Z.; Qiu, H.; Feng, A.; Li, J. Study of the Mechanical Properties and Microstructural Response with Laser Shock Peening on 40CrMo Steel. *Metals* **2022**, *12*, 1034, doi:10.3390/met12061034.
37. Wang, Y.; Wang, G.; Liu, W.; Liu, Q.; Hou, D.; Zhong, L.; Jin, Z.; Xu, Z.; Wei, G. Effect of laser shock peening on tribological performance of 15CrNiMo carburized layer in multi-body friction systems. *Journal of Materials Research and Technology* **2025**, *37*, 5369–5386, doi:10.1016/j.jmrt.2025.07.142.
38. Pei, X.; Shen, Y.; Xu, Z.; Li, P.; Peng, Y. The Influence of Laser Shock Peening on the Microstructure and Mechanical Properties of AH32 Steel. *Materials* **2025**, *18*, 4679, doi:10.3390/ma18204679.
39. Cao, Z.; Cui, L.; Luo, S.; Song, J.; Su, H.; Pang, Z.; Zhao, W.; He, W.; Liang, X. Insights into room- and elevated-temperature micro-mechanisms of laser shock peened M50 steel with superior tribological performance. *Journal of Materials Science & Technology* **2025**, *205*, 270–285, doi:10.1016/j.jmst.2024.04.039.
40. Pathak, S.; Stránský, O.; Šmaus, J.; Kopeček, J.; Narayanan, J.A.; Kaufman, J.; Beránek, L.; Böhm, M.; Brajer, J.; Mocek, T. Influence on micro-geometry and surface characteristics of laser powder bed fusion built 17-4 PH miniature spur gears in laser shock peening. *Advances in Industrial and Manufacturing Engineering* **2024**, *9*, 100151, doi:10.1016/j.aime.2024.100151.
41. Liu, P.; Yang, Z.; Xie, C.; Yang, F.; Zhou, L. Control and Modelling of Laser Shock Peening without Coating (LSPwC) Texture of AISI 9310 Steel. *Materials* **2024**, *17*, 4776, doi:10.3390/ma17194776.
42. Wu, J.; Zhang, D.; Wang, X. Surface Morphology Analysis of Laser Shock Peened 20CrMnTi Steel: A Statistical Evaluation. *Metals* **2023**, *13*, 1673, doi:10.3390/met13101673.
43. Sun, J.; Li, J.; Chen, X.; Xu, Z.; Lin, Y.; Jiang, Q.; Chen, J.; Li, Y. Optimizing Parameters with FEM Model for 20CrMnTi Laser Shocking. *Materials* **2023**, *16*, 328, doi:10.3390/ma16010328.
44. Chen, X.; Sun, J.; Xu, Z.; Chen, J.; Jiang, Q.; Li, Y.; Li, J.; Cheng, J. Optimization of Laser Shock Process Parameters for 40Cr Steel. *Coatings* **2022**, *12*, 1872, doi:10.3390/coatings12121872.
45. He, G.; Xie, Y.; Zou, S.; Peng, L.; Xu, W. Numerical investigation on process parameters of compound treatment of laser shock peening and shot peening on the effect of the mechanical properties of 20Cr2Ni4 gear steel. *The International Journal of Advanced Manufacturing Technology* **2025**, *140*, 4003–4018, doi:10.1007/s00170-025-16392-z.
46. Yang, F.; Zhao, T.; He, P.; Zhou, L.; Pan, X.; Liang, X.; Jia, W.; An, Z.; Liu, P.; Zhang, H. Effect of pulsed femtosecond laser shock peening surface modification on anti-wear failure properties of AISI 9310 gear steel. *Engineering Failure Analysis* **2024**, *159*, 108146, doi:10.1016/j.engfailanal.2024.108146.
47. Yang, Z.; Liu, P.; Zhou, J.; Wang, H. Investigation of the Thermo-Mechanical Coupling Mechanism in Surface Texturing of AISI 9310 Steel Through Laser Shock Peening Without Coating. *JOM* **2025**, *77*, 9842–9864, doi:10.1007/s11837-025-07801-5.
48. Ma, Y.; Jiang, H.; Zou, H.; Yan, S.; Shao, B.; Zong, Y. Carbide-mediated martensite nanostructuring enabling tribological performance improvement under laser shock peening. *Journal of Materials Processing Technology* **2026**, *354*, 119379, doi:10.1016/j.jmatprotec.2026.119379.
49. Tang, L.; Jia, W.; Hu, J. An enhanced rapid plasma nitriding by laser shock peening. *Materials Letters* **2018**, *231*, 91–93, doi:10.1016/j.matlet.2018.08.010.
50. Ren, Y.; Wan, H.; Chen, Y.; Zhu, H.; Lu, H.; Ren, X. Effect of laser shock peening and carbonitriding on tribological properties of 20Cr2Mn2Mo steel alloy under dry sliding conditions. *Surface and Coatings Technology* **2021**, *417*, 127215, doi:10.1016/j.surfcoat.2021.127215.
51. Song, J.; He, W.; Liang, X.; Cao, Z.; Pang, Z.; Zhao, W.; Luo, S. Enhanced carbon diffusion efficiency and work hardening effect of AISI 9310 steel via pre-laser shock peening. *Surface and Coatings Technology* **2023**, *473*, 129932, doi:10.1016/j.surfcoat.2023.129932.
52. Wan, H.; Lu, H.; Ren, Y.; Ma, C.; Chen, Y.; Xin, Z.; Cheng, L.; He, K.; Tu, X.; Han, Q.; et al. Strengthening mechanisms and tensile properties of 20Cr2Mn2Mo processed by laser shock peening and vacuum carbonitriding. *Surface and Coatings Technology* **2022**, *439*, 128462, doi:10.1016/j.surfcoat.2022.128462.
53. Sun, Y.; Liu, W.; Yu, X.; Wei, Y.; Su, Y.; Wang, Q.; Na, Y. Effect of Compound Treatment of Laser Shock Peening and Nitriding on Wear Resistance of M50 Steel. *Journal of Materials Engineering and Performance* **2024**, *34*, 10591–10600, doi:10.1007/s11665-024-09839-z.
54. Cao, Z.; He, W.; Luo, S.; Song, J.; Su, H.; Pang, Z.; Zhao, S.; Liang, X. Effect of combined laser shock peening and nitrogen implantation on the microstructure and tribology of M50 bearing steel. *Journal of Materials Research and Technology* **2024**, *30*, 5080–5095, doi:10.1016/j.jmrt.2024.04.213.

55. Luo, J.; Wang, J.; Xu, J. Effect of laser shock peening on plasma nitriding microstructure and properties of H13 steel. *Surface and Coatings Technology* **2023**, *473*, 130004, doi:10.1016/j.surfcoat.2023.130004.
56. Avinash, S.; Yadav, V.K.; Duraiselvam, M. Effect of laser shock peening as a pretreatment on ion nitriding of precipitation hardened stainless steel. *Surface Topography: Metrology and Properties* **2023**, *12*, doi:10.1088/2051-672X/ad1607.
57. Wang, Y.; Wang, G.; Yan, J.; Liao, Y.; He, X.; Zhong, L.; Wei, G.; Xu, Z. Investigation of the effects of a laser-induced integrated gradient structure on the wear resistance of EX30 bearing steel. *Journal of Materials Research and Technology* **2026**, *40*, 1003–1020, doi:10.1016/j.jmrt.2025.12.143.
58. Yu, L.; Jiang, W.; An, X.; Ni, S.; Bo, G.; Li, W.; Song, M. Introducing both chemical and structural gradients in AISI 321 stainless steel via aluminizing and laser shock peening for superior properties. *Surface and Coatings Technology* **2023**, *472*, 129942, doi:10.1016/j.surfcoat.2023.129942.
59. Zhang, Y.; Zhang, K.; Hu, Z.; Chen, T.; Susmel, L.; Wei, B. The synergetic effects of shot peening and laser-shot peening on the microstructural evolution and fatigue performance of a medium carbon steel. *International Journal of Fatigue* **2023**, *166*, 107246, doi:10.1016/j.ijfatigue.2022.107246.
60. Zhang, W.; Bu, X.; Deng, W.; Bao, J.; Meng, X.; Luo, K.; Lu, J. Gradient structure in GH5188 alloy fabricated by compound treatment of laser shock peening and ultrasonic surface rolling for synergistic improvement of strength and plasticity. *Materials Science and Engineering: A* **2026**, *970*, 150469, doi:10.1016/j.msea.2026.150469.
61. Feng, A.; Zhao, J.; Lin, J.; Pan, X.; Feng, H.; Wang, C.; Lu, Z. Effect of Laser Quenching-Shock Peening Strengthening on the Microstructure and Mechanical Properties of Cr12MoV Steel. *Materials* **2022**, *15*, 6693, doi:10.3390/ma15196693.
62. Gao, J.; Xu, Z.; Lv, H.; Yang, Z.; Yang, G.; Liu, L. Study on Microstructure and Corrosion Fatigue Resistance of 14Cr12Ni3Mo2VN Materials Based on the Composite Technology of High-Frequency Induction Quenching and Laser Shock Peening. *Coatings* **2024**, *14*, 1484, doi:10.3390/coatings14121484.

Disclaimer/Publisher's Note: The statements, opinions and data contained in all publications are solely those of the individual author(s) and contributor(s) and not of MDPI and/or the editor(s). MDPI and/or the editor(s) disclaim responsibility for any injury to people or property resulting from any ideas, methods, instructions or products referred to in the content.
